# Synthesis and Characterization of Phosphanophenolate-Based Rare-Earth Metal–Copper Complexes

**DOI:** 10.1021/acs.inorgchem.5c01738

**Published:** 2025-08-28

**Authors:** Andreas Fleißner, Viktoria Rehbein, Alexandra Haidinger, Christina I. Dilly, Antoine Dupé, Roland C. Fischer, Elise S. Hecht, Johann A. Hlina

**Affiliations:** † 27267 Institute of Chemistry, Inorganic Chemistry, University of Graz, Schubertstraße 1, 8010 Graz, Austria; ‡ 27253 Institute of Inorganic Chemistry, Graz University of Technology, Stremayrgasse 9, 8010 Graz, Austria; § Institute of Analytical Chemistry and Food Chemistry, Graz University of Technology, Stremayrgasse 9, 8010 Graz, Austria

## Abstract

The reaction of [Ln^III^(OAr^P^-κ^2^ *O*,*P*)_3_] (**1-Ln**, Ln = La, Sm, Y, Yb, and Ar^P^O^–^ = 2,4-^t^Bu_2_-6-(Ph_2_P)­C_6_H_2_O^–^) with the copper­(I) triflate toluene adduct yields the corresponding dinuclear rare-earth metal­(III)–copper­(I) complexes [Ln^III^(OTf)­(μ-OAr^P^-1κ^1^ *O*,2κ^1^ *P*)_3_Cu^I^] (**2-Ln**, Ln = La, Sm, Y, Yb) in clean conversion. In order to explore an alternative synthetic route by starting from a mononuclear copper complex instead of a rare-earth metal complex, the mononuclear copper complex [(HOAr^P^-κ*P*)_3_Cu^I^OTf] (**3**) was prepared from copper­(I) triflate toluene adduct and HOAr^P^. However, the reaction of **3** with [Ln^III^[N­(SiMe_3_)_2_]_3_] (Ln = La, Y) gave the heterobimetallic complexes only in low yields, along with undesired side-products. Investigation into the exchange of the triflate in **2-Ln** for different groups was explored using alkaline metal amides, alkyls, and alkoxides and was successful only for reactions with KO^t^Bu, yielding [Ln^III^(O^t^Bu)­(μ-OAr^P^-1κ^1^ *O*,2κ^1^ *P*)_3_Cu^I^] (**4-Ln**, Ln = La, Sm, Y, Yb). The compounds were characterized by NMR, UV–vis, and IR spectroscopy, single-crystal X-ray diffraction, and elemental analysis. The effective magnetic moments of the paramagnetic complexes were determined via the Evans NMR method.

## Graphical abstract

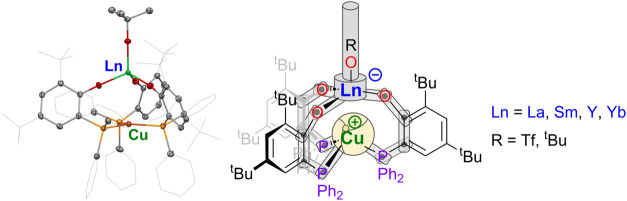

## Introduction

The interaction between metals in multinuclear, molecular compounds has been studied extensively for the transition metals.^1,2^ The interest to combine different properties within the same complexes sparked the research into combinations of early and late transition metals and revealed reactivity beyond the scope of the individual metals valuable for application in areas such as catalysis.^2–5^ This contrasts the situation of combinations of transition metals with f-block metals, as the chemistry of d–f–metal complexes received significantly less attention and is still poorly understood in comparison.^6^ Among the key contributions to the field is the work by the Kempe group, who reported the first examples of unsupported bonds between rare-earth and transition metals (Figure 1).^7–10^ These were realized by utilizing metalloligands, e.g., bis­(cyclopentadienyl)­rhenate, Cp_2_Re^–^, to form complexes such as [Cp_2_ReLnCp_2_] (Ln = Y, Yb), [(Cp_2_Re)_3_Ln] (Ln = La, Sm, Lu), and [(Cp_2_Re)_2_Yb­(THF)_2_]. In the context of catalytic applications, the use of supporting ligands to provide a more robust bonding situation appears feasible, as this allows a wider range of transformations at the individual metals, while keeping the metals within close proximity of each other. Roesky and his group demonstrated the use of supporting amidophosphane ligands to form di- and trinuclear rare-earth-group 10 metal complexes with short intermetallic distances (Figure 1).^11,12^ The first catalytic application of rare-earth transition metal complexes was presented by Lu and co-workers for the hydrogenation of alkynes (Figure 1).^13^ The authors showed that the catalyst performance may be tuned by combining nickel with different rare-earth metals or gallium. The Cui group reported similar heterodinuclear scandium-group 10 metal complexes employing similar ligands and recently showed the catalytic application of a scandium–nickel system in the hydrosilylation of terminal alkenes (Figure 1).^14–17^ In addition to the amidophosphane ligands, phosphanophenolate-based systems have been used to support complexes combining transition with f-block metals.^18–21^ More recently, Ortu and co-workers enhanced our understanding of the coordination chemistry of mononuclear phosphanophenolate-based rare-earth metal complexes using (2-di-*tert*-butyl)- and (2-di-*iso*-propyl)­phenol as proligands.^22^ Building on our previous report on rare-earth metal–silver complexes (Figure 1), we would like to present the results of our research on heterobimetallic rare-earth metal–copper complexes.^21^

**Figure 1:**
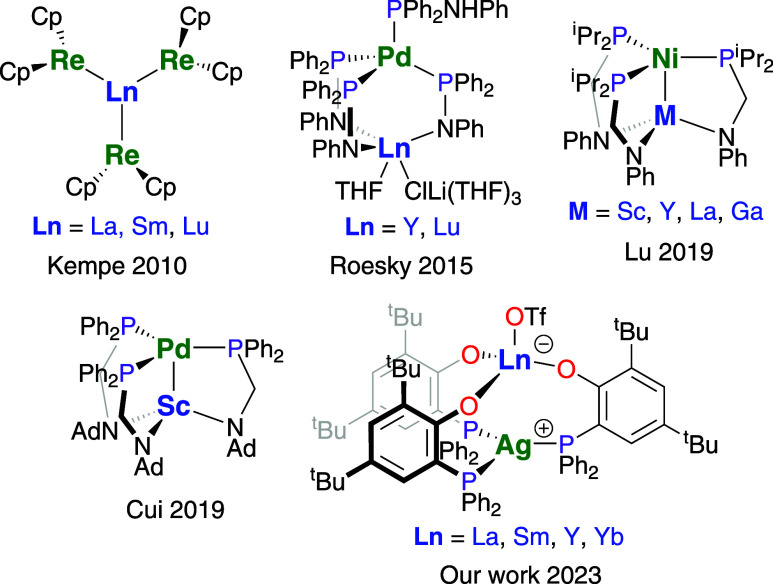
Selected examples of previously reported heterobimetallic rare-earth-transition metal complexes.

## Results and Discussion

### Synthesis and Characterization

In adaptation of our previous research into the chemistry of rare-earth metal–silver complexes, we treated mononuclear rare-earth metal complexes [Ln^III^(OAr^P^-κ^2^ *O*,*P*)_3_] (**1-Ln**, Ln = La, Sm, Y, Yb, Ar^P^O^–^ = 2,4-^t^Bu_2_-6-(Ph_2_P)­C_6_H_2_O^–^) with equimolar amounts of copper­(I) triflate toluene adduct in benzene or toluene at ambient temperature (Scheme 1).^21^ This gave the dinuclear rare-earth metal­(III)–copper­(I) complexes [Ln^III^(OTf)­(μ-OAr^P^-1κ^1^ *O*,2κ^1^ *P*)_3_Cu^I^] (**2-Ln**, Ln = La, Sm, Y, Yb) in yields of 33–89% after crystallization from THF/pentane at ambient temperature as colorless (**2-La** and **2-Y**), pale yellow (**2-Sm**), or amber (**2-Yb**) solids. The NMR spectroscopic analyses showed clean and quantitative conversion; however, the conditions for the crystallization of the compounds were not optimized, resulting in a wide range of isolated yields. Using the same synthetic conditions, we found that bis­(1,5-cyclooctadiene)­copper­(I) triflate could also be employed as an alternative copper­(I) triflate source. However, copper­(I) triflate toluene adduct already gave good results and was synthetically more feasible, as bis­(1,5-cyclooctadiene)­copper­(I) triflate is typically prepared from the former.^23^ In adaptation of a previously reported method, we prepared bis­(1,5-cyclooctadiene)­copper­(I) triflate by treatment of copper­(I) triflate toluene adduct with two equiv of 1,5-cyclooctadiene in toluene at ambient temperature in 92% yield.

**Scheme 1:**
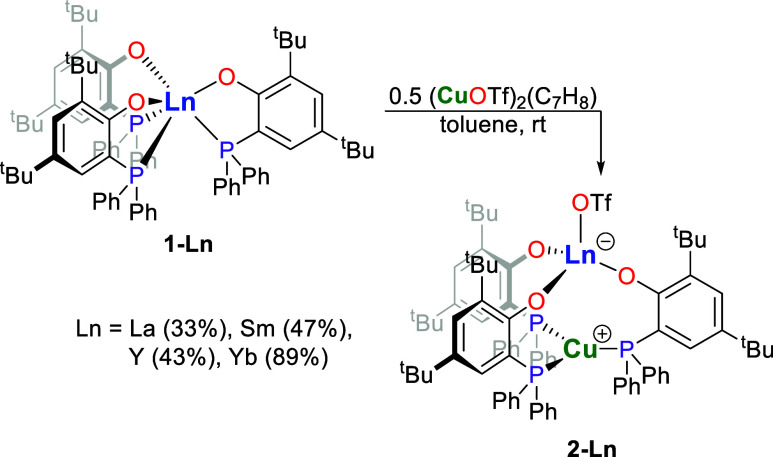
Synthesis of the Phosphanophenolate-Bridged Rare-Earth Metal­(III)–Copper­(I) Triflate Complexes (**2-Ln**, Ln = La, Sm, Y, Yb)

Crystals of heterobimetallic complex **2-Ln** suitable for single-crystal X-ray diffraction analyses were grown from THF/pentane (**2-La** and **2-Sm**) or benzene/pentane (**2-Y** and **2-Yb**) solutions. The compounds crystallized in the space groups *P*21̅n (**2-La**) or *P*21̅c (**2-Sm**, **2-Y**, and **2-Yb**) and the analyses confirmed the dinuclear structure of the complexes with the three phosphanophenolate ligands bridging between the two metal centers by binding to the rare-earth metal center via the phenolate oxygen atoms and to the copper centers via the phosphanes (Figures 2 and S1 in the Supporting Information). The triflate group is bound to the rare-earth metal, resulting in an intramolecular charge separation with the rare-earth metal-containing fragment forming an *ate*-complex and the copper-containing substructure bearing one positive charge, forming an overall neutral complex. Among the otherwise isostructural dinuclear **2-Ln** complexes, we observe differences in the coordination sphere around the rare-earth center. In the case of the lanthanum derivative, the triflate group is bound to the lanthanum­(III) atom via a single oxygen atom, and the coordination sphere of the metal ion is saturated with one THF molecule, forming **2-La**·**THF**. The samarium­(III) derivative **2-Sm** exhibits triflate bound via two oxygen atoms without coordinated THF. The yttrium­(III) (**2-Y**) and ytterbium­(III) derivatives (**2-Yb**) show triflate bound via a single oxygen atom. This follows the decrease in the ionic radii from lanthanum­(III) to ytterbium­(III). We previously observed such behavior for similar rare-earth metal–silver complexes.^21^ The rare-earth metal–copper distances are in the range of 3.4591(6) (**2-La**·**THF**) to 3.2592(3) Å (**2-Yb**) and therefore just above the sums of the covalent radii of the metal atoms, ranging from 3.39 (La–Cu) to 3.19 Å (Yb–Cu).^24^ However, the copper atoms are slightly tilted out of plane formed by three coordinating phosphorus atoms and toward the rare-earth metal ions. The distances of the copper atoms to the calculated plane between the three phosphorus atoms are 0.142 (**2-La**), 0.159 (**2-Sm**), 0.138 (**2-Y**), and 0.135 Å (**2-Yb**), exhibiting a trend of decreasing distance from early rare-earth metal ions to the later ones. Considering our observations in similar rare-earth metal–silver complexes, we see no indication of dative metal–metal bonding but merely an electrostatic interaction between the negatively charged *ate*-fragment housing the rare-earth metal ion and the positively charged fragment containing the copper ion. The copper–phosphorus distances in the complexes cover similar ranges from 2.283(1) to 2.2920(6) Å, supporting that the situation is widely independent from that of the rare-earth metal center. The coordination geometries around the rare-earth metal ions vary. In the case of **2-La**·**THF**, a pseudotrigonal bipyramidal coordination is observed with the oxygen atom of the bound THF molecule (O4) and one phenolate oxygen atom at the apical positions (O3) and the triflate oxygen atom (O5) together with the remaining two phenolate oxygen atoms (O1 and O2) in the equatorial positions. The bidentate coordination of the triflate in **2-Sm** results in pseudosquare pyramidal geometry with the two triflate oxygen atoms together with the two phenolate oxygen atoms forming the base and the remaining phenolate oxo at the top of the pyramid. This contrasts the observations for the analogous rare-earth metal–silver complexes, where the triflate group in the lanthanum–silver complex exhibited bidentate coordination without THF coordination, despite being present in the crystallization solution, while the samarium–silver derivative showed the triflate bound to the samarium ion via a single oxygen atom along with coordination of a THF molecule. In the case of the rare-earth metal–copper complexes **2-Y** and **2-Yb**, we observe the triflate groups bound to the rare-earth metal ion by a single oxygen atom in distorted tetrahedral coordination geometries. The bond distances of the rare-earth metal ions to the phenolate oxygen atoms exhibit the expected decrease from the lanthanum derivative **2-La**·**THF**, 2.240(2) to 2.256(3) Å, to the ytterbium derivative **2-Yb**, 2.031(2) to 2.051(2) Å, following the decrease of the ionic radii of the rare-earth metal ions.^21^ The bond distances of the rare-earth metal ions to the triflate oxygen atoms are at 2.434(3) Å in **2-La**, which is shorter than in the case of the κ^2^-bound triflate in the similar lanthanum–silver complex. In the **2-Sm** complex, in which we encounter a bidentate coordination of the triflate to the samarium ion, the distances are 2.526(2) and 2.614(2) Å and therefore longer than in the similar samarium–silver complex, in which the triflate is bound to the samarium ion via a single oxygen atom. In the case of **2-Y** with 2.236(3) Å, the distance is slightly shorter than the corresponding one in the analogous yttrium–silver complex, whereas with 2.202(2) Å in **2-Yb**, it is slightly longer than in the similar ytterbium–silver complex (Table 1).

**Figure 2:**
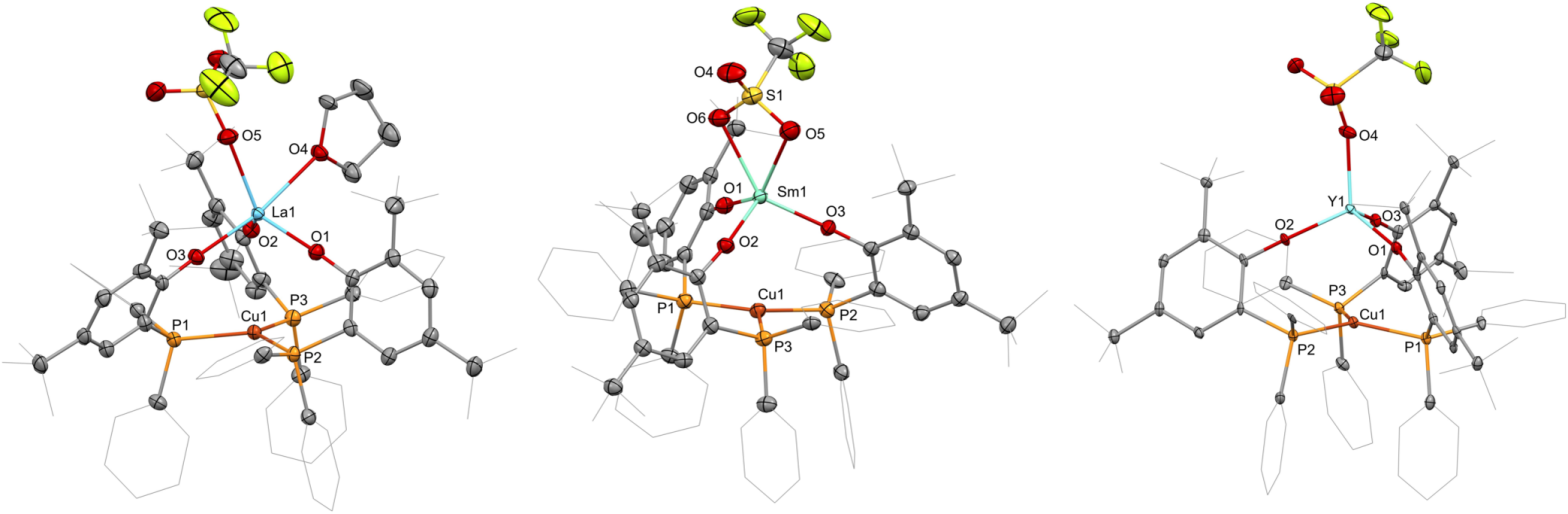
Molecular structure of **2-La**·**THF** (left), **2-Sm** (middle), and **2-Y** (right). Hydrogen atoms are omitted, and selected ligand carbon atoms are depicted as wireframe for clarity. Thermal ellipsoids are drawn at 50% probability. Selected distances (Å) and angles (°) are listed in Table 1.

**Table 1:** Selected Distances (Å) and Angles (°) from the Solid-State Structures of **2-La·THF**, **2-Sm**, **2-Y**, **2-Yb**, **4-La**, **4-Sm**, **4-Y**, and **4-Yb**

entry	**2-La**·**THF**	**2-Sm**	**2-Y**	**2-Yb**
Ln···Cu [Å]	3.4591(6)	3.3981(6)	3.3150(6)	3.2590(5)
Ln-O_Ar_ [Å]	2.240(2)	2.138(2)	2.069(2)	2.031(2)
	2.241(2)	2.143(2)	2.077(2)	2.051(2)
	2.256(3)	2.161(2)	2.084(2)	2.051(2)
Ln-O_Tf_ [Å]	2.434(3)	2.526(2)	2.236(3)	2.202(2)
Ln-O_Tf/THF_ [Å]	2.637(3)	2.614(2)		
P–Cu–P [°]	117.89(4)	115.97(3)	115.90(4)	115.35(3)
	118.78(4)	120.34(3)	120.63(4)	120.77(3)
	122.17(4)	122.24(3)	122.39(4)	122.84(3)

entry	**4-La**	**4-Sm**	**4-Y**	**4-Yb**
Ln···Cu [Å]	3.3028(9)	3.2882(8)	3.3059(9)	3.2848(8)
Ln-O_Ar_ [Å]	2.2898(10)	2.2018(11)	2.1250(8)	2.0917(9)
Ln-O_tBu_ [Å]	2.1430(17)	2.0735(18)	2.0261(14)	1.9991(17)
P–Cu–P [°]	119.898(1)	119.879(2)	119.859(1)	119.825(2)

The NMR spectroscopic data of the **2-Ln** (Ln = La, Sm, Y, Yb) exhibit one set of resonances for the three Ar^P^O^–^ ligands per complex. In ^1^H NMR spectroscopy, the diamagnetic complexes **2-La** and **2-Y** exhibited shifts in the range from 1.17 to 7.50 ppm and 1.17 to 7.47 ppm, respectively. The absence of resonances for THF in **2-La** suggests that THF is rather weakly bound to the lanthanum ion, which may be a result of the THF oxygen atom competing with a second triflate oxygen atom in coordination to the metal ion. The paramagnetic derivative **2-Sm** hardly exceeds the range spanning from 1.26 to 7.86 ppm. This is contrasted by the second paramagnetic derivative **2-Yb**, exhibiting the range from −25.15 to 27.63 ppm. The effective magnetic moments (μ_eff_) of **2-Sm** and **2-Yb**, determined by the Evans method are 1.1 and 3.6 μ_B_, respectively.^25^ For comparison, the corresponding rare-earth metal–silver complexes [Ln^III^(OTf)­(μ-OAr^P^-1κ^1^ *O*,2κ^1^ *P*)_3_Ag^I^], showed effective magnetic moments at 1.2 (Ln = Sm) and 4.7 μ_B_ (Ln = Yb) and the literature values for the ions were reported at 0.85 (Sm^III^) and 4.53 μ_B_ (Yb^III^), respectively.^21,26^ In the case of the ^13^C NMR data of **2-Y**, we can observe the diphenylphosphane-bound phenolate *ipso*-carbon atom as a doublet of doublets of doublets (Figure 3). The resonance is composed of a ^1^ *J* coupling to the directly bound phosphorus atom with 32 Hz, along with two ^3^ *J* couplings of 17 and 3 Hz magnitude. Considering that spin–spin couplings are sensitive to relative orientations of the nuclei involved, we adopt the Michl–West nomenclature to describe the dihedral angles, as it enables a more nuanced assignment in comparison with the Prelog–Klyne nomenclature.^27^ Using the solid-state structure of **2-Y** as a basis for the assignment, we assign the ^3^ *J* coupling of 17 Hz to the ^31^P nucleus with the *deviant* conformation at 155° and the ^3^ *J* coupling of 3 Hz to ^31^P nucleus with the *cisoid* conformation of 33°.^28^ The ^31^P NMR shifts of the diamagnetic complexes are −6.2 (**2-La**) and −6.8 ppm (**2-Y**). In **2-Sm**, the paramagnetic samarium ion appears to have only a limited influence on the ^31^P NMR resonance, which is observed at −7.2 ppm. This contrasts with the situation for the second paramagnetic ytterbium complex **2-Yb**, as it exhibits the corresponding resonance at 51.8 ppm. For comparison, the corresponding ^31^P NMR signal for the free ligand is observed at −30.8 ppm.^29^

**Figure 3:**
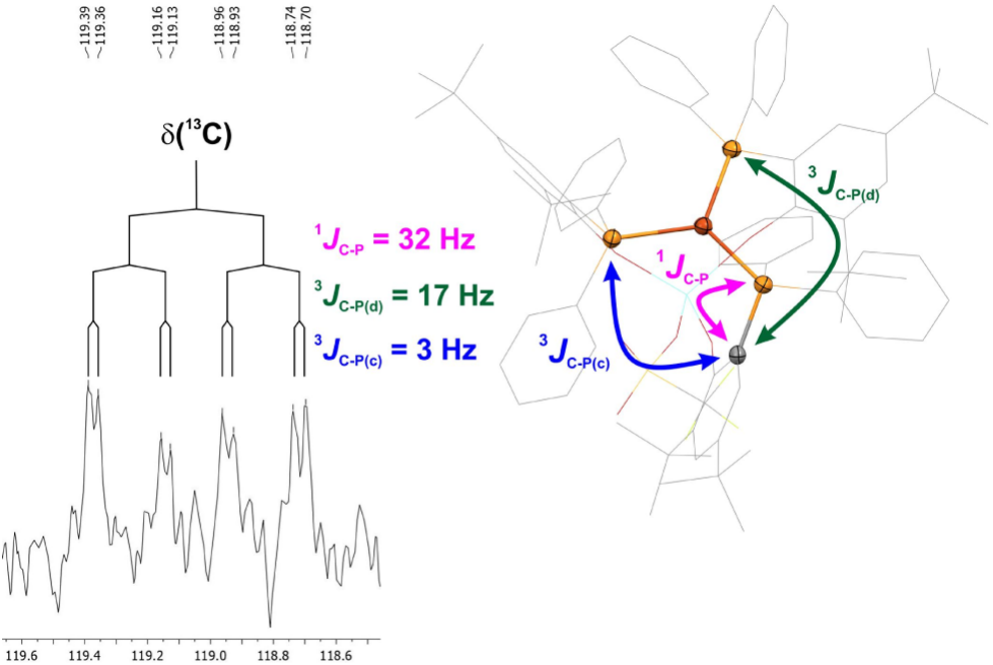
^13^C­{^1^H} NMR resonance of the phosphane-substituted *ipso*-C atom of the phenolate substructure in **2-Y**, which exhibits different couplings to all three ^31^P nuclei coordinated to the copper­(I) atom.

As part of this research, we also explored the possibility of preparing the heterobimetallic **2-Ln** complexes starting from the corresponding monometallic copper compound [(HOAr^P^)_3_Cu^I^OTf] **3** as an alternative synthetic pathway. We prepared complex **3** by reaction of copper­(I) triflate toluene adduct and HOAr^P^ in a 1:3 molar ratio in toluene at ambient temperature, yielding the colorless compound in 94% (Scheme 2). Initial attempts to use a copper­(I) bromide dimethylsulfide adduct or copper­(I) chloride resulted in the undesired loss of hydrogen bromide or hydrogen chloride, respectively. Therefore, we moved to the corresponding copper­(I) triflate starting materials. Also, any attempts to prepare complexes with a copper­(I) to HOAr^P^ ratio below 1:3 using copper­(I) triflate toluene adduct or bis­(cyclooctadiene)­copper­(I) triflate only yielded **3**. The single-crystal X-ray diffraction analysis shows **3** to crystallize in the monoclinic space group *P*21̅*c* and the copper atom in trigonal planar coordination by the three phosphane moieties of the HOAr^P^ ligands, with the triflate group not bonded to the metal ion (Figure 4). The solid-state structure further shows the phosphane-bound phenolate groups above and below the coordination plane of the cooper atom. Considering the steric demand of the substructure in comparison to the two phenyl groups, the rotation of the ligand around the Cu–P axis may be slow enough that the reaction of **3** with [Ln­[N­(SiMe_3_)_2_]_3_] might include side-reactions due to remaining hydroxyl or rare-earth metal amides being available for conversion with one or more additional reactant molecules, causing the formation of higher oligomers. The copper–phosphorus distances are in the range of 2.2676(7)–2.2786(6) Å and thus shorter than the corresponding bonds in the heterobimetallic **2-Ln** complexes. The three P–Cu–P angles sum up to 359.94°, underlining the planar coordination geometry. The NMR spectroscopic data exhibit only a single set of resonances for the three HOAr^P^ ligands, and the broadened ^31^P NMR signal is observed at −11.6 ppm with a full width at half-maximum of 151 Hz. Treatment of **3** with [Ln­[N­(SiMe_3_)_2_]_3_] (Ln = La, Y) using toluene or THF at ambient or temperatures of up to 60 °C gave only **2-La** or **2-Y**, respectively, along with additional products, which were removed by multiple washings with pentane. This is similar to what we previously observed for the analogous rare-earth metal–silver complexes.^21^ Since this pathway was not a viable alternative to the initially used synthesis, we did not utilize this alternative approach any further.

**Figure 4:**
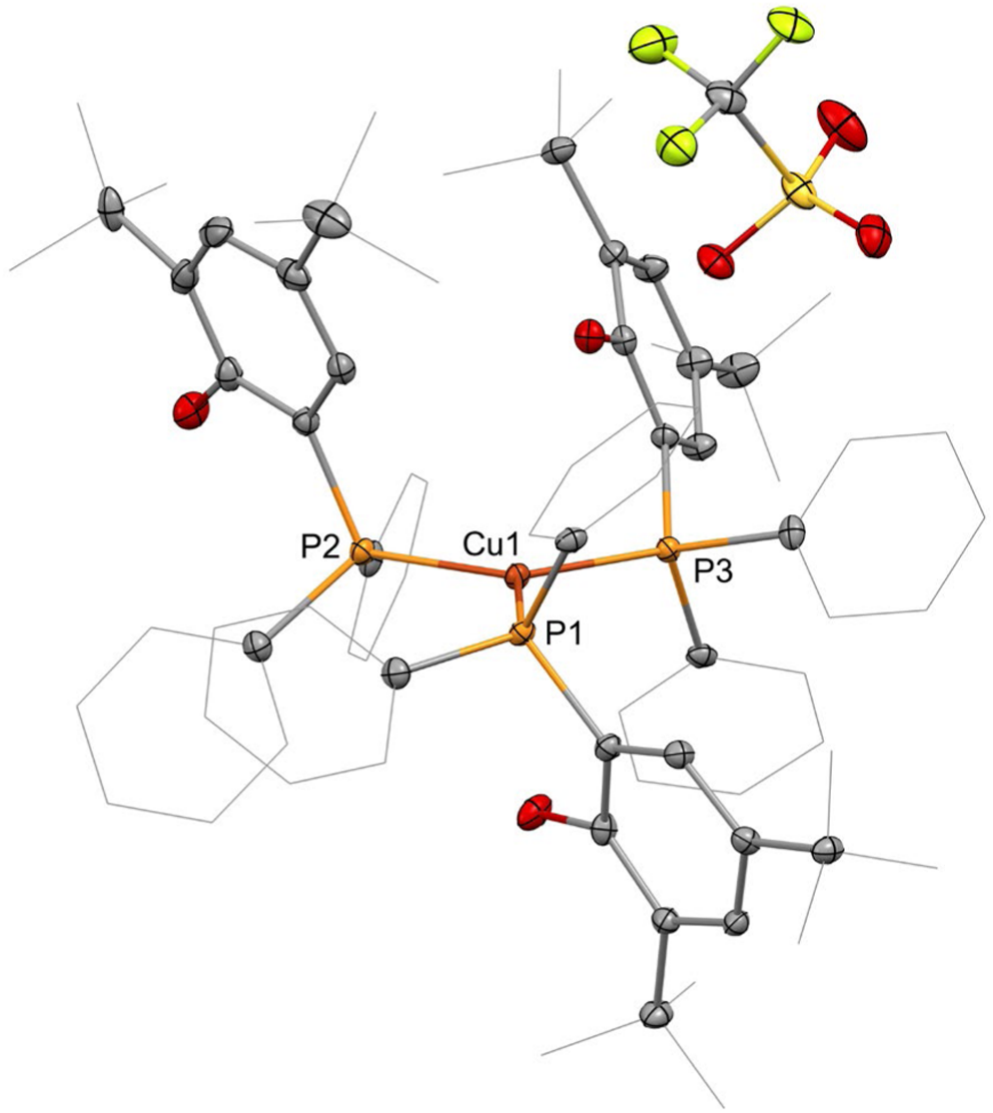
Molecular structure of **3**. Hydrogen atoms are omitted, and selected ligand carbon atoms are depicted as a wireframe for clarity. Thermal ellipsoids drawn at 50% probability. Selected distances (Å) and angles (°): Cu1–P1:2.2697(7), Cu1–P2:2.2786(6), Cu1–P3:2.2676(7), P1–Cu1–P2:121.77(3), P2–Cu1–P3:117.23(3), and P1–Cu1–P3:120.94(3).

**Scheme 2:**
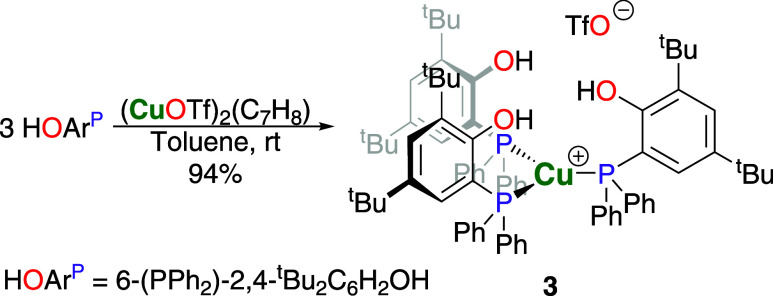
Synthesis of the Monometallic Copper­(I) Complex (HOAr^P^)_3_Cu^I^OTf **3**

Considering that there is a long-standing interest in cationic late transition metal catalysts in the context of metal–substrate interaction, we were interested in investigating the stability of the dinuclear complexes, in which the positive charge is compensated intramolecularly by an *ate*-complex fragment, toward ligand exchange reactions on the latter. Accordingly, we explored the exchange of the triflate group in **2-Ln** for amido or alkyl groups, aiming to prepare precatalysts for hydrofunctionalization catalysis. However, the equimolar treatment of the **2-Ln** compounds with sodium hexamethyldisilazide, lithium diethylamide, or lithium *iso*-butylamide resulted in decomposition into monometallic species such as copper­(I) hexamethyldisilazide tetramer (see Supporting Information for experimental details for selected derivatization attempts). The reactions of **2-Ln** with equimolar amounts of methyllithium or benzylpotassium resulted in decomposition under the precipitation of dark gray to black powder. As it was unclear whether the stability of the complexes was limited to triflate complexes, we treated **2-Ln** complexes with equimolar amounts of potassium *tert*-butanolate in THF at ambient temperature. This resulted in the clean exchange of the triflate for *tert*-butanolate groups in **2-Ln** yielding [Ln^III^(O^t^Bu)­(μ-OAr^P^-1κ^1^ *O*,2κ^1^ *P*)_3_Cu^I^] (**4-Ln**, Ln = La, Sm, Y, Yb) in 49–61% after crystallization from benzene/pentane (Scheme 3). This underlines the possibility of rare-earth metal-centered ligand exchange under preservation of the dinuclear structure of the complexes, which is an essential aspect in catalytic processes using complexes of similar dinuclear structures. The heterometallic complexes **4-Ln** are colorless, which is somewhat unexpected for the samarium (**4-Sm**) and especially the ytterbium derivatives (**4-Yb**), as the precursors **2-Sm** and **2-Yb** are colored and in the case of the latter intensely so. The single-crystal X-ray diffraction analyses show that the four **4-Ln** (Ln = La, Sm, Y, Yb) complexes are isostructural and crystallize in the space group *R̅*3 (Figures 5 and S3–S5 in the Supporting Information). The intermetallic distances are 3.3028(9) Å (**4-La**), 3.2882(8) Å (**4-Sm**), 3.3059(9) Å (**4-Y**), and 3.2848(8) Å (**4-Yb**). Therefore, they do not exhibit a trend following the decrease of the ionic radii, as is observed for the parent **2-Ln** complexes, which may be attributed to packing effects in the solid state. With the exception of **4-Sm**, the intermetallic distances are just above the sums of the covalent radii of the respective metal ions.^24^ We attribute the comparably close proximity of the two metal ions also for the **4-Ln** complexes to predominantly be the result of the ligands bridging the metal ions in combination with the electrostatic interaction of the charges distributed across the rare-earth metal- and copper-containing substructures of the complexes. Also, in the case of the **4-Ln** complexes, a tilt of the copper atoms toward the rare-earth metal atoms is observed. However, the extent is smaller than in the parent **2-Ln** compounds but exhibits a trend of increasing distance from lanthanum to ytterbium. Here, the distances of the copper atoms from the calculated plane between the three phosphorus atoms are 0.073 (**4-La**), 0.079 (**4-Sm**), 0.086 (**4-Y**), and 0.096 Å (**4-Yb**). The rare-earth-metal-bound *tert*-butanolates are coordinated linearly, with Ln-O-CMe_3_ angles at 180.0° for all **4-Ln** complexes, with both oxygen and tertiary carbon atom located on the metal–metal axis. The distances of the rare-earth metal ions to the phenolate oxygen atoms range from 2.0917(9) Å (**4-Yb**) to 2.2898(10) Å (**4-La**) and those to the *tert*-butanolate oxygen atoms range from 1.9991(17) Å (**4-Yb**) to 2.1430(17) Å (**4-La**). The copper–phosphorus distances decrease from 2.2769(3) Å in **4-La** to 2.2686(3) Å in **4-Yb** and are slightly shorter than those in **3** as well as in the corresponding **2-Ln** complexes.

**Figure 5:**
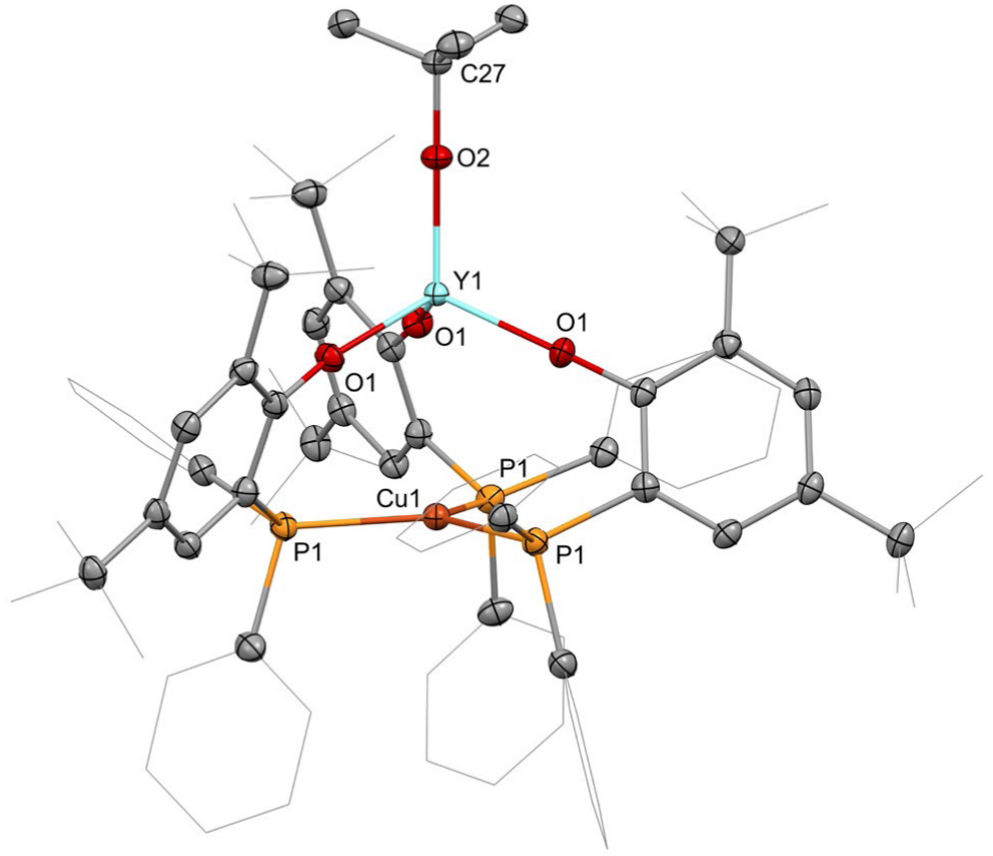
Molecular structure of **4-Y**. Hydrogen atoms are omitted, and selected ligand carbon atoms are depicted as a wireframe for clarity. Thermal ellipsoids drawn at 50% probability. Selected distances (Å) and angles (deg): Y1···Cu1:3.3059(9), Y1–O1:2.1250(8), Y1–O2:2.0261(14), Cu1–P1:2.2711(3), O1–Y1–O2:110.67(2), Y1–O2–C27:180.00, and P1–Cu1–P1′: 119.859(1).

**Scheme 3:**
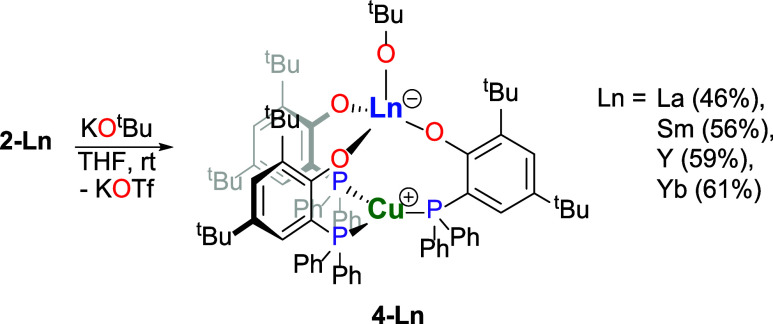
Synthesis of the Phosphanophenolate-Bridged Rare-Earth Metal­(III)–Copper­(I) *tert*-Butanolate Complexes (**4-Ln**, Ln = La, Sm, Y, Yb)

The NMR spectroscopic analysis of the **4-Ln** complexes showed a picture similar to that of parent heterometallic complexes **2-Ln**. The ^1^H NMR resonances of the diamagnetic compounds are observed in ranges from 1.21 to 7.45 ppm (**4-La**) and from 1.23 to 7.45 ppm (**4-Y**). In the case of the paramagnetic complexes, the ranges are from −0.15 to 8.21 ppm (**4-Sm**) and −54.66 to 33.24 ppm (**4-Yb**). The ^31^P NMR resonances of **4-La** (−5.9 ppm), **4-Sm** (−3.6 ppm), and **4-Y** (−7.2 ppm) appear similar to those in the corresponding precursor, whereas in the case of **4-Yb**, the signal shifted to 51.8 ppm, which is a difference of more than 100 ppm. The Evans method measurements showed the effective paramagnetic moments also very similar to the precursors at 1.1 (**4-Sm**) and 3.8 μ_B_ (**4-Yb**), respectively.^25^

### Optical Spectroscopy

The UV–vis data were recorded from solutions in toluene and show the spectra dominated by ligand-related π–π* transition absorptions of the aromatic substructures (see Figures S32–S41 in the Supporting Information). The absorptions observed for the complexes **2-Ln** rise to the cutoff wavelength of 310 nm to molar extinction coefficients in the range from 1.2 × 10^4^ (**2-Sm**) to 1.5 × 10^4^ (**2-La**) L mol^–1^cm^–1^. A weak shoulder in the spectrum of **2-Sm** at 381 nm (ε = 3.2 × 10^2^ L mol^–1^cm^–1^) may be assigned to the f–f transition from the ground-state ^6^H_5/2_ to ^4^D_1/2_.^30^ The observed absorption of the mononuclear complex **3** rising to 2.6 × 10^3^ L mol^–1^cm^–1^, still appearing as a steep slope at the cutoff wavelength, might indicate a bathochromic shift of the absorptions relating to π–π* transitions upon deprotonation of the phenol group as part of the formal incorporation of the rare-earth metal ion into the complexes. The broad, weak absorption in the spectrum of **2-Yb** at 409 nm (3.2 × 10^2^ L mol^–1^cm^–1^) may be attributed to a ligand-to-metal charge transfer process.^31,32^ For the **4-Ln** complexes, the situation is similar, exhibiting the highest absorptions at the cutoff wavelength of 310 nm with extinction coefficients ranging from 1.8 × 10^4^ (**4-La**, **4-Sm**) to 2.2 × 10^4^ L mol^–1^cm^–1^ (**4-Y**). Additional features in the UV–vis spectra of these compounds were observed as shoulders in cases of **4-La** at 347 nm (2.4 × 10^3^ L mol^–1^cm^–1^) and **4-Yb** at 347 nm (7.4 × 10^3^ L mol^–1^cm^–1^), which may also be related to π–π* transitions.

The IR spectra of **2-Ln** complexes (Ln = La, Sm, Y, Yb) exhibit the resonances characteristic of the deformation vibrations, δ_s_ and δ_as_, of *tert*-butyl groups at 1421–1432 cm^–1^ (see Figures S42–S50 in the Supporting Information).^33^ In the spectra of the corresponding **4-Ln** compounds, the additional *tert*-butyl group results in a stronger absorption, which also merges the two bands into a single one in the range from 1424 to 1427 cm^–1^. The loss of the band in the range from 1179 to 1186 cm^–1^ in **2-Ln** upon exchange of the triflate group for the *tert*-butyl group indicates a relation to the former. Considering previous IR studies of triflate complexes, the bands may be assigned to the asymmetric bond stretching vibration, ν_as_, of the CF_3_ group of the triflate.^34^ However, the presence of other bands in this area of the spectra, along with overlaps, does not allow an unambiguous assignment.

## Conclusions

In conclusion, we presented the synthesis and characterization of the heterodinuclear rare-earth metal­(III)–copper­(I) complexes [Ln^III^(OTf)­(μ-OAr^P^-1κ^1^ *O*,2κ^1^ *P*)_3_Cu^I^] (**2-Ln**, Ln = La, Sm, Y, Yb, Ar^P^O^–^ = 2,4-*^t^* Bu_2_-6-(Ph_2_P)­C_6_H_2_O^–^) by reaction of the corresponding mononuclear rare-earth metal complexes [Ln^III^(OAr^P^-κ^2^ *O*,*P*)_3_] (**1-Ln**, Ln = La, Sm, Y, Yb) with [(Cu^I^OTf)_2_(C_7_H_8_)] in equimolar ratios. In order to explore the alternative synthetic pathway toward the heterodinuclear complexes starting from a mononuclear copper complex, [(HOAr^P^-κ*P*)_3_Cu^I^] (**3**) was prepared by reaction of [(Cu^I^OTf)_2_(C_7_H_8_)] with three equivalents of HOAr^P^. Further treatment of **3** with [Ln^III^[N­(SiMe_3_)_2_]_3_] (**1-Ln**, Ln = La, Y) was found to give the corresponding heterodinuclear complexes **2-Ln** only in low yields. Therefore, this pathway was not further utilized. In the context of catalyst development, we were also interested in rare-earth metal–copper complexes featuring terminal ligands on the rare-earth metal center different from the triflate. The exchange of the triflate group in **2-Ln** for amide, alkyl, or alkoxy groups under preservation of the dinuclear structure was only successful for the latter and performed by reaction of **2-Ln** with equimolar amounts of KO^t^Bu yielding [Ln^III^(O^t^Bu)­(μ-OAr^P^-1κ^1^ *O*,2κ^1^ *P*)_3_Cu^I^] (**4-Ln**, Ln = La, Sm, Y, Yb). The intermetallic distances of the complexes are typically just above the sums of the covalent radii of the metal ions involved. However, we do not see an indication that the copper ions act as donors to the rare-earth metal ions, since the ^31^P NMR shifts of mononuclear copper complex **3** do not exhibit extensive differences from those of the diamagnetic rare-earth metal complexes. Considering the intramolecular charge separation, we see the metal–metal interaction to be dominated by the electrostatic interaction of the positive charge of the copper-containing fragment and the negative charge of the rare-earth metal fragment.

## Experimental Section

### General Details

All manipulations were carried out under an atmosphere of dry, oxygen-free nitrogen using standard Schlenk and glovebox techniques. Benzene-*d*_6_ was distilled from potassium. THF was purified by distillation from calcium hydride under nitrogen. All other solvents were purified by passing through columns of activated alumina.^35^ [Ln^III^(OAr^P^-κ^2^ *O*,*P*)_3_] (Ln = La, Sm, Y, Yb),^21^ Ln^III^[N­(SiMe_3_)_2_]_3_ (Ln = La, Sm, Yb, Y),^36^ [(C_7_H_8_)­(Cu^I^OTf)_2_],^37^ and 2,4-^t^Bu_2_-6-(PPh_2_)­PhOH^29^ were prepared according to published procedures. Other chemicals were obtained from different suppliers and used without further purification. The NMR spectra were recorded on a JEOL JNM-ECZL 400 MHz or a Bruker AVANCE III 300 instrument and are referenced to Me_4_Si (^1^H, ^13^C). The ^1^H NMR data required for Evans NMR method calculations of the effective magnetic moments, μ_eff_, were recorded by using solutions of the analytes in benzene-*d*_6_ with and without a sealed capillary containing benzene-*d*_6_ to determine the shift differences between the solvent residue peaks. The shift differences of the resonances were then used for the calculations.^25,38^ For X-ray structure analyses, the crystals were mounted onto the tips of glass fibers or nylon loops. Data collection was performed with a Bruker-AXS SMART APEX CCD diffractometer using graphite-monochromated Mo Kα radiation (0.71073 Å), a Rigaku XtaLAB Synergy, Dualflex, HyPix-Arc 100 diffractometer, or a Bruker APEX II diffractometer using an Incoatec microfocus sealed tube of Mo Kα radiation (0.71073 Å) with a CCD area detector. The data were reduced to F_o_^2^ and corrected for absorption effects with SAINT^39^ and SADABS^40,41^ or CrysAlisPro,^42^ respectively. The structures were solved by direct methods and refined by the full-matrix least-squares method (SHELXL97 or SHELXL19).^43^ If not noted otherwise, all non-hydrogen atoms were refined with anisotropic displacement parameters. All hydrogen atoms were located in calculated positions to correspond to the standard bond lengths and angles. UV–vis spectra were recorded on an Agilent Cary 60 UV–vis spectrophotometer using toluene to prepare the analyte solutions for the measurements. Elemental analysis was carried out using a Heraeus VARIO ELEMENTAR instrument and could only be performed for compounds free of fluorine. IR data were recorded on a Bruker α-T ATR-FTIR spectrometer.

**Caution!** *Extreme care should be taken in both the handling of the cryogen liquid nitrogen and its use in the Schlenk line trap to avoid the condensation of oxygen from air. Benzene as well as benzene-d*_6_ *are carcinogenic and must be handled to avoid any contact with the human body and disposed according to local safety regulations. All novel compounds have not been tested regarding their toxicity and should be regarded as toxic chemicals and handled, avoiding any contact with the human body, and disposed according to local safety regulations. Methyllithium and benzylpotassium are pyrophoric materials and should be handled with great care and disposed according to local safety regulations*.

### Syntheses

#### Lanthanum­(III)­tris­[2,4-di-*tert*-butyl-6-(diphenylphosphan-yl)­phenolate]­copper­(I) Triflate [La^III^(OTf)­(OAr^P^-1κ^1^ *O*,2κ^1^ *P*)_3_Cu^I^] **2-La**

A vial equipped with a magnetic stir bar was charged with **1-La** (654 mg, 500 μmol), [(Cu^I^OTf)_2_(C_7_H_8_)] (137 mg, 265 μmol), and toluene (10 mL) and stirred at ambient temperature for 18 h. The solution was then filtered using a syringe filter. After that, all volatiles were evaporated under reduced pressure, the residue was dissolved in minimal amounts of benzene at 65 °C, and after cooling to ambient temperature, the resulting solution was layered with pentane to afford crystallization. The crystalline material was isolated, washed three times with pentane, and dried under vacuum, yielding **2-La** as a colorless crystalline solid (249 mg, 33%). Mp 197 °C (dec.). ^1^H NMR (δ in ppm, benzene-*d*_6_, 298 K): 1.17 (s, 27H, ^*t*^ *Bu*), 1.36 (s, 27H, ^*t*^ *Bu*), 6.63 (dt, ^3^ *J*_(31)P,(1)H_ = 6 Hz, ^4^ *J*_(1)H,(1)H_ = 3 Hz, Ar*H*), 6.67–6.77 (m, 6H, Ar*HH*), 6.78–6.91 (m, 9H, Ar*H*H*H*), 6.99 (br s, 3H, Ar*H*), 7.09 (br s, 6H, Ar*HH*), 7.16 (s, 3H, Ar*H*), 7.42 (d, ^4^ *J*_(1)H,(1)H_ = 3 Hz, Ar*H*), 7.34–7.50 (br s, 6H, Ar*H*). ^13^C­{^1^H} NMR (δ in ppm, benzene-*d*_6_, 298 K): 29.5 (^*t*^ *Bu*), 31.6 (^*t*^ *Bu*), 34.2 (^*t*^ *Bu*), 126.2 (*C*_*Ar*_), 126.9 (*C*_*Ar*_), 128.3 (*C*_*Ar*_), 128.7 (*C*_*Ar*_), 129.8 (*C*_*Ar*_), 132.2 (*C*_*Ar*_), 135.5 (*C*_*Ar*_), 137.6 (*C*_*Ar*_). ^31^P­{^1^H} NMR (δ in ppm, benzene-*d*_6_, 298 K): −6.2. UV–vis (λ nm, ε L mol^–1^cm^–1^ in brackets): 310 (1.5 × 10^4^). IR (ATR, cm^–1^): 428, 446, 465, 512, 578, 639, 673, 693, 743, 772, 836, 883, 915, 1020, 1090, 1114, 1178, 1200, 1230, 1253, 1284, 1325, 1360, 1421, 1460, 1478, 1587, 1625, 2865, 2958, 3057. CCDC: 2381853.

#### Samarium­(III)­tris­[2,4-di-*tert*-butyl-6-(diphenylphosphanyl)­phenolate]­copper­(I) Triflate [Sm^III^(OTf)­(OAr^P^-1κ^1^ *O*,2κ^1^ *P*)_3_Cu^I^] **2-Sm**

The complex was prepared via the same procedure that was employed for **2-La** using **1-Sm** (660 mg, 500 μmol) and [(Cu^I^OTf)_2_(C_7_H_8_)] (139 mg, 269 μmol), yielding **2-Sm** as a pale yellow microcrystalline solid (362 mg, 47%). Mp 188 °C (dec.). ^1^H NMR (δ in ppm, benzene-*d*_6_, 298 K): 1.26 (s, 27H, ^*t*^ *Bu*), 2.40 (s, 27H, ^*t*^ *Bu*), 6.37 (dt, ^3^ *J*_(31)P,(1)H_ = 6 Hz, ^4^ *J*_(1)H,(1)H_ = 3 Hz, 3H, Ar*H*), 6.47 (br s, 6H, Ar*HH*), 6.60 (br s, 6H, Ar*HH*), 6.71 (br s, 3H, Ar*HH*), 7.02 (br s, 6H, Ar*HH*), 7.12 (br s, 3H, Ar*HH*), 7.16 (s, 3H, Ar*H*), 7.86 (d, ^4^ *J*_(1)H,(1)H_ = 3 Hz, 3H, Ar*H*). ^13^C­{^1^H} NMR (δ in ppm, benzene-*d*_6_, 298 K): 31.3 (^*t*^ *Bu*), 31.8 (^*t*^ *Bu*), 34.8 (^*t*^ *Bu*), 36.3 (^*t*^ *Bu*), 126.9 (*C*_*Ar*_). ^31^P­{^1^H} NMR (δ in ppm, benzene-*d*_6_, 298 K): −7.2. μ_eff_ = 1.1 μ_B_. UV–vis (λ nm, ε L mol^–1^cm^–1^ in brackets): 310 (1.2 × 10^4^), 381 (3.2 × 10^2^, sh). IR (ATR, cm^–1^): 429, 465, 512, 578, 639, 673, 693, 743, 770, 838, 883, 917, 1023, 1091, 1114, 1182, 1205, 1229, 1254, 1284, 1325, 1360, 1421, 1460, 1477, 1628, 2864, 2901, 2949, 3057. CCDC: 2347686.

#### Yttrium­(III)­tris­[2,4-di-*tert*-butyl-6-(diphenylphosphanyl)­phenolate]­silver­(I) Triflate [Y^III^(OTf)­(OAr^P^-1κ^1^ *O*,2κ^1^ *P*)_3_Cu^I^] **2-Y**

The complex was prepared via the same procedure that was employed for **2-La** using **1-Y** (252 mg, 200 μmol) and [(Cu^I^OTf)_2_(C_7_H_8_)] (52 mg, 100 μmol). The complex was isolated as colorless crystals of **2-Y** (125 mg, 43%). Mp 199 °C (dec.). ^1^H NMR (δ in ppm, benzene-*d*_6_, 298 K): 1.17 (s, 27H, ^*t*^ *Bu*), 1.41 (s, 27H, ^*t*^ *Bu*), 6.66 (dt, ^3^ *J*_(31)P,(1)H_ = 6 Hz, ^4^ *J*_(1)H,(1)H_ = 3 Hz, 3H, Ar*H*), 6.69 (d, ^3^ *J*_(1)H,(1)H_ = 7 Hz, Ar*H*), 6.79 (d, ^3^ *J*_(1)H,(1)H_ = 7 Hz, Ar*H*), 6.849 (t, ^3^ *J*_(1)H,(1)H_ = 7 Hz, Ar*H*), 6.97–7.06 (m, 9H, Ar*HH*), 7.16 (s, 3H, Ar*H*), 7.39 (d, ^4^ *J*_(1)H,(1)H_ = 3 Hz, 3H, Ar*H*), 7.47 (br s, 6H, Ar*HH*). ^13^C­{^1^H} NMR (δ in ppm, benzene-*d*_6_, 298 K): 29.9 (^*t*^ *Bu*), 31.9 (^*t*^ *Bu*), 34.5 (^*t*^ *Bu*), 119.0 (ddd, ^1^ *J*_(31)P,(13)C_ = 32 Hz, ^3^ *J*_(31)P,(13)C_ = 17 Hz, ^3^ *J*_(31)P,(13)C_ = 3 Hz, P*C*_*Ar*_), 126.5 (*C*_*Ar*_), 127.2 (*C*_*Ar*_), 129.1 (*C*_*Ar*_), 130.4 (*C*_*Ar*_), 132.4 (*C*_*Ar*_), 134.5 (*C*_*Ar*_), 135.7 (*C*_*Ar*_), 138.6 (*C*_*Ar*_), 140.1 (*C*_*Ar*_), 163.1 (*C*_*Ar*_). ^31^P­{^1^H} NMR (δ ppm, benzene-*d*_6_): −6.8. UV–vis (λ nm, ε L mol^–1^cm^–1^ in brackets): 310 (1.5 × 10^4^). IR (ATR, cm^–1^): 435, 465, 499, 513, 533, 578, 638, 693, 743, 771, 840, 883, 917, 1022, 1091, 1114, 1183, 1203, 1230, 1256, 1285, 1329, 1360, 1389, 1421, 1460, 1477, 1587, 1628, 2864, 2902, 2949, 3054. CCDC: 2381852.

#### Ytterbium­(III)­tris­[2,4-di-*tert*-butyl-6-(diphenylphosphanyl)­phenolate]­copper­(I) Triflate [Yb^III^(OTf)­(OAr^P^-1κ^1^ *O*,2κ^1^ *P*)_3_Cu^I^] **2-Yb**

The complex was prepared via the same procedure that was employed for **2-La** using **1-Yb** (537 mg, 400 μmol) and [(Cu^I^OTf)_2_(C_7_H_8_)] (104 mg, 200 μmol). The complex was isolated as amber-colored crystals of **2-Yb** (551 mg, 89%). Mp 206 °C (dec.). ^1^H NMR (δ in ppm, benzene-*d*_6_, 298 K): −25.15 (s, 27H, ^*t*^ *Bu*), −6.39 (s, 3H, Ar*H*), −2.13 (s, 6H, Ar*H*), −1.61 (s, 27H, ^*t*^ *Bu*), −1.34 (s, 3H, Ar*H*), 2.72 (br s, 3H, Ar*H*), 11.57 (s, 3H, Ar*H*), 13.63 (s, 3H, Ar*H*), 15.37 (s, 6H, Ar*H*), 27.63 (br s, 6H, Ar*H*). ^31^P­{^1^H} NMR (δ in ppm, benzene-*d6*, 298 K): 51.8. μ_eff_ = 3.6 μ_B_. UV–vis (λ nm, ε L mol^–1^cm^–1^ in brackets): 310 (1.4 × 10^4^), 409 (3.2 × 10^2^). IR (ATR, cm^–1^): 432, 448, 465, 499, 513, 534, 580, 635, 673, 693, 741, 771, 843, 883, 917, 1012, 1091, 1115, 1199, 1230, 1256, 1287, 1329, 1345, 1359, 1424, 1460, 1477, 1628, 2863, 2949, 3057. CCDC: 2381887.

#### Tris­[2,4-di*tert*-butyl-6-(diphenylphosphanyl)­phenol]­copper­(I) Triflate [(HOAr^P^-κ*P*)_3_Cu^I^OTf] **3**

A Schlenk flask equipped with a magnetic stir bar was charged with [(Cu^I^OTf)_2_(C_7_H_8_)] (518 mg, 1.00 mmol), HOAr^P^ (2.34 g, 6.00 mmol), and toluene (20 mL) and stirred for 18 h. Then, the solvent was concentrated under reduced pressure to a volume of ca. 5 mL, and pentane (40 mL) was added to afford precipitation of the product. The precipitate was separated from the supernatant solution, washed with pentane, and dried under vacuum, affording a colorless solid (2.59 g, 94%). Mp 144 °C. ^1^H NMR (δ in ppm, benzene-*d*_6_, 298 K): 1.12 (s, 27H, ^*t*^ *Bu*), 1.43 (s, 27H, ^*t*^ *Bu*), 6.90–7.05 (m, 21H, Ar*H*), 7.31 (s, 3H, O*H*), 7.48 (s, 12H, Ar*H*), 7.59 (d, 3H, ^4^ *J*_(1)H(1)H_ = 2 Hz, Ar*H*). ^13^C­{^1^H} NMR (δ in ppm, benzene-*d*_6_, 298 K): 30.3 (^*t*^ *Bu*), 31.4 (^*t*^ *Bu*), 34.7 (^*t*^ *Bu*), 35.3 (^*t*^ *Bu*), 121.4 (*C*_*Ar*_), 127.6 (*C*_*Ar*_), 129.1 (*C*_*Ar*_), 129.1 (*C*_*Ar*_), 130.5 (*C*_*Ar*_), 131.5 (*C*_*Ar*_), 134.0 (*C*_*Ar*_), 140.1 (*C*_*Ar*_), 144.9 (*C*_*Ar*_), 155.6 (*C*_*Ar*_). ^31^P­{^1^H} NMR (δ ppm, benzene-*d*_6_): −11.6. UV–vis (λ nm, ε L mol^–1^cm^–1^ in brackets): 310 (2.6 × 10^3^). IR (ATR, cm^–1^): 411, 514, 571, 629, 663, 734, 764, 825, 852, 872, 908, 989, 1026, 1084, 1142, 1162, 1197, 1223, 1243, 1268, 1322, 1352, 1433, 1454, 1487, 1593, 2841, 2882, 2949, 3013, 3028. CCDC: 2347685.

#### Lanthanum­(III)­tris­[2,4-di-*tert*-butyl-6-(diphenylphosphanyl)­phenolate]­copper­(I) *tert*-Butanolate [La^III^(O^t^Bu)­(OAr^P^-1κ^1^ *O*,2κ^1^ *P*)_3_Cu^I^] **4-La**

A vial equipped with a magnetic stir bar was charged with **2-La** (304 mg, 200 μmol), potassium *tert*-butanolate (23 mg, 205 μmol), and THF (5 mL), and the solution was stirred at ambient temperature for 18 h. Then, the solvent was evaporated, the colorless residue was taken up with benzene, and the resulting solution was filtered using a syringe filter. The solution was concentrated under reduced pressure, and the addition of pentane afforded crystallization. The crystalline material was isolated, washed three times with pentane, and dried under vacuum, yielding **4-La** as a colorless crystalline solid (134 mg, 46%). Mp 180 °C (dec.). ^1^H NMR (δ in ppm, benzene-*d*_6_, 298 K): 1.21 (s, 27H, ^*t*^ *Bu*), 1.47 (s, 27H, ^*t*^ *Bu*), 1.54 (s, 9H, ^*t*^ *Bu*), 6.61 (dt, ^3^ *J*_(31)P,(1)H_ = 6 Hz, ^4^ *J*_(1)H,(1)H_ = 3 Hz, 3H, Ar*H*), 6.70–6.93 (m, 15H, Ar*HH*), 6.95–7.07 (m, 6H, Ar*H*H*H*), 7.19 (br s, 3H, Ar*H*), 7.45 (d, ^4^ *J*_(1)H,(1)H_ = 3 Hz, 9H, Ar*HH*). ^13^C­{^1^H} NMR (δ in ppm, benzene-*d*_6_, 298 K): 29.8 (^*t*^ *Bu*), 31.7 (^*t*^ *Bu*), 34.1 (^*t*^ *Bu*), 34.2 (^*t*^ *Bu*), 34.5 (^*t*^ *Bu*), 126.0 (*C*_*Ar*_), 126.4 (*C*_*Ar*_), 128.6 (*C*_*Ar*_), 132.3 (*C*_*Ar*_), 135.4 (*C*_*Ar*_), 137.1 (*C*_*Ar*_). ^31^P­{^1^H} NMR (δ in ppm, benzene-*d*_6_, 298 K): −5.9. UV–vis (λ nm, ε L mol^–1^cm^–1^ in brackets): 315 (1.8 × 10^4^), 358 (2.4 × 10^3^, sh). IR (ATR, cm^–1^): 425, 448, 465, 497, 510, 578, 628, 690, 743, 775, 839, 883, 917, 989, 1012, 1091, 1114, 1144, 1196, 1226, 1257, 1288, 1359, 1385, 1424, 1458, 1477, 1587, 2864, 2902, 2956, 3051. Analysis calcd for C_82_H_99_CuLaO_4_P_3_ [1444.06]: C 68.20. H 6.91. Found: C 68.60. H 6.64. CCDC: 2383364.

#### Samarium­(III)­tris­[2,4-di-*tert*-butyl-6-(diphenylphosphanyl)­phenolate]­copper­(I) *tert*-Butanolate [Sm^III^(O^t^Bu)­(OAr^P^-1κ^1^ *O*,2κ^1^ *P*)_3_Cu^I^] **4-Sm**

The complex was prepared via the same procedure that was employed for **4-La** using **2-Sm** (308 mg, 201 μmol) and potassium *tert*-butanolate (24 mg, 214 μmol), yielding **4-Sm** as a colorless crystalline solid (164 mg, 56%). Mp 169 °C (dec.). ^1^H NMR (δ in ppm, benzene-*d*_6_, 298 K): −0.15 (s, 27H, ^*t*^ *Bu*), 1.12 (s, 27H, ^*t*^ *Bu*), 4.06 (s, 9H, ^*t*^ *Bu*), 6.34 (t, ^3^ *J*_(1)H,(1)H_ = 8 Hz, 6H, Ar*H*), 6.47 (t, ^3^ *J*_(1)H,(1)H_ = 7 Hz, 6H, Ar*H*), 6.83 (dt, ^3^ *J*_(31)P,(1)H_ = 6 Hz, ^4^ *J*_(1)H,(1)H_ = 3 Hz, 3H, Ar*H*), 6.85 (d, ^4^ *J*_(1)H,(1)H_ = 3 Hz, Ar*H*), 6.91–6.97 (m, 6H, Ar*HH*), 7.13–7.20 (m, 6H, Ar*HH*), 7.25 (t, ^3^ *J*_(1)H,(1)H_ = 7 Hz, 3H, Ar*H*), 8.21 (br s, 6H, Ar*H*). ^13^C­{^1^H} NMR (δ in ppm, benzene-*d*_6_, 298 K): 29.4 (^*t*^ *Bu*), 31.9 (^*t*^ *Bu*), 32.9 (^*t*^ *Bu*), 34.4 (^*t*^ *Bu*), 36.3 (^*t*^ *Bu*), 78.0 (^*t*^ *BuO*), 126.1 (*C*_*Ar*_), 126.5 (*C*_*Ar*_), 129.3 (*C*_*Ar*_), 130.2 (*C*_*Ar*_), 132.2 (*C*_*Ar*_), 135.5 (*C*_*Ar*_), 136.4 (*C*_*Ar*_), 137.5 (*C*_*Ar*_). ^31^P­{^1^H} NMR (δ in ppm, benzene-*d*_6_, 298 K): −3.6. μ_eff_ = 1.1 μ_B_. UV–vis (λ nm, ε L mol^–1^cm^–1^ in brackets): 310 (1.8 × 10^4^). IR (ATR, cm^–1^): 425, 448, 465, 496, 510, 578, 628, 690, 744, 775, 840, 883, 917, 998, 1026, 1091, 1114, 1144, 1156, 1195, 1226, 1257, 1287, 1359, 1385, 1424, 1458, 1475, 1587, 2864, 2902, 2949, 3050. Analysis calcd for C_82_H_99_CuO_4_P_3_Sm [1455.52]: C 67.67. H 6.86. Found: C 68.12. H 6.44. CCDC: 2381886.

#### Yttrium­(III)­tris­[2,4-di-*tert*-butyl-6-(diphenylphosphanyl)­phenolate]­silver­(I) *tert*-Butanolate [Y^III^(O^t^Bu)­(OAr^P^-1κ^1^ *O*,2κ^1^ *P*)_3_Cu^I^] **4-Y**

The complex was prepared via the same procedure that was employed for **4-La** using **2-Y** (252 mg, 171 μmol) and potassium *tert*-butanolate (22 mg, 196 μmol), yielding **4-Y** as a colorless crystalline solid (140 mg, 59%). Mp 173 °C (dec.). ^1^H NMR (δ in ppm, benzene-*d*_6_, 298 K): 1.23 (s, 27H, ^*t*^ *Bu*), 1.47 (s, 27H, ^*t*^ *Bu*), 1.62 (s, 9H, ^*t*^ *Bu*), 6.68 (dt, ^3^ *J*_(31)P,(1)H_ = 6 Hz, ^4^ *J*_(1)H,(1)H_ = 3 Hz, 3H, Ar*H*), 6.77 (t, ^3^ *J*_(1)H,(1)H_ = 7 Hz, 6H, Ar*H H*), 6.82 (t, ^3^ *J*_(1)H,(1)H_ = 8 Hz, 6H, Ar*H H*), 6.89 (t, ^3^ *J*_(1)H,(1)H_ = 7 Hz, 3H, Ar*H H*), 6.97 (t, ^3^ *J*_(1)H,(1)H_ = 7 Hz, 3H, Ar*H H*)*H*, 7.07–7.13 (m, 6H, Ar*H*), 7.16 (s, 3H, Ar*H*), 7.44 (d, ^4^ *J*_(1)H,(1)H_ = 3 Hz, 6H, Ar*H H*). ^13^C­{^1^H} NMR (δ in ppm, benzene-*d*_6_, 298 K): 30.5 (^*t*^ *Bu*), 31.7 (^*t*^ *Bu*), 34.1 (^*t*^ *Bu*), 34.5 (^*t*^ *Bu*), 71.9 (^*t*^ *BuO*), 126.4 (*C*_*Ar*_), 126.6 (*C*_*Ar*_), 128.5 (*C*_*Ar*_), 128.5 (*C*_*Ar*_), 129.5 (*C*_*Ar*_), 132.3 (*C*_*Ar*_), 135.4 (*C*_*Ar*_), 138.0 (*C*_*Ar*_). ^31^P­{^1^H} NMR (δ ppm, benzene-*d*_6_): −7.2. UV–vis (λ nm, ε L mol^–1^cm^–1^ in brackets): 310 (2.2 × 10^4^). IR (ATR, cm^–1^): 432, 466, 497, 512, 530, 580, 628, 690, 743, 774, 843, 883, 918, 1010, 1091, 1115, 1145, 1200, 1226, 1258, 1288, 1359, 1385, 1426, 1460, 1475, 2863, 2902, 2948, 3051. Analysis calcd for C_82_H_99_CuO_4_P_3_Y [1394.06]: C 70.65. H 7.16. Found: C 70.22. H 6.67. CCDC: 2381889.

#### Ytterbium­(III)­tris­[2,4-di-*tert*-butyl-6-(diphenylphosphanyl)­phenolate]­silver­(I) *tert*-Butanolate [Yb^III^(O^t^Bu)­(OAr^P^-1κ^1^ *O*,2κ^1^ *P*)_3_Cu^I^] **4-Yb**

The complex was prepared via the same procedure that was employed for **4-La** using **2-Yb** (314 mg, 202 μmol) and potassium *tert*-butanolate (23 mg, 205 μmol), yielding **4-Yb** as a colorless crystalline solid (183 mg, 61%). Mp 219 °C (dec.). ^1^H NMR (δ in ppm, benzene-*d*_6_, 298 K): −54.66 (s, 9H, ^*t*^ *Bu*), −10.78 (s, 6H, Ar*H*), −1.02 (br s, 6H, Ar*H*), 0.81 (s, 3H, Ar*H*), 1.72 (s, 3H, Ar*H*), 3.49 (s, 27H, ^*t*^ *Bu*), 11.49 (br s, 6H, Ar*H*), 15.15 (s, 3H, Ar*H*), 15.55 (s, 6H, Ar*H*), 19.97 (s, 3H, Ar*H*), 33.24 (s, 27H, ^*t*^ *Bu*). ^31^P­{^1^H} NMR (δ in ppm, benzene-*d*_6_, 298 K): −57.0. μ_eff_ = 3.8 μ_B_. UV–vis (λ nm, ε L mol^–1^cm^–1^ in brackets): 310 (2.1 × 10^4^), 347 (7.4 × 10^3^, sh). IR (ATR, cm^–1^): 427, 448, 466, 497, 512, 531, 580, 626, 690, 743, 774, 845, 883, 918, 1015, 1091, 1117, 1146, 1200, 1226, 1258, 1288, 1360, 1386, 1426, 1460, 1477, 1587, 2863, 2902, 2949, 3051. Analysis calculated for C_82_H_99_CuO_4_P_3_Yb [1478.20]: C 66.63. H 6.75. Found: C 66.81. H 6.43. CCDC: 2381888.

#### [(cod)_2_Cu^I^OTf][Bibr ref23]

A Schlenk flask equipped with a stir bar was charged with [(C_7_H_8_)­(Cu^I^OTf)_2_] (2.59 g, 5.00 mmol) and toluene (30 mL). At ambient temperature, 1,5-cyclooctadiene (3.25 g, 30 mmol) was gradually added via syringe under stirring. After the addition, the white suspension was stirred for 1 h followed by the removal of the supernatant solution using a filter cannula and washing of the colorless crystalline solid three times with pentane. The product was then dried under reduced pressure, yielding [(cod)_2_Cu^I^OTf] as a colorless crystalline solid (3.93 g, 92%). ^1^H NMR (δ in ppm, chloroform-*d*, 298 K): 2.41 (s, 16H, C*H*_2_), 5.78 (s, 8H, C*H*). ^13^C­{^1^H} NMR (δ in ppm, chloroform-*d*, 298 K): 28.1 (*C*H_2_), 123.2 (*C*H).

## Supplementary Material



## Notes

Crystallographic data, NMR, UV–vis, and IR spectroscopic data of the presented compounds (PDF)

## References

[1] Cotton, F. A. ; Murillo, C. A. ; Walton, R. A. Multiple Bonds between Metal Atoms; Springer Science and Business Media: New York, NY, 2005.

[2] Buchwalter P., Rosé J., Braunstein P. (2015). Multimetallic Catalysis Based on
Heterometallic Complexes and Clusters. Chem.
Rev..

[3] Shibasaki, M. Multimetallic Catalysts in Organic Synthesis, 1st ed.; John Wiley & Sons, Incorporated: Hoboken, 2006.

[4] Thomas C. M. (2011). Metal-Metal
Multiple Bonds in Early/Late Heterobimetallic Complexes: Applications
toward Small Molecule Activation and Catalysis. Comments Inorg. Chem..

[5] Cooper B. G., Napoline J. W., Thomas C. M. (2012). Catalytic
Applications of Early/Late
Heterobimetallic Complexes. Catal. Rev..

[6] Liddle S. T., Mills D. P. (2009). Metal–Metal Bonds in f-Element Chemistry. Dalton Trans..

[7] Butovskii M. V., Tok O. L., Wagner F. R., Kempe R. (2008). Bismetallocenes: Lanthanoid-Transition-Metal
Bonds through Alkane Elimination. Angew. Chem.,
Int. Ed..

[8] Butovskii M. V., Döring C., Bezugly V., Wagner F. R., Grin Y., Kempe R. (2010). Molecules Containing Rare-Earth Atoms Solely Bonded by Transition
Metals. Nat. Chem..

[9] Döring C., Dietel A.-M., Butovskii M. V., Bezugly V., Wagner F. R., Kempe R. (2010). Molecular [Yb­(TM)_2_] Intermetalloids (TM = Ru, Re). Chem.
- Eur. J..

[10] Butovskii M. V., Tok O. L., Bezugly V., Wagner F. R., Kempe R. (2011). Molecular
Lanthanoid-Transition-Metal Cluster through C-H Bond Activation by
Polar Metal-Metal Bonds. Angew. Chem., Int.
Ed..

[11] Völcker F., Mück F. M., Vogiatzis K. D., Fink K., Roesky P. W. (2015). Bi- and
Trimetallic Rare-Earth–Palladium Complexes Ligated by Phosphinoamides. Chem. Commun..

[12] Völcker F., Roesky P. W. (2016). Bimetallic Rare-Earth/Platinum
Complexes Ligated by
Phosphinoamides. Dalton Trans..

[13] Ramirez B.
L., Sharma P., Eisenhart R. J., Gagliardi L., Lu C. C. (2019). Bimetallic Nickel-Lutetium
Complexes: Tuning the Properties and Catalytic
Hydrogenation Activity of the Ni Site by Varying the Lu Coordination
Environment. Chem. Sci..

[14] Du J., Huang Z., Zhang Y., Wang S., Zhou S., Fang H., Cui P. (2019). A Scandium
Metalloligand-Based Heterobimetallic
Pd–Sc Complex: Electronic Tuning Through a Very Short Pd→Sc
Dative Bond. Chem. - Eur. J..

[15] Du J., Zhang Y., Huang Z., Zhou S., Fang H., Cui P. (2020). Heterobimetallic Pd(0) Complexes with Pd→Ln (Ln = Sc, Y, Yb,
Lu) Dative Bonds: Rare-Earth Metal-Dominated Frustrated Lewis Pair-like
Reactivity. Dalton Trans..

[16] Cui P., Wu C., Du J., Luo G., Huang Z., Zhou S. (2021). Three-Coordinate
Pd­(0) with Rare-Earth Metalloligands: Synergetic CO Activation and
Double P–C Bond Cleavage-Formation Reactions. Inorg. Chem..

[17] Ma, L. ; Hong, D. ; Cui, P. Hydrosilylation of Terminal Alkenes Catalyzed by a Scandium Metalloligand Supported Ni(0) Complex. Eur. J. Org. Chem. 2025 28 e202500069 10.1002/ejoc.202500069.

[18] Hlina J. A., Pankhurst J. R., Kaltsoyannis N., Arnold P. L. (2016). Metal–Metal
Bonding in Uranium–Group 10 Complexes. J. Am. Chem. Soc..

[19] Hlina J. A., Wells J. A. L., Pankhurst J. R., Love J. B., Arnold P. L. (2017). Uranium
Rhodium Bonding in Heterometallic Complexes. Dalton Trans..

[20] Sinclair F., Hlina J. A., Wells J. A. L., Shaver M. P., Arnold P. L. (2017). Ring Opening
Polymerisation of Lactide with Uranium­(IV) and Cerium­(IV) Phosphinoaryloxide
Complexes. Dalton Trans..

[21] Haidinger A., Dilly C. I., Fischer R. C., Svatunek D., Uher J. M., Hlina J. A. (2023). To Bond or Not to
Bond: Metal–Metal Interaction
in Heterobimetallic Rare-Earth Metal–Silver Complexes. Inorg. Chem..

[22] Alexopoulos E., Liu Y., Bowles A. W. J., Réant B. L. L., Ortu F. (2024). Synthesis and Characterisation
of Phosphino-Aryloxide Rare Earth Complexes. Molecules.

[23] Solomon R. G., Kochi J. K. (1972). Cationic Benzene and Olefin Complexes of Copper­(I)
Trifluoromethanesulphonate. J. Chem. Soc. Chem.
Commun..

[24] Cordero B., Gómez V., Platero-Prats A. E., Revés M., Echeverría J., Cremades E., Barragán F., Alvarez S. (2008). Covalent Radii Revisited. Dalton
Trans..

[25] Schubert E. M. (1992). Utilizing
the Evans Method with a Superconducting NMR Spectrometer in the Undergraduate
Laboratory. J. Chem. Educ..

[26] Cotton, S. Lanthanide and Actinide Chemistry: Cotton/Lanthanide and Actinide Chemistry; John Wiley & Sons, Ltd: Chichester, UK, 2006.

[27] Michl J., West R. (2000). Conformations of Linear Chains. Systematics and Suggestions for Nomenclature. Acc. Chem. Res..

[28] Pregosin, P. S. NMR in Organometallic Chemistry; Wiley-VCH Verlag GmbH & Co., KGaA: Weinheim, 2012.

[29] Bakewell C., Cao T.-P.-A., Le
Goff X. F., Long N. J., Auffrant A., Williams C. K. (2013). Yttrium
Phosphasalen Initiators for *Rac* -Lactide Polymerization. Organometallics.

[30] Carnall W. T., Fields P. R., Rajnak K. (1968). Electronic
Energy Levels in the Trivalent
Lanthanide Aquo Ions. I. Pr^3+^, Nd^3+^, Pm^3+^, Sm^3+^, Dy^3+^, Ho^3+^, Er^3+^, and Tm^3+^. J. Chem. Phys..

[31] Faulkner S., Natrajan L. S., Perry W. S., Sykes D. (2009). Sensitised Luminescence
in Lanthanide Containing Arrays and d–f Hybrids. Dalton Trans..

[32] S
Natrajan L. (2011). Hetero-Polymetallic Complexes Incorporating Luminescent
Lanthanide Ions. Curr. Inorg. Chem..

[33] Bienz, S. ; Bigler, L. ; Fox, T. ; Meier, H. ; Hesse, M. ; Zeeh, B. Spektroskopische Methoden in der Organischen Chemie. In überarbeitete und erweiterte Auflage, 9th ed.; Georg Thieme Verlag: Stuttgart New York, 2016.

[34] Johnston D. H., Shriver D. F. (1993). Vibrational Study
of the Trifluoromethanesulfonate
Anion: Unambiguous Assignment of the Asymmetric Stretching Modes. Inorg. Chem..

[35] Pangborn A. B., Giardello M. A., Grubbs R. H., Rosen R. K., Timmers F. J. (1996). Safe and
Convenient Procedure for Solvent Purification. Organometallics.

[36] Schuetz S. A., Day V. W., Sommer R. D., Rheingold A. L., Belot J. A. (2001). Anhydrous Lanthanide Schiff Base
Complexes and Their
Preparation Using Lanthanide Triflate Derived Amides. Inorg. Chem..

[37] Flores D. M., Schmidt V. A. (2019). Intermolecular 2
+ 2 Carbonyl–Olefin Photocycloadditions
Enabled by Cu­(I)–Norbornene MLCT. J.
Am. Chem. Soc..

[38] Bain G. A., Berry J. F. (2008). Diamagnetic Corrections
and Pascal’s Constants. J. Chem. Educ..

[39] SAINTPLUS: Software Reference Manual, Version 6.45 1997–2003; Bruker-AXS: Madison, WI.

[40] Blessing R.
H. (1995). An Empirical
Correction for Absorption Anisotropy. Found.
Crystallogr..

[41] Sheldrick, G. M. SADABS, Version 2.10; Bruker AXS Inc.: Madison, WI, 2003.

[42] Rigaku Oxford Diffraction. CrysAlisPro; Rigaku Oxford Diffraction, 2024.

[43] Sheldrick G. M. (2008). A Short
History of *SHELX*. Found. Crystallogr..

[44] Fleißner, A. ; Rehbein, V. ; Haidinger, A. ; Dilly, C. I. ; Dupé, A. ; Fischer, R. C. ; Hecht, E. S. ; Hlina, J. A. Synthesis and Characterisation of Phosphanophenolate-Based Rare-Earth Metal-Copper Complexes Inorg. Chem. 10.26434/chemrxiv-2025-p0s00-v2. PMC12606715 40878002

