# Supplementary material for: Synthesis and Characterization of Phosphanophenolate-Based Rare-Earth Metal–Copper Complexes
Source: Inorg Chem. 2025 Aug 28;64(44):21825–33. doi: 10.1021/acs.inorgchem.5c01738 (PMC12606715; doi:10.1021/acs.inorgchem.5c01738)
Supplement: Supplementary file 1 [file ic5c01738_si_001.pdf]

Supporting Information

Synthesis and Characterisation of Phosphanophenolate-based Rare-Earth Metal-Copper Complexes

Andreas Fleißner,[b] Viktoria Rehbein,[b] Alexandra Haidinger,[b] Christina I. Dilly,[b] Antoine Dupé,[a] Roland C. Fischer,[b] Elise S. Hecht,[c] and Johann A. Hlina[a,*]

[a]Institute of Chemistry, Inorganic Chemistry, University of Graz, Schubertstraße 1, 8010 Graz, Austria.

[b]Institute of Inorganic Chemistry, Graz University of Technology, Stremayrgasse 9, 8010 Graz, Austria.

[c]Institute of Analytical Chemistry and Food Chemistry, Graz University of Technology, Stremayrgasse 9, 8010 Graz, Austria.

Corresponding author e-mail: johann.hlina@uni-graz.at

Table of Contents

Crystallography S2

Additional Molecular Structures S2

Crystallographic Data S4

NMR Spectroscopy S7

UV-vis Spectroscopy S21

IR Spectroscopy S26

Selected Derivatisation Attempts S31

Crystallography:

Additional Molecular Structures:

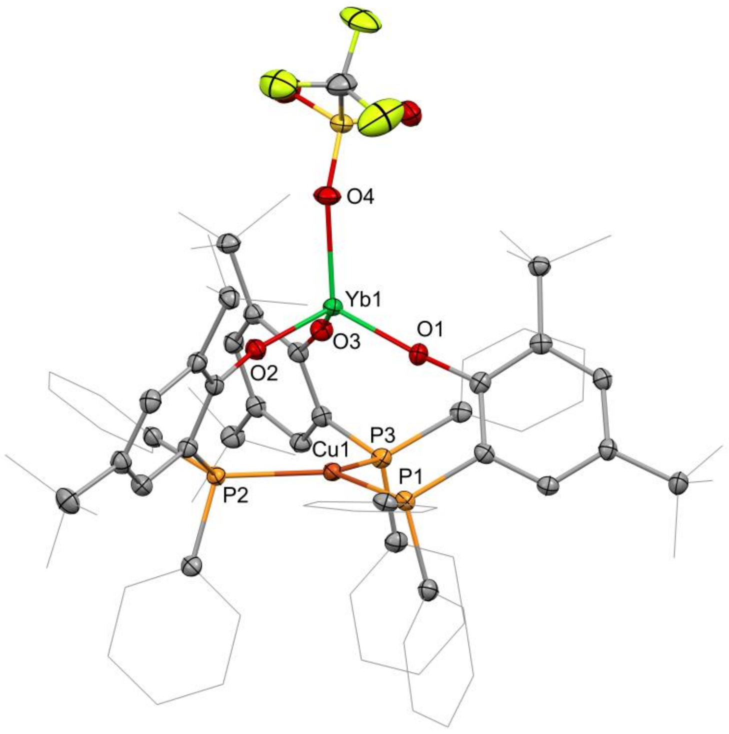

Figure S1. Molecular structure of **2-Yb**. Hydrogen atoms are omitted and selected carbon atoms depicted as wireframe for clarity. Thermal ellipsoids drawn at 50% probability.

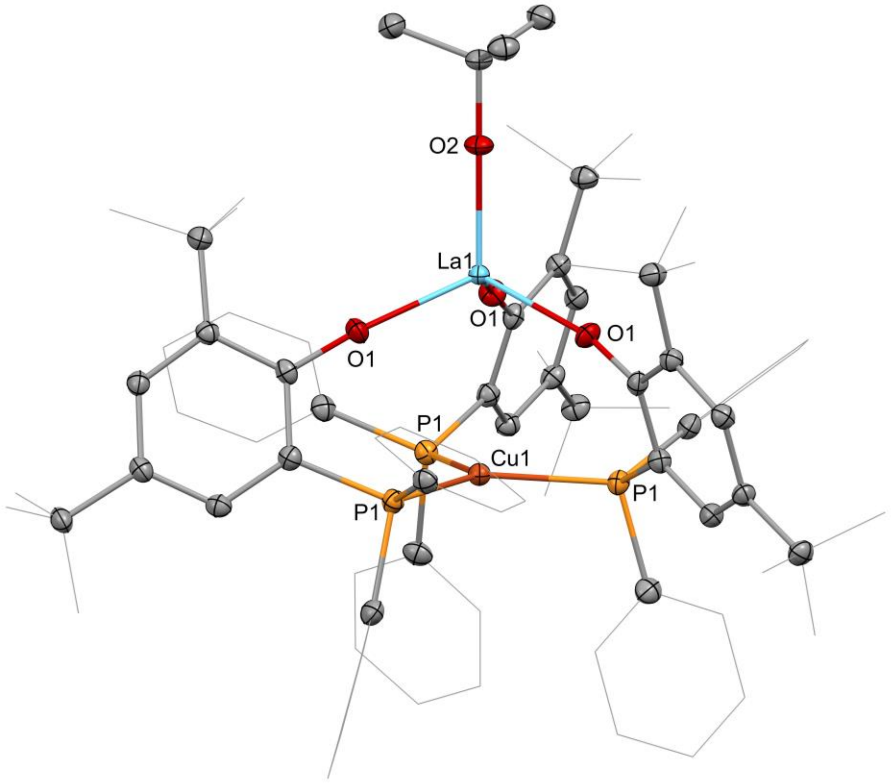

Figure S2. Molecular structure of **4-La**. Hydrogen atoms are omitted and selected carbon atoms depicted as wireframe for clarity. Thermal ellipsoids drawn at 50 % probability.

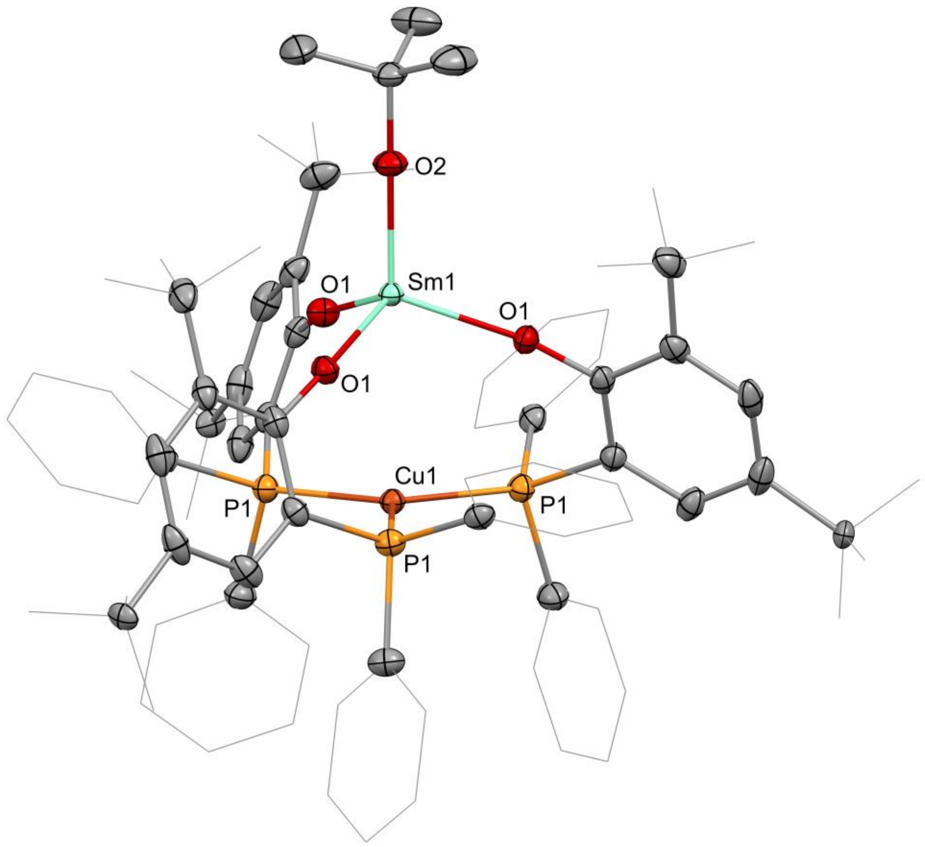

Figure S3. Molecular structure of **4-Sm**. Hydrogen atoms are omitted and selected carbon atoms depicted as wireframe for clarity. Thermal ellipsoids drawn at 50 % probability.

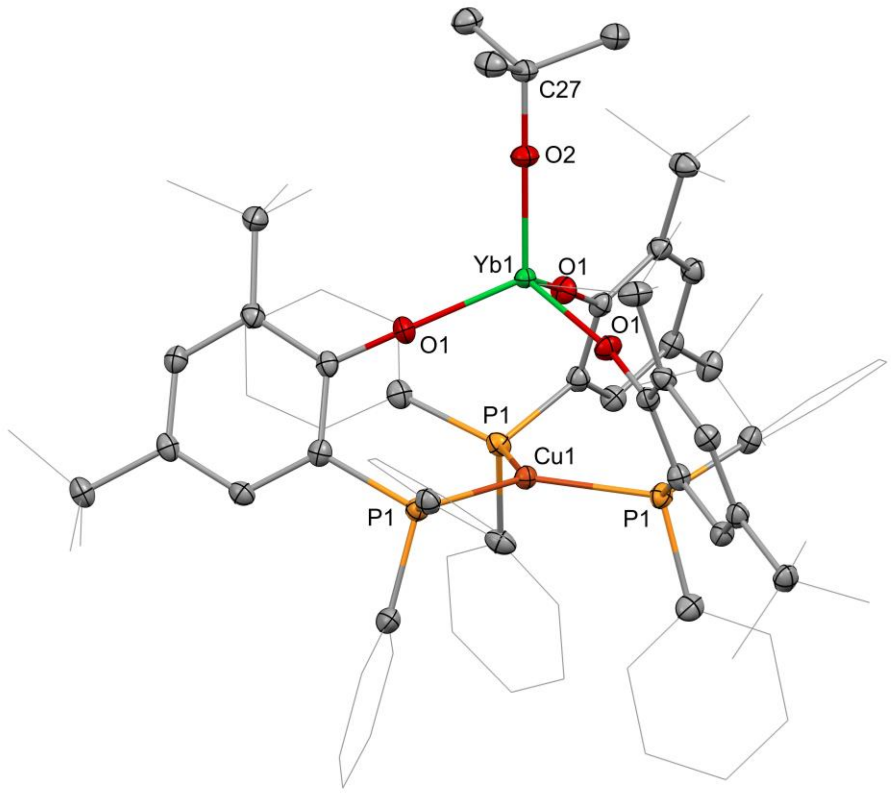

Figure S4. Molecular structure of **4-Yb**. Hydrogen atoms are omitted and selected carbon atoms depicted as wireframe for clarity. Thermal ellipsoids drawn at 50 % probability.

Crystallographic Data:

Table S1. Crystallographic data of **2-La**, **2-Sm**, and **2-Y**.

	**2-La**	**2-Sm**	**2-Y**
CCDC number	2381853	2347686	2381852
Empirical formula	$C_{87}H_{106}CuF_3LaO_8P_3S$	$C_{79}H_{90}CuF_3O_6P_3SSm$	$C_{79}H_{90}CuF_3O_6P_3SY$
Formula weight	1664.13	1531.36	1469.92
Temperature /K	150.00	150.00	100.00
Crystal system	monoclinic	monoclinic	monoclinic
Space group	$P2_1/n$	$P2_1/c$	$P2_1/c$
a /Å	13.781(2)	15.085(3)	15.3199(5)
b /Å	27.688(5)	16.731(3)	16.5678(7)
c /Å	23.819(4)	32.039(6)	31.6499(12)
α /°	90	90	90
β /°	90.674(3)	96.042(3)	96.150(2)
γ /°	90	90	90
Volume /$Å^3$	9088(3)	8042(3)	7987.1(5)
Z	4	4	4
ρ_{calc}g /cm^3	1.216	1.265	1.222
M /mm^{-1}	0.828	1.127	1.130
F(000)	3464.0	3164.0	3072.0
Crystal size /mm^3	0.33 x 0.3 x 0.3	0.24 x 0.13 x 0.1	0.33 x 0.19 x 0.16
2Θ range for data collection /°	2.942 to 52.75	3.528 to 52.726	3.57 to 58.06
Index ranges	-17<=h<=17, -34<=k<=34, -29<=l<=29	-18<=h<=18, -20<=k<=20, -39<=l<=39	-20<=h<=19, -22<=k<=22, -43<=l<=43
Reflections collected	72165	63291	151993
Independent reflections	18552 [R(int) = 0.0529]	16381 [R(int) = 0.0456]	21138 [R(int) = 0.1371]
Data/restraints/parameters	18552 / 0 / 986	16381 / 3 / 896	21138 / 72 / 896
Goodness-of-fit on F^2	1.143	1.079	1.024
Final R indexes [I>=2σ (I)]	R1 = 0.0551, wR2 = 0.1184	R1 = 0.0420, wR2 = 0.0923	R1 = 0.0678, wR2 = 0.1207
Final R indexes [all data]	R1 = 0.0666, wR2 = 0.1236	R1 = 0.0510, wR2 = 0.0961	R1 = 0.1299, wR2 = 0.1377
Largest diff. peak/hole / e $Å^{-3}$	1.18 and -0.82	1.16 and -0.46	0.80 and -0.75

Table S2. Crystallographic data of **2-Yb**, **3**, and **4-La**.

	**2-Yb**	**3**	**4-La**
CCDC number	2381887	2347685	2383364
Empirical formula	$C_{79}H_{90}CuF_3O_6P_3SYb\cdot C_6H_6$	$C_{85}H_{99}CuF_3O_6P_3S$	$C_{82}H_{99}CuO_4P_3La$
Formula weight	1632.16	1462.15	1443.97

Temperature /K	100.0(7)	100.02	100.0(6)
Crystal system	monoclinic	monoclinic	trigonal
Space group	$P2_1/c$	$P2_1/c$	*R*-3
a /Å	15.29880(10)	22.7924(9)	21.0718(2)
b /Å	16.52310(10)	13.1225(4)	21.0718(2)
c /Å	31.5625(2)	26.1369(11)	30.0773(2)
α /°	90	90	90
β /°	96.4390(10)	92.414(2)	90
γ /°	90	90	120
Volume /Å^3	7928.15(9)	7810.4(5)	11565.7(2)
Z	4	4	6
ρ_{calc} g /cm^3	1.367	1.179	1.244
M /mm^{-1}	3.739	0.430	5.502
F(000)	3364.0	2928.0	4524.0
Crystal size /mm^3	0.15 x 0.09 x 0.06	0.29 x 0.16 x 0.14	0.09 x 0.07 x 0.06
2Θ range for data collection /°	5.636 to 154.746	3.474 to 60.012	7.618 to 154.706
Index ranges	-19<=h<=19,<br>-19<=k<=20,<br>-35<=l<=39	-28<=h<=28,<br>-16<=k<=16,<br>-33<=l<=33	-26<=h<=26,<br>-24<=k<=23,<br>-34<=l<=37
Reflections collected	91018	310014	28279
Independent reflections	16800 [R(int) = 0.0401]	16585 [R(int) = 0.0712]	5448 [R(int) = 0.0303]
Data/restraints/parameters	16800 / 198 / 999	16585 / 0 / 903	5448 / 0 / 281
Goodness-of-fit on F^2	1.118	1.025	1.068
Final R indexes [I>=2σ (I)]	R1 = 0.0314, wR2 = 0.0774	R1 = 0.0363, wR2 = 0.0837	R1 = 0.0209, wR2 = 0.0527
Final R indexes [all data]	R1 = 0.0347, wR2 = 0.0787	R1 = 0.0529, wR2 = 0.0940	R1 = 0.0212, wR2 = 0.0528
Largest diff. peak/hole / e Å^{-3}	0.84 and -0.62	0.44 and -0.74	0.34 and -0.48

Table S3. Crystallographic data of **4-Sm**, **4-Y** and **4-Yb**.

	**4-Sm**	**4-Y**	**4-Yb**
CCDC number	2381886	2381889	2381888
Empirical formula	$C_{82}H_{99}CuO_4P_3Sm$	$C_{82}H_{99}CuO_4P_3Y$	$C_{82}H_{99}CuO_4P_3Yb$
Formula weight	1455.41	1478,10	1393.97
Temperature /K	100.0(3)	100.0(2)	100.0(8)
Crystal system	trigonal	trigonal	trigonal
Space group	*R*-3	*R*-3	*R*-3
a /Å	21.1004(4)	21.01250(10)	21.03620(10)
b /Å	21.1004(4)	21.01250(10)	21.03620(10)
c /Å	29.9665(5)	29.9928(3)	30.0751(2)

α /°	90	90	90
β /°	90	90	90
γ /°	120	120	120
Volume /$Å^3$	11554.4(5)	11468.40(16)	11525.83(13)
Z	6	6	6
$ρ_{calc}$ g /cm^3	1.255	1.284	1.205
M /mm^{-1}	1.140	3.493	2.294
F(000)	4554.0	4602.0	4416.0
Crystal size /mm^3	0.15 × 0.12 × 0.10	0.12 x 0.07 x 0.06	0.12 x 0.09 x 0.07
2Θ range for data collection /°	2.808 to 30.503	5.68 to 154.706	7.624 to 154.706
Index ranges	-30<=h<=30,<br>-30<=k<=30,<br>-42<=l<=42	-26<=h<=25,<br>-24<=k<=26,<br>-36<=l<=37	-26<=h<=26,<br>-26<=k<=24,<br>-36<=l<=38
Reflections collected	18907	28362	28409
Independent reflections	7840 [R(int) = 0.0295]	5388 [R(int) = 0.0313]	5426 [R(int) = 0.0262]
Data/restraints/parameters	7840 / 10 / 321	5388 / 0 / 281	5426 / 0 / 281
Goodness-of-fit on F^2	1.050	1.055	1.071
Final R indexes [I>=2σ (I)]	R1 = 0.0292, wR2 = 0.0626	R1 = 0.0203, wR2 = 0.0506	R1 = 0.0242, wR2 = 0.0619
Final R indexes [all data]	R1 = 0.0359, wR2 = 0.0645	R1 = 0.0213, wR2 = 0.0511	R1 = 0.0262, wR2 = 0.0628
Largest diff. peak/hole / e$Å^{-3}$	0.427 and -0.469	0.31 and -0.42	0.31 and -0.47

NMR Spectroscopy:

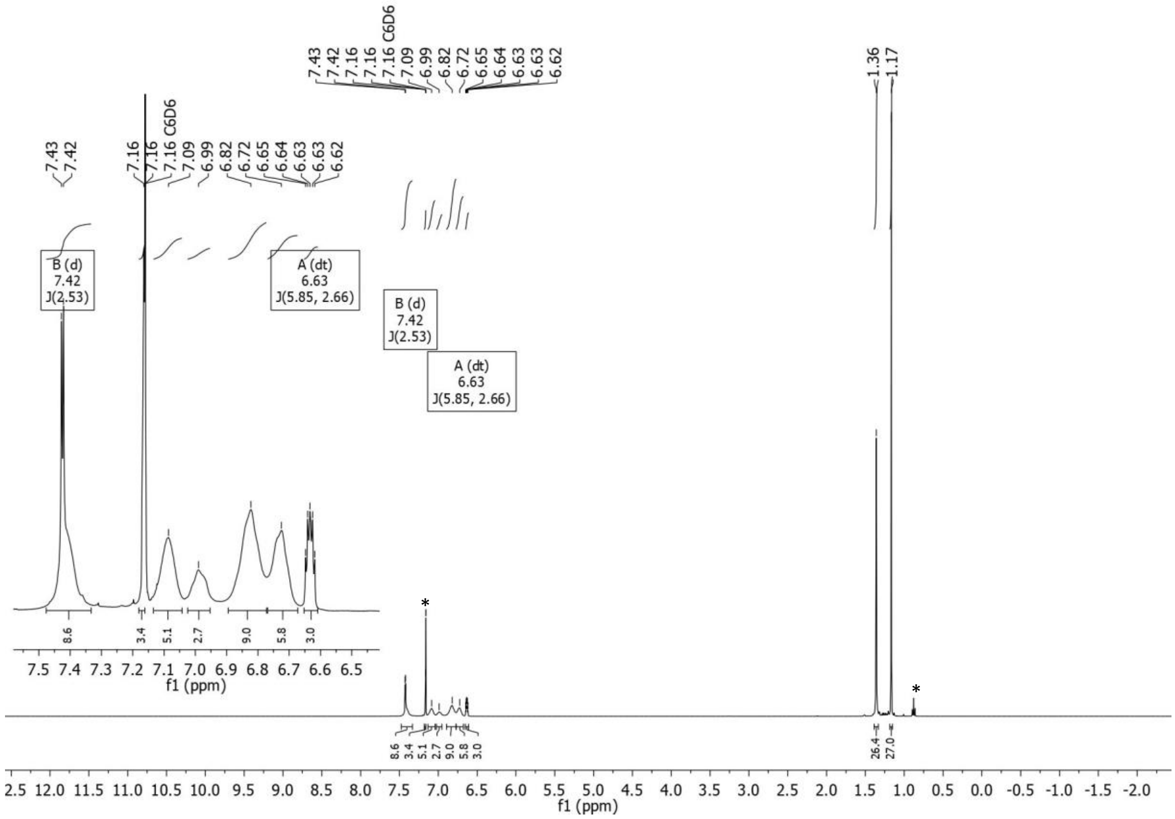

Figure S5. ^{1}H NMR spectrum of **2-La** in C_6D_6 recorded at 298 K. NMR solvent and trace impurities are marked with an asterisk.

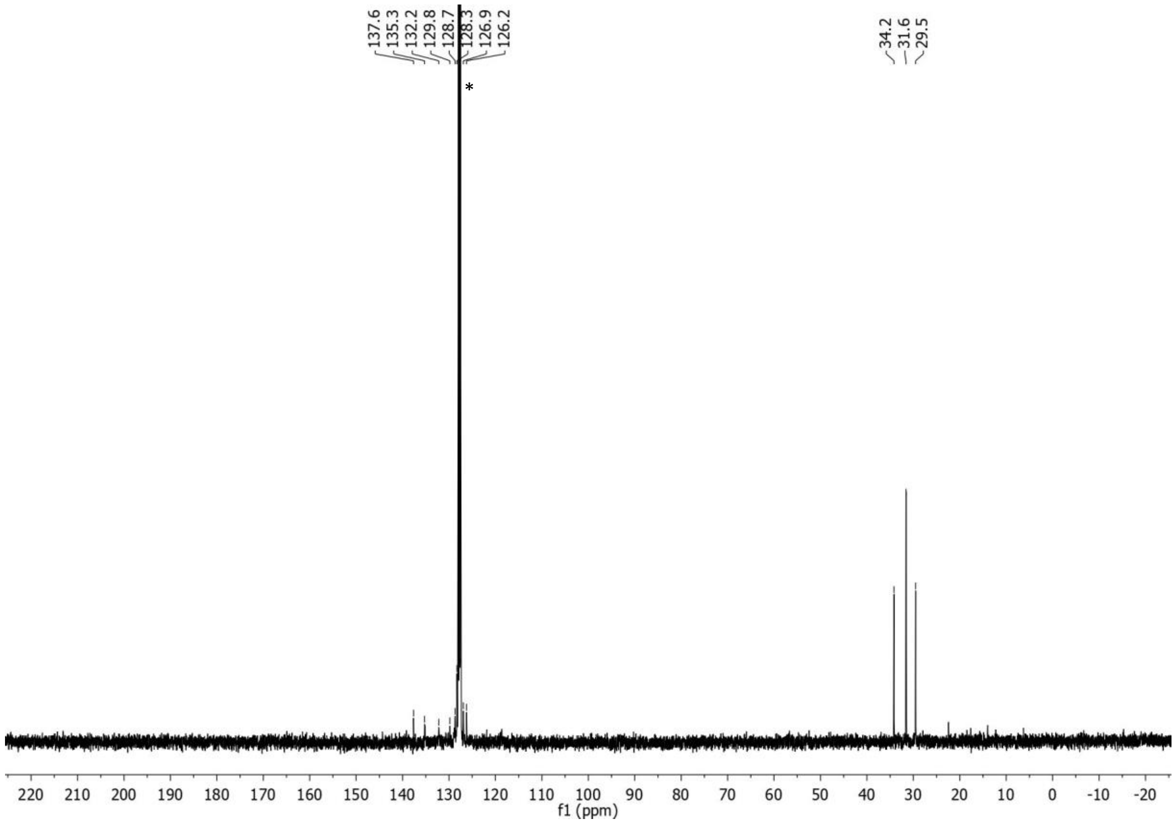

Figure S6. $^{13}C\{^{1}H\}$ NMR spectrum of **2-La** in C_6D_6 recorded at 298 K. NMR solvent is marked with an asterisk.

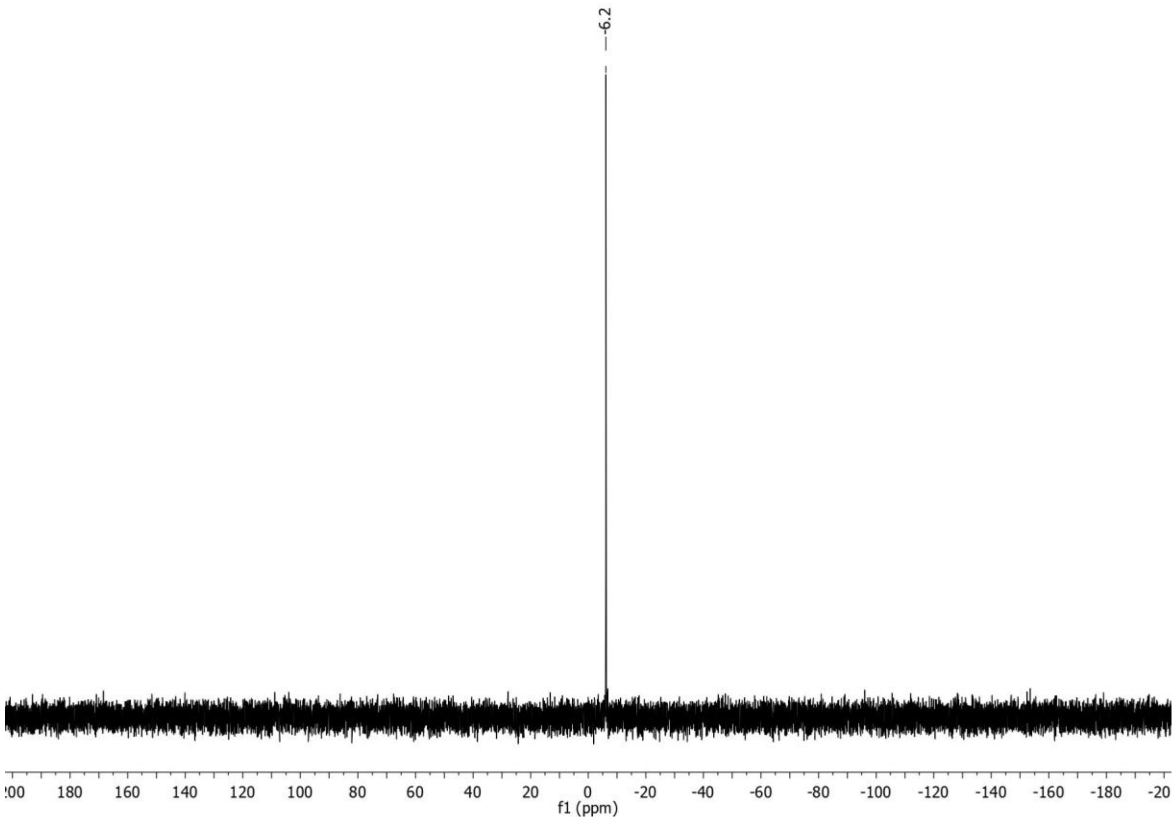

Figure S7. $^{31}P\{^1H\}$ NMR spectrum of **2-La** in C_6D_6 recorded at 298 K.

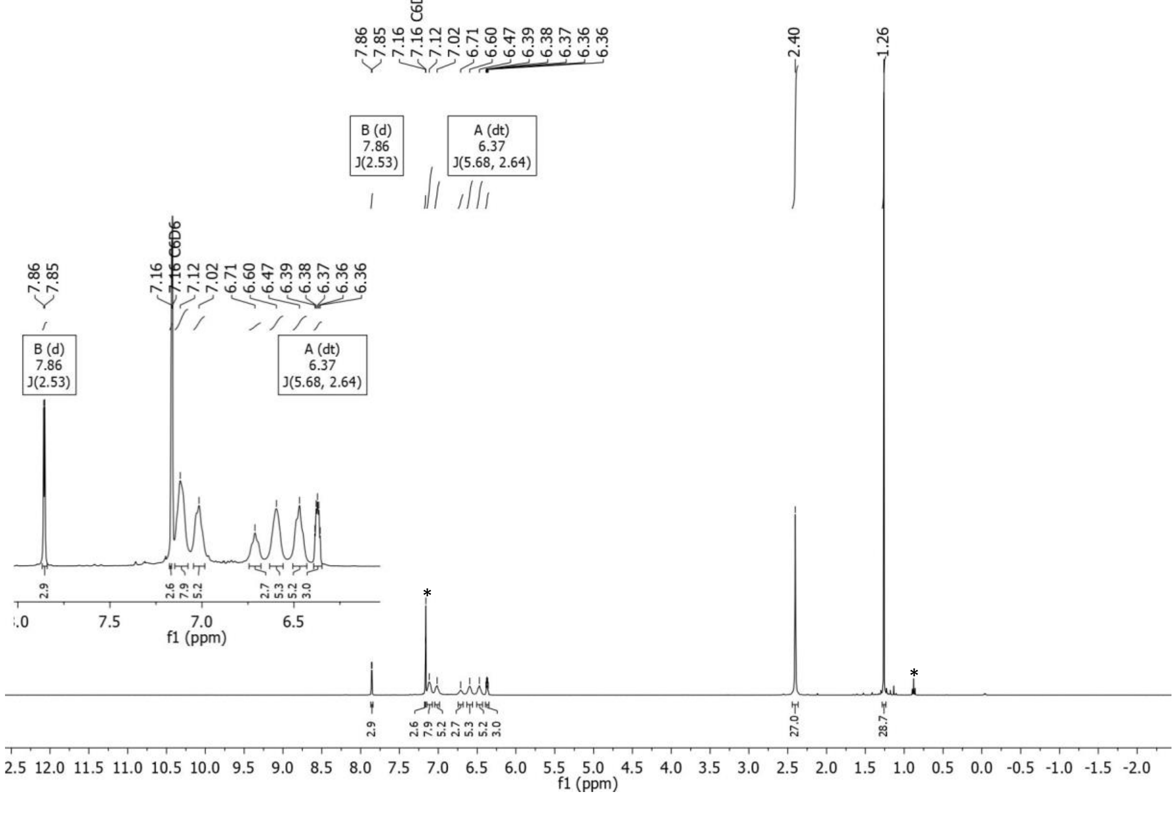

Figure S8. ^1H NMR spectrum of **2-Sm** in C_6D_6 recorded at 298 K. NMR solvent and trace impurities are marked with an asterisk.

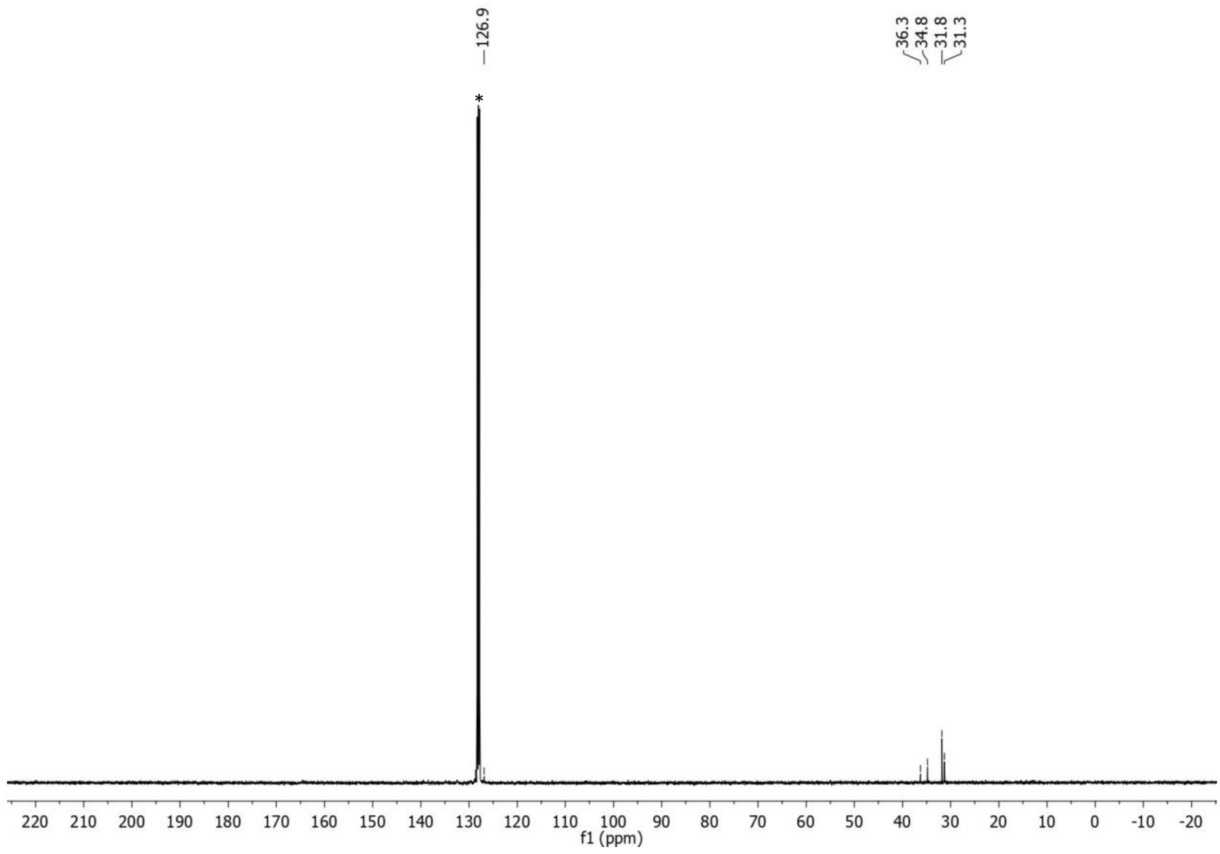

Figure S9. $^{13}C\{^{1}H\}$ NMR spectrum of **2-Sm** in C_6D_6 recorded at 298 K. NMR solvent is marked with an asterisk.

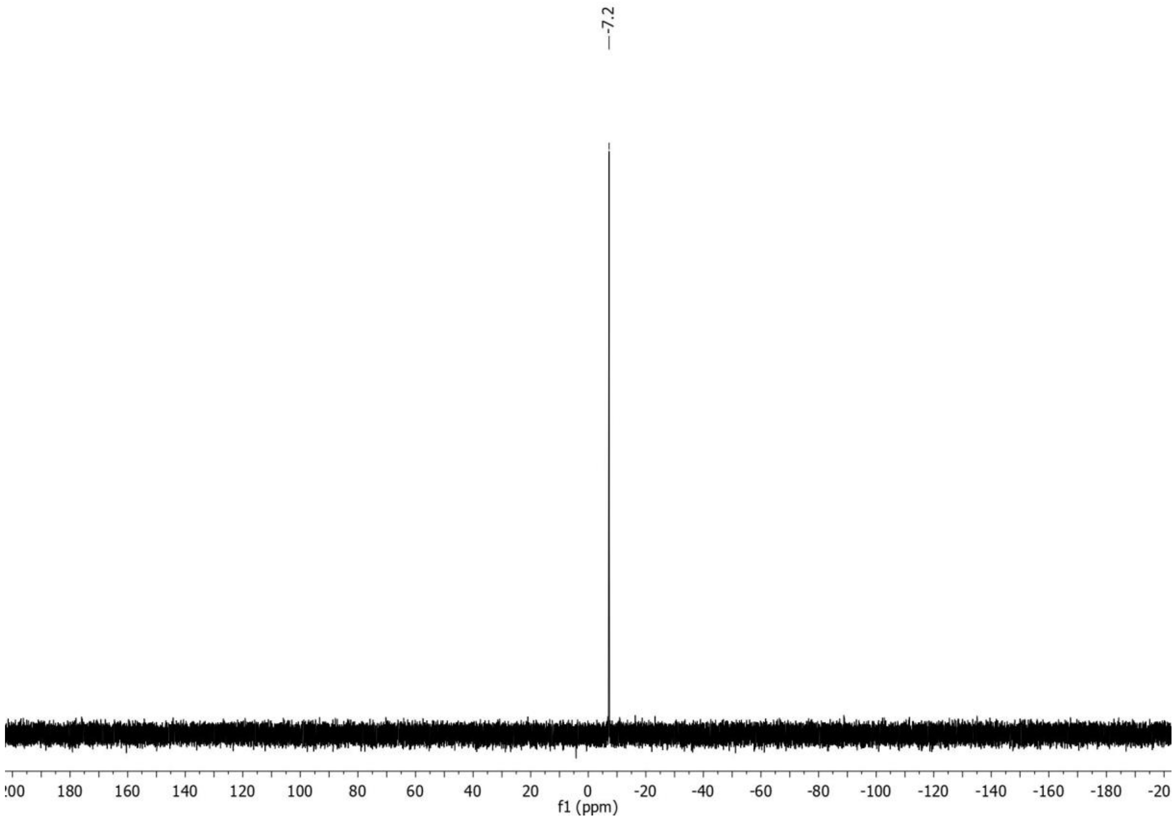

Figure S10. $^{31}P\{^{1}H\}$ NMR spectrum of **2-Sm** in C_6D_6 recorded at 298 K.

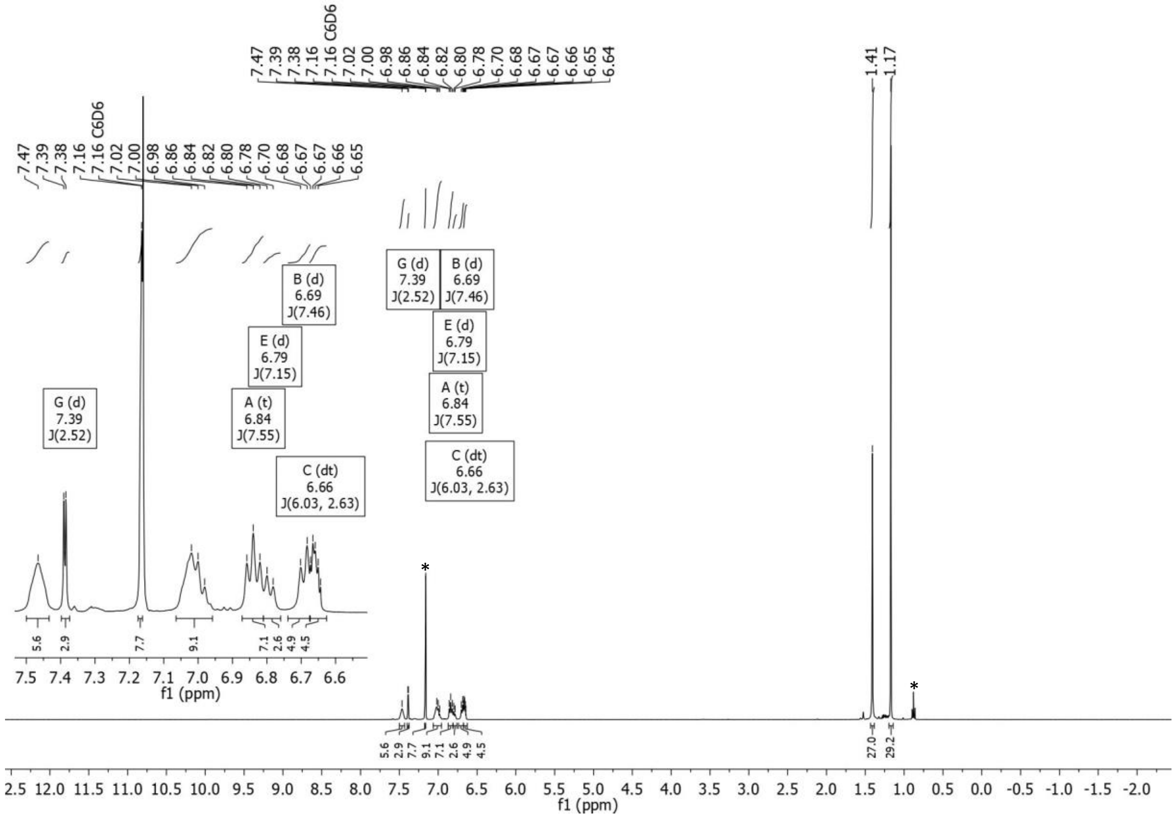

Figure S11. ^{1}H NMR spectrum of **2-Y** in C_6D_6 recorded at 298 K. NMR solvent and trace impurities are marked with an asterisk.

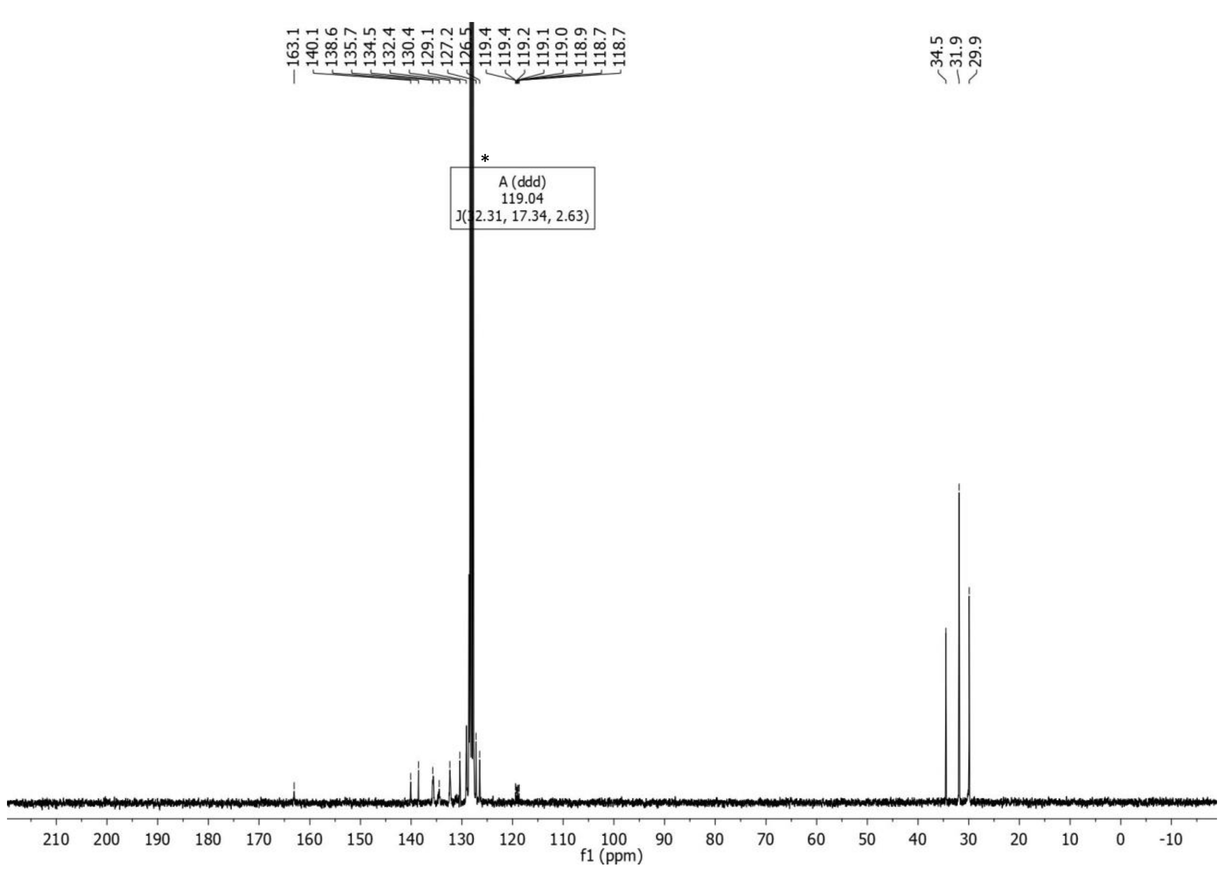

Figure S12. $^{13}C\{^{1}H\}$ NMR spectrum of **2-Y** in C_6D_6 recorded at 298 K. NMR solvent and trace impurities are marked with an asterisk.

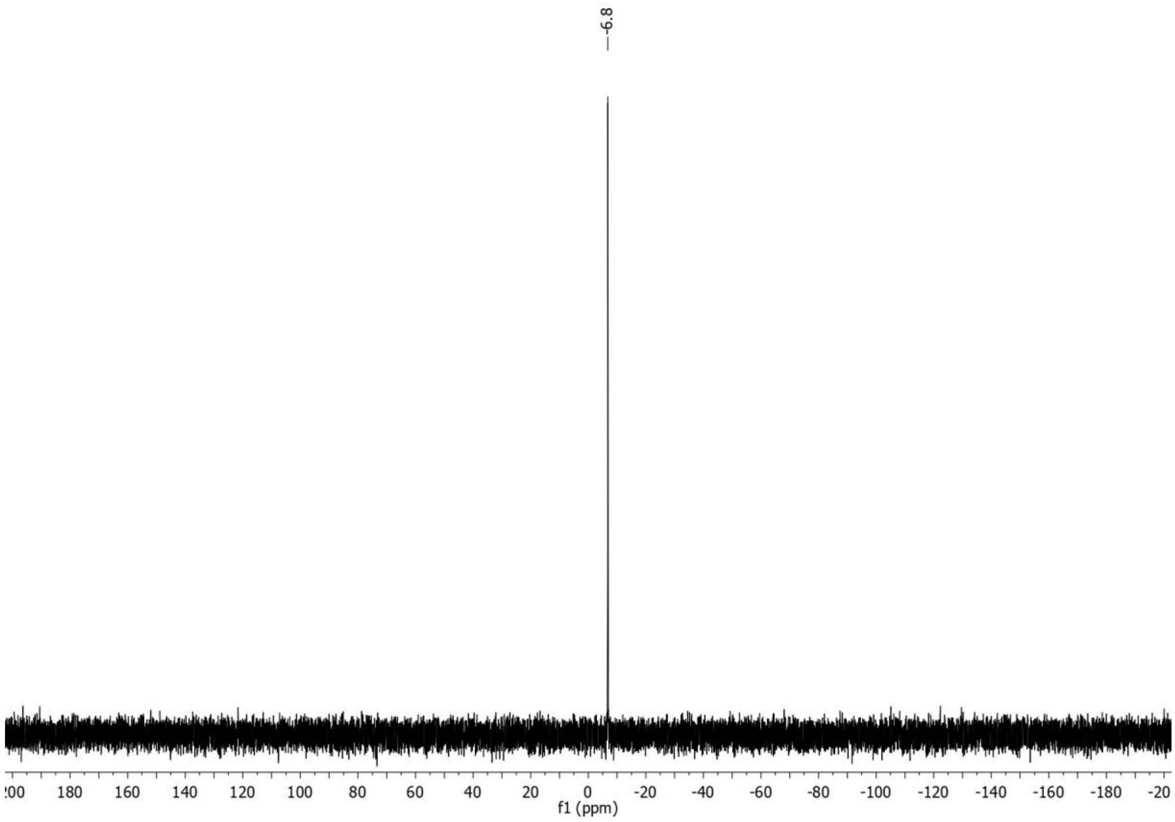

Figure S13. $^{31}P\{^{1}H\}$ NMR spectrum of **2-Y** in C_6D_6 recorded at 298 K.

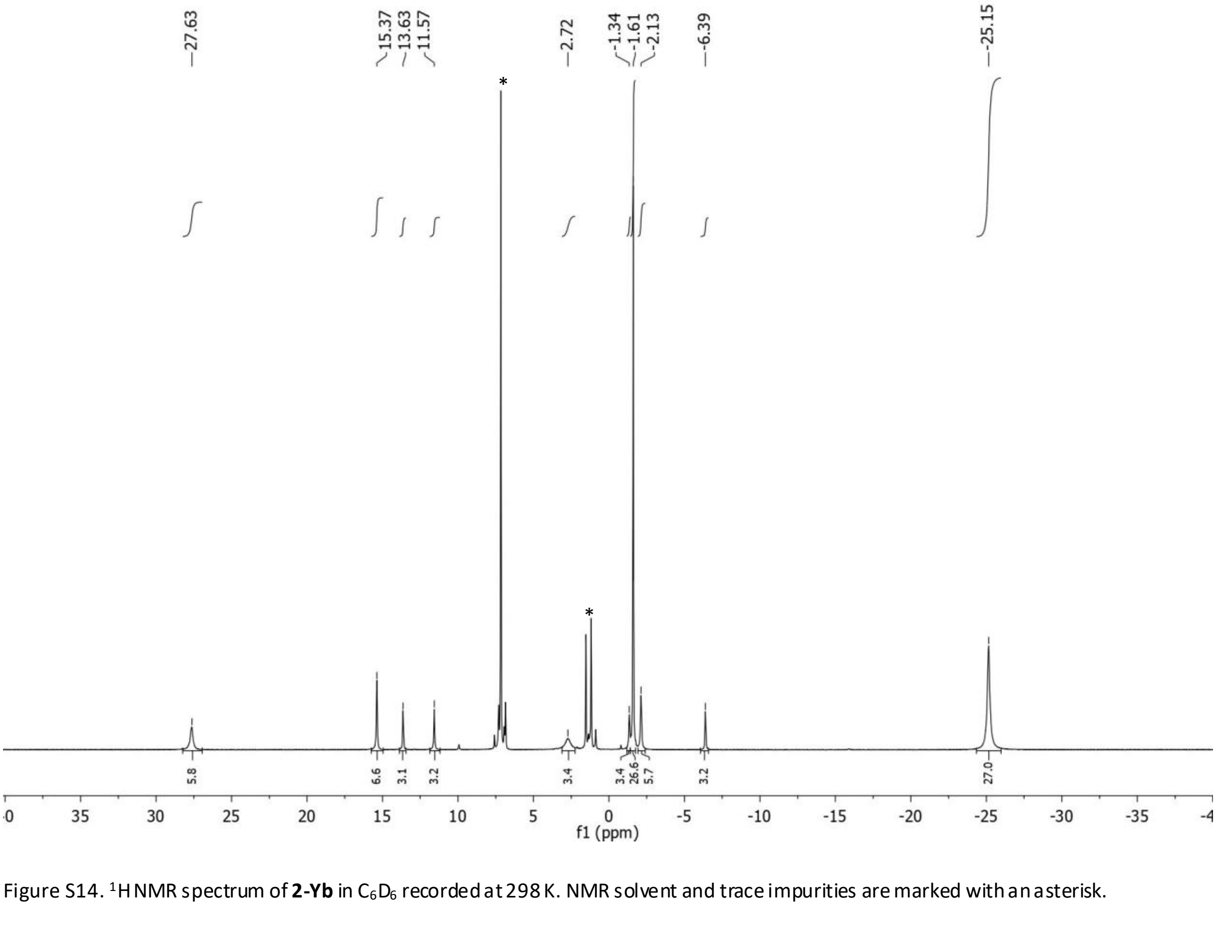

Figure S14. ^{1}H NMR spectrum of **2-Yb** in C_6D_6 recorded at 298 K. NMR solvent and trace impurities are marked with an asterisk.

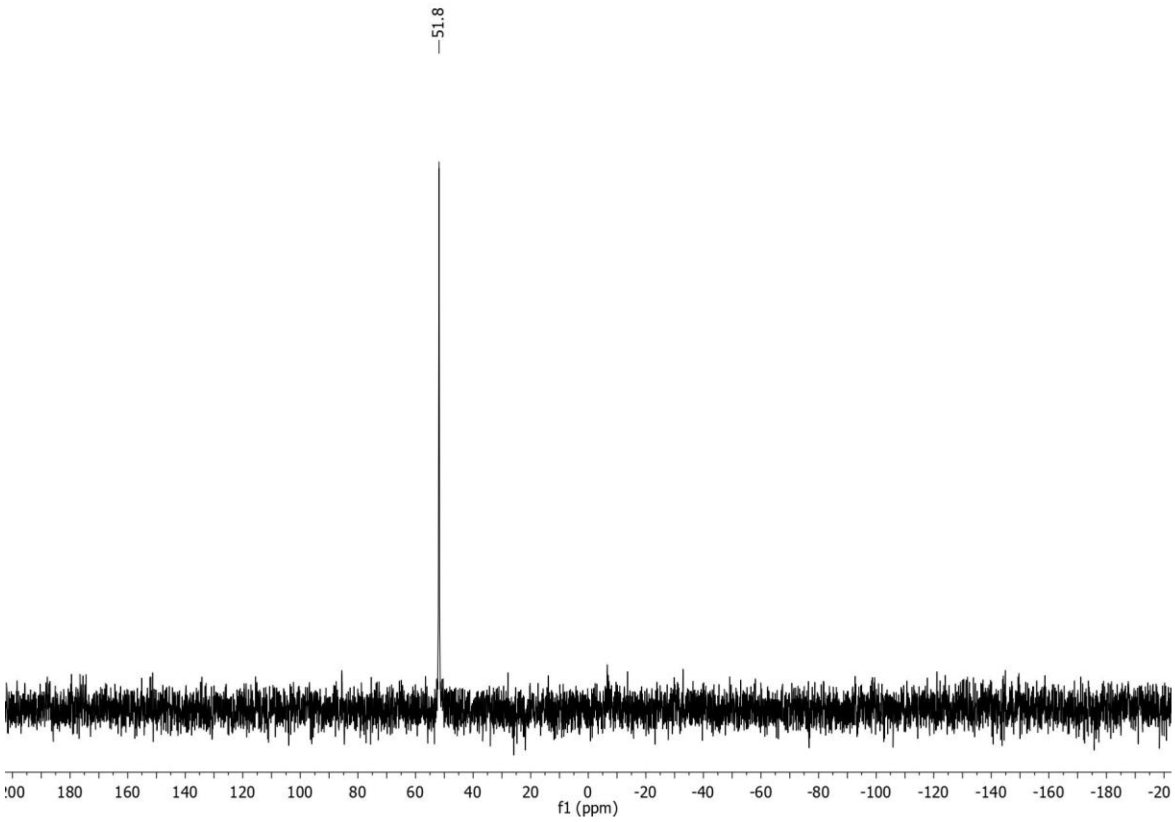

Figure S15. $^{31}P\{^{1}H\}$ NMR spectrum of **2-Yb** in C_6D_6 recorded at 298 K.

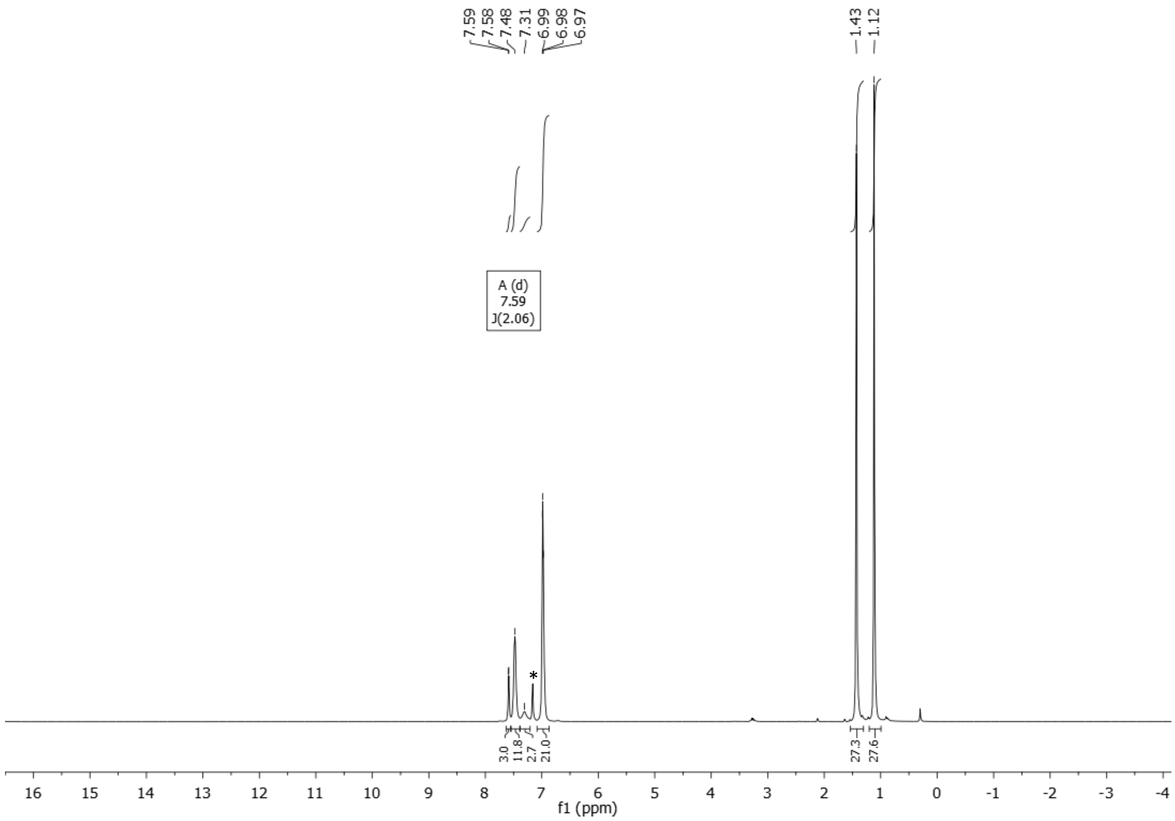

Figure S16. ^{1}H NMR spectrum of **3** in C_6D_6 recorded at 298 K. NMR solvent is marked with an asterisk.

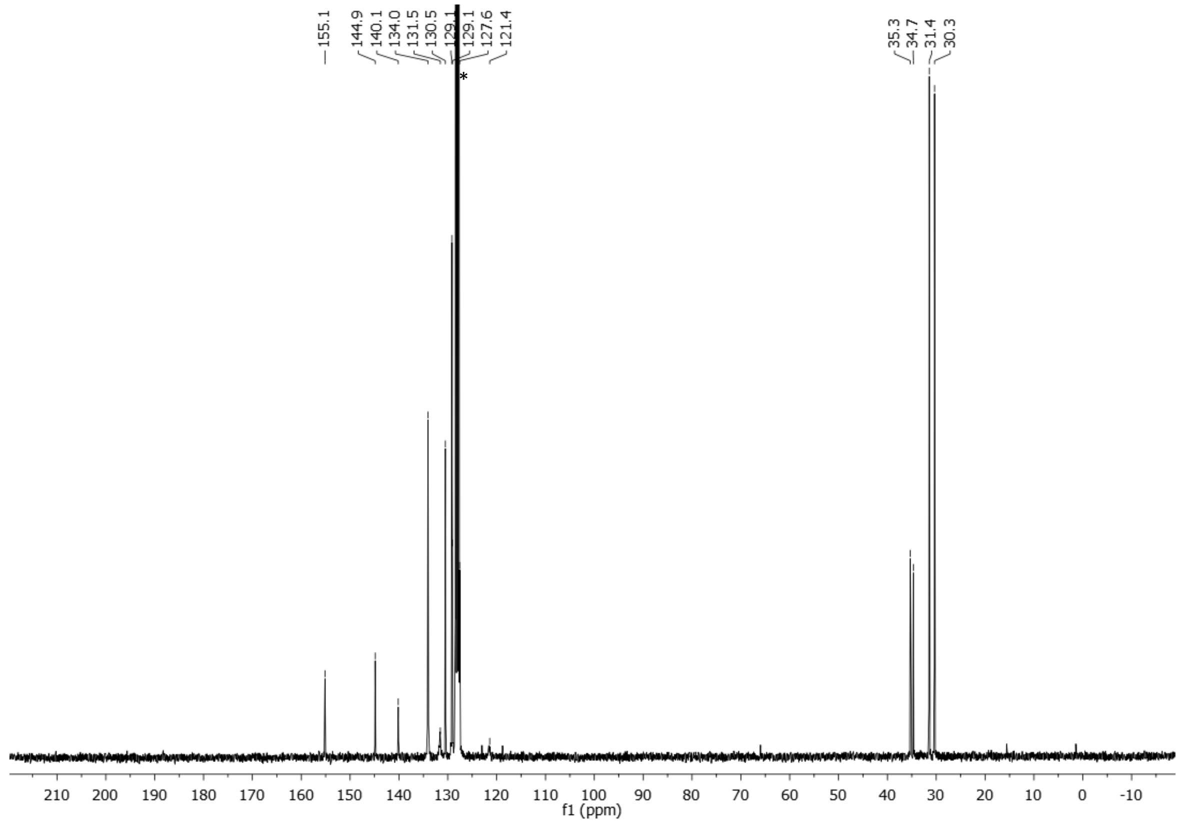

Figure S17. $^{13}C\{^{1}H\}$ NMR spectrum of **3** in C_6D_6 recorded at 298 K. NMR solvent is marked with an asterisk.

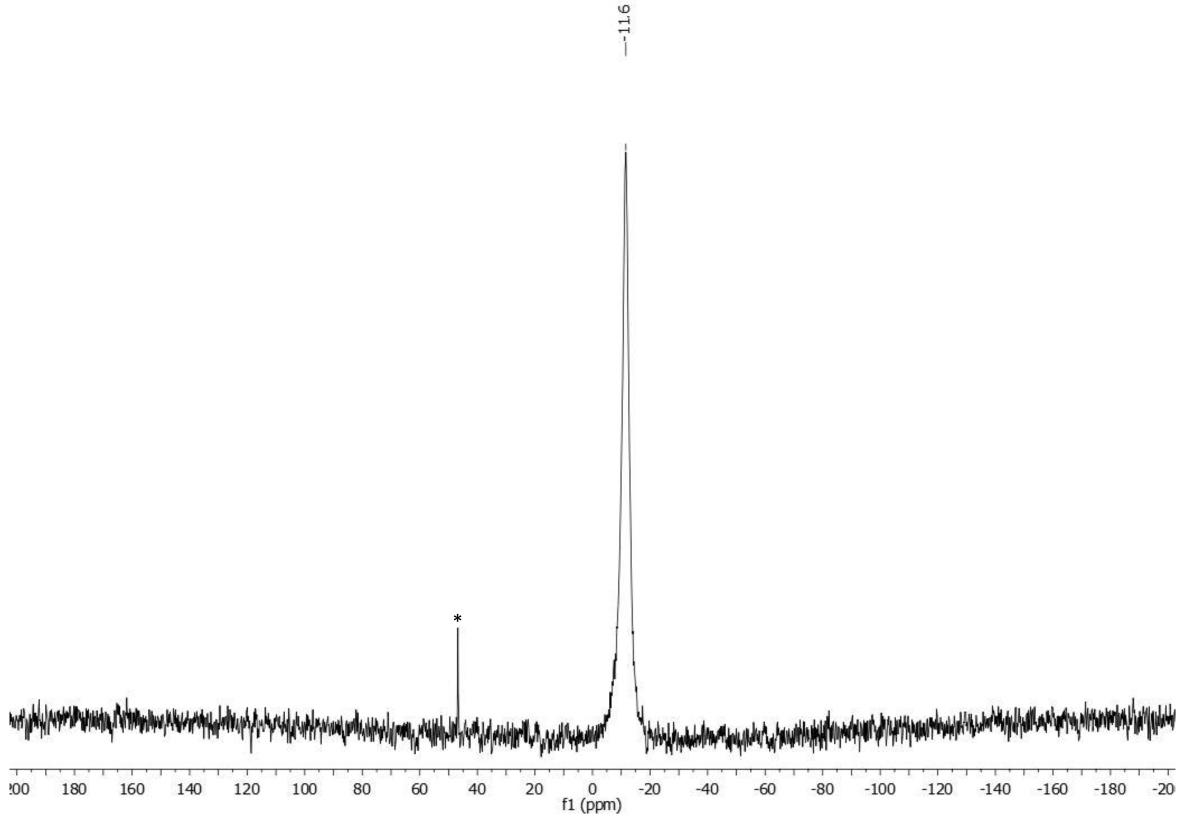

Figure S18. $^{31}P\{^{1}H\}$ NMR spectrum of **3** in C_6D_6 recorded at 298 K. Trace impurities are marked with an asterisk.

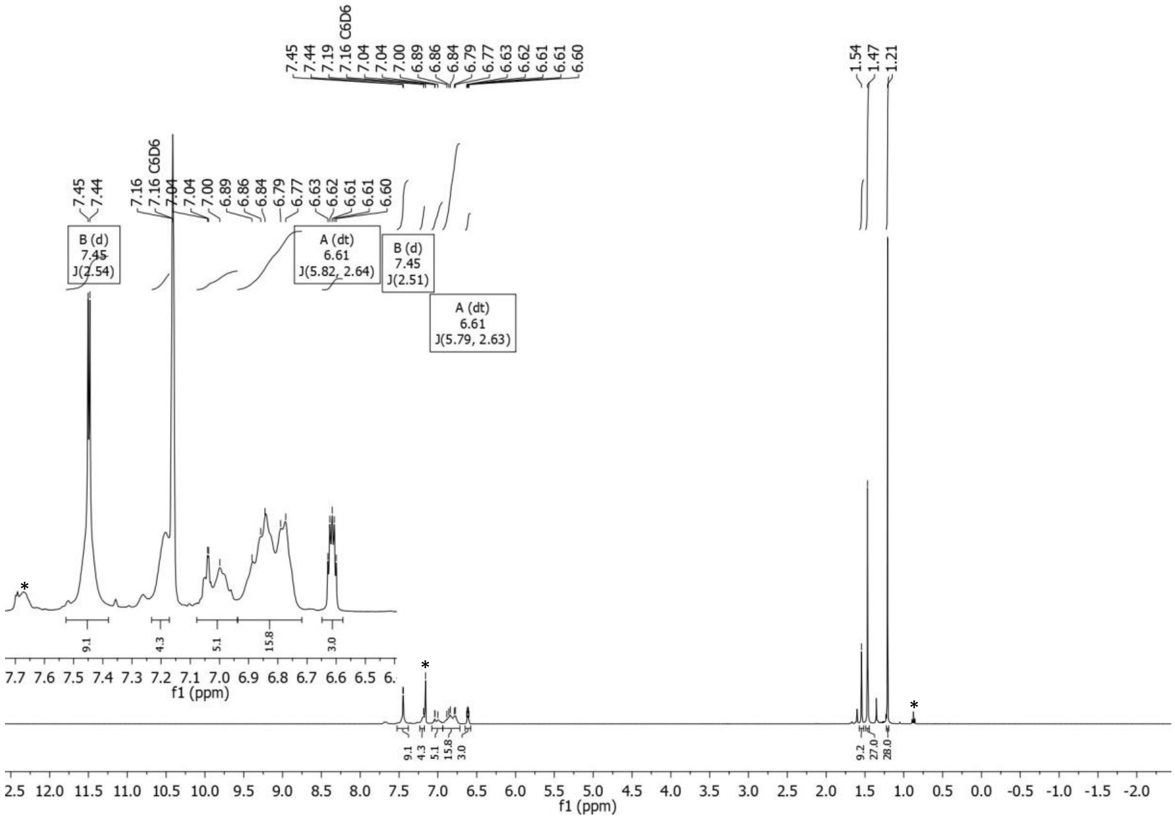

Figure S19. ^{1}H NMR spectrum of **4-La** in C_6D_6 recorded at 298 K. NMR solvent and trace impurities are marked with an asterisk.

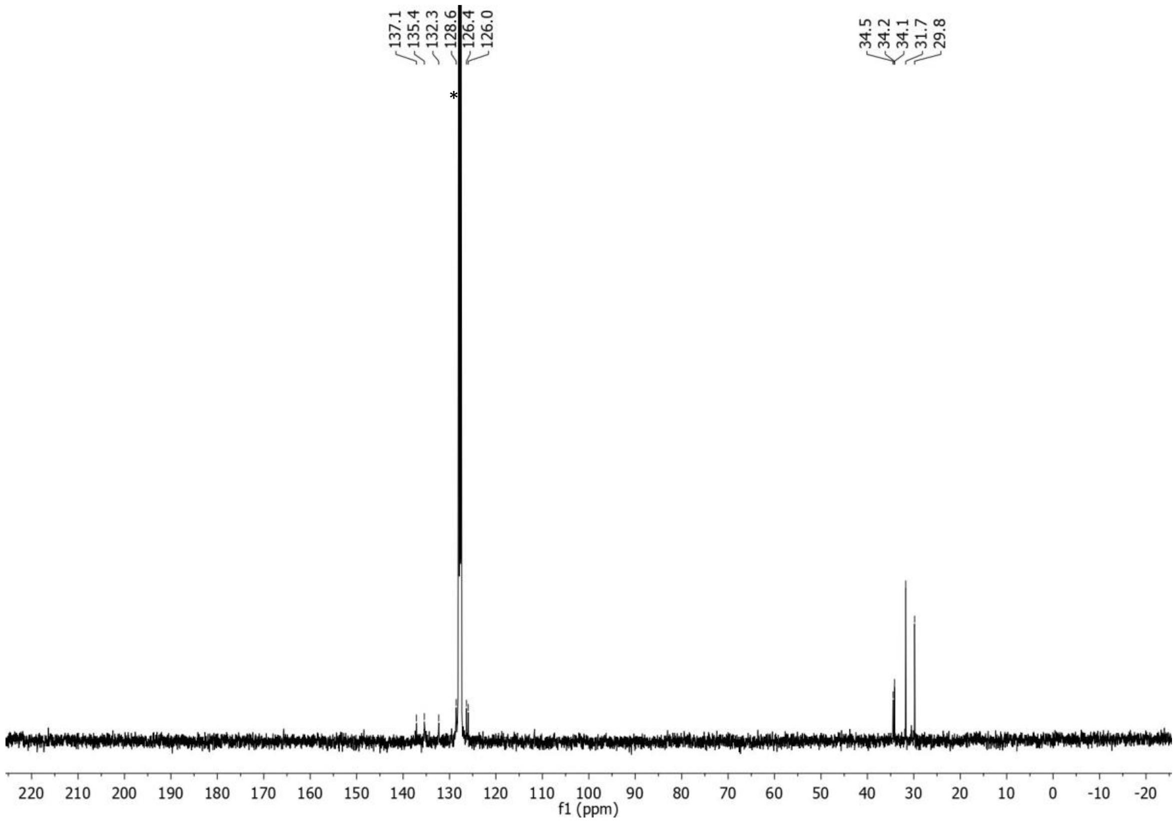

Figure S20. $^{13}C\{^{1}H\}$ NMR spectrum of **4-La** in C_6D_6 recorded at 298 K. NMR solvent is marked with an asterisk.

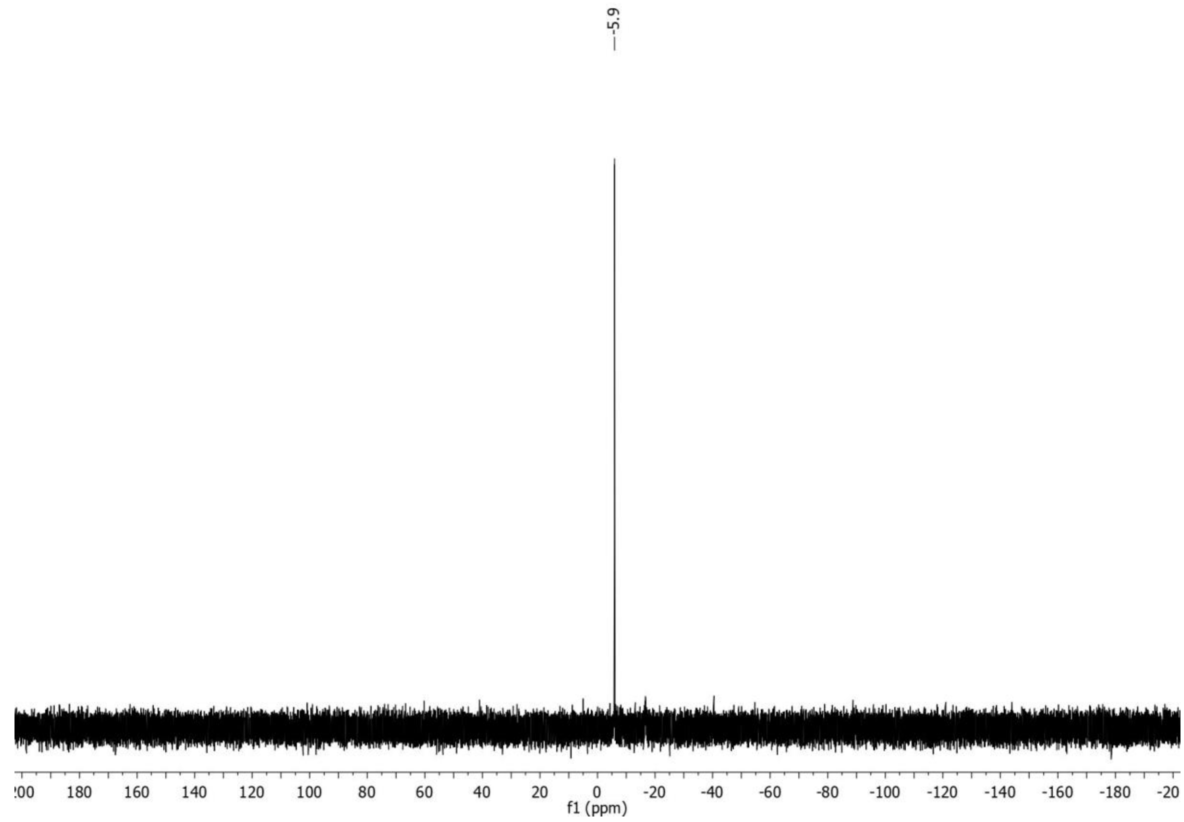

Figure S21. $^{31}P\{^{1}H\}$ NMR spectrum of **4-La** in C_6D_6 recorded at 298 K.

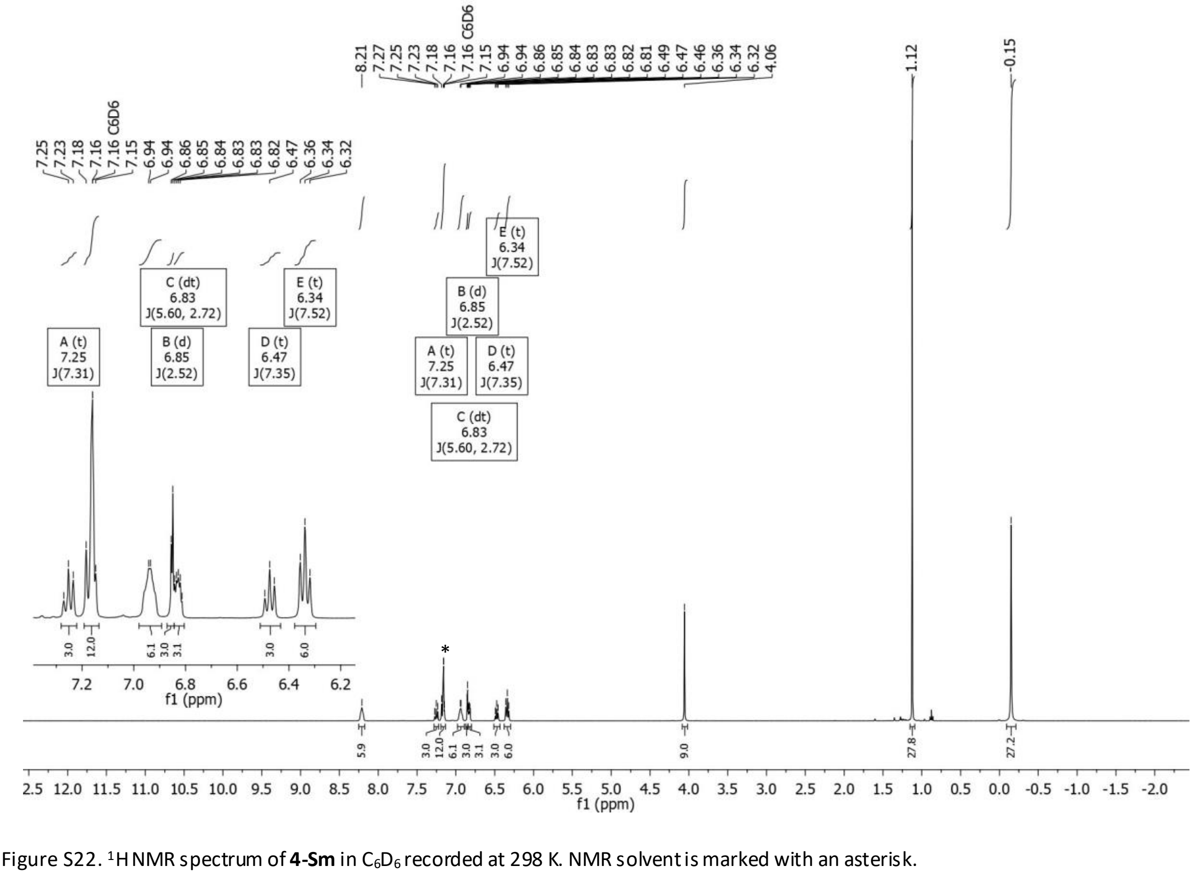

Figure S22. ^{1}H NMR spectrum of **4-Sm** in C_6D_6 recorded at 298 K. NMR solvent is marked with an asterisk.

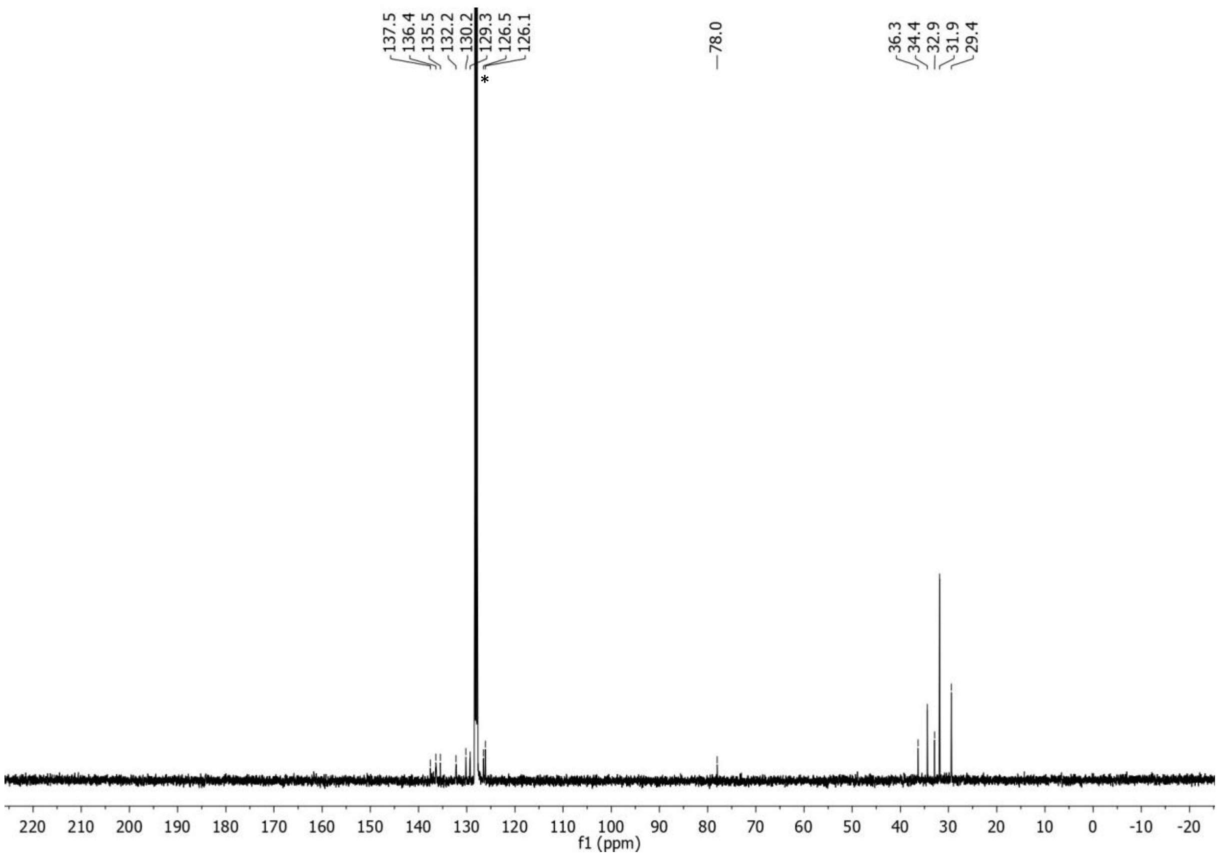

Figure S23. $^{13}C\{^{1}H\}$ NMR spectrum of **4-Sm** in C_6D_6 recorded at 298 K. NMR solvent is marked with an asterisk.

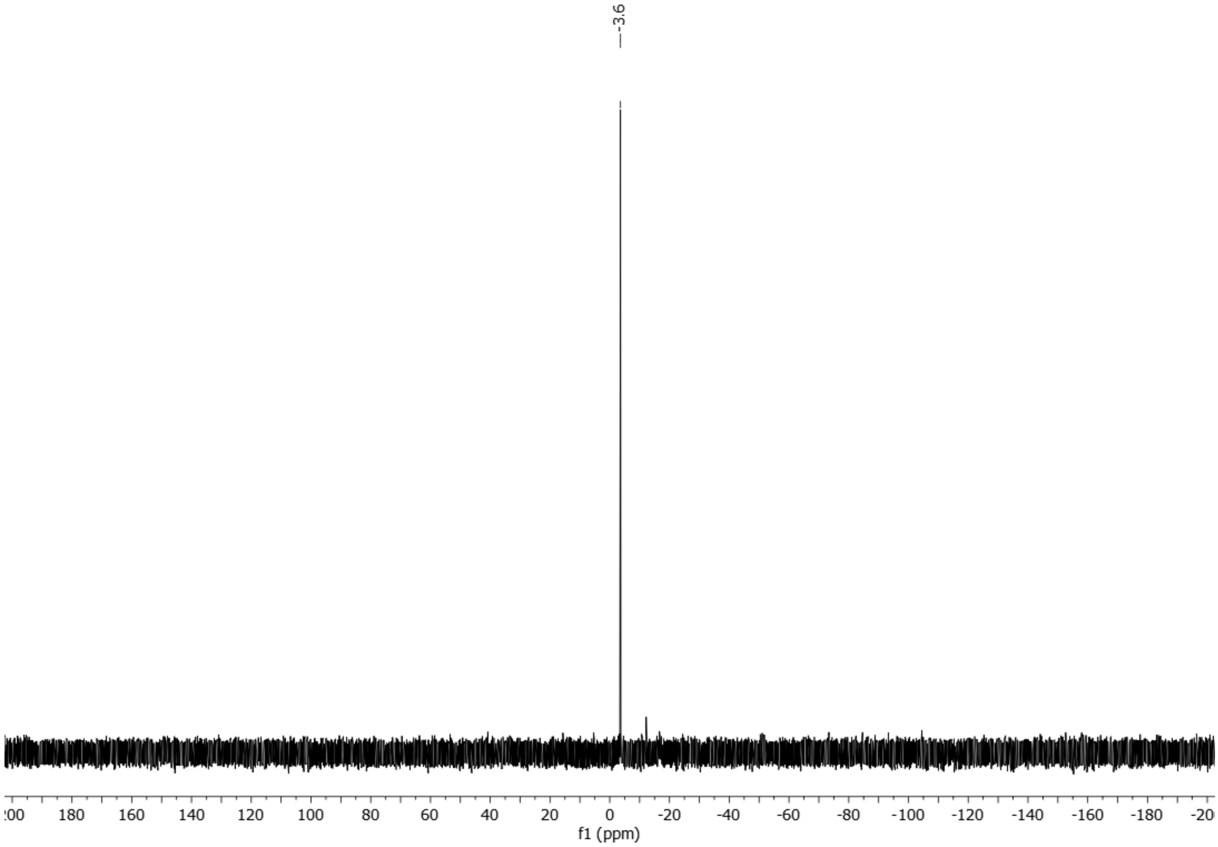

Figure S24. $^{31}P\{^{1}H\}$ NMR spectrum of **4-Sm** in C_6D_6 recorded at 298 K.

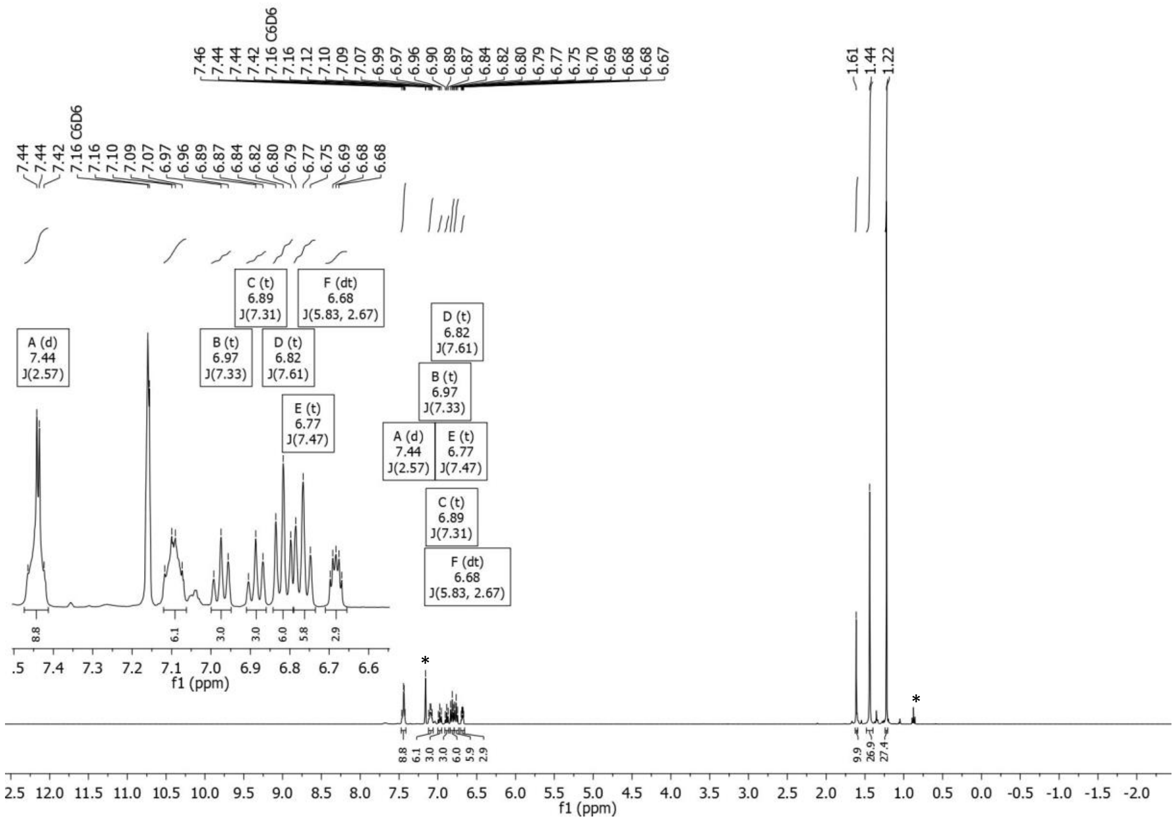

Figure S25. ^{1}H NMR spectrum of **4-Y** in C_6D_6 recorded at 298 K. NMR solvent and trace impurities are marked with an asterisk.

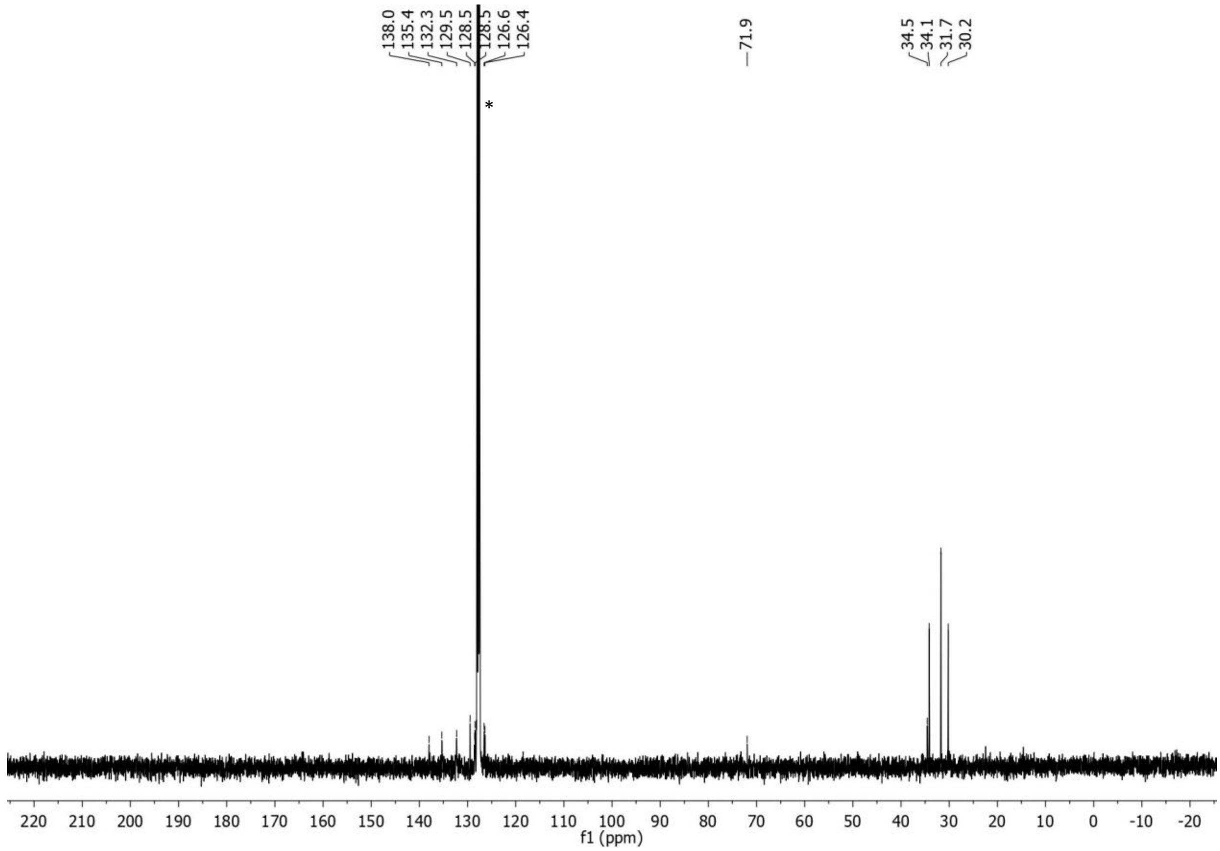

Figure S26. $^{13}C\{^{1}H\}$ NMR spectrum of **4-Y** in C_6D_6 recorded at 298 K. NMR solvent is marked with an asterisk.

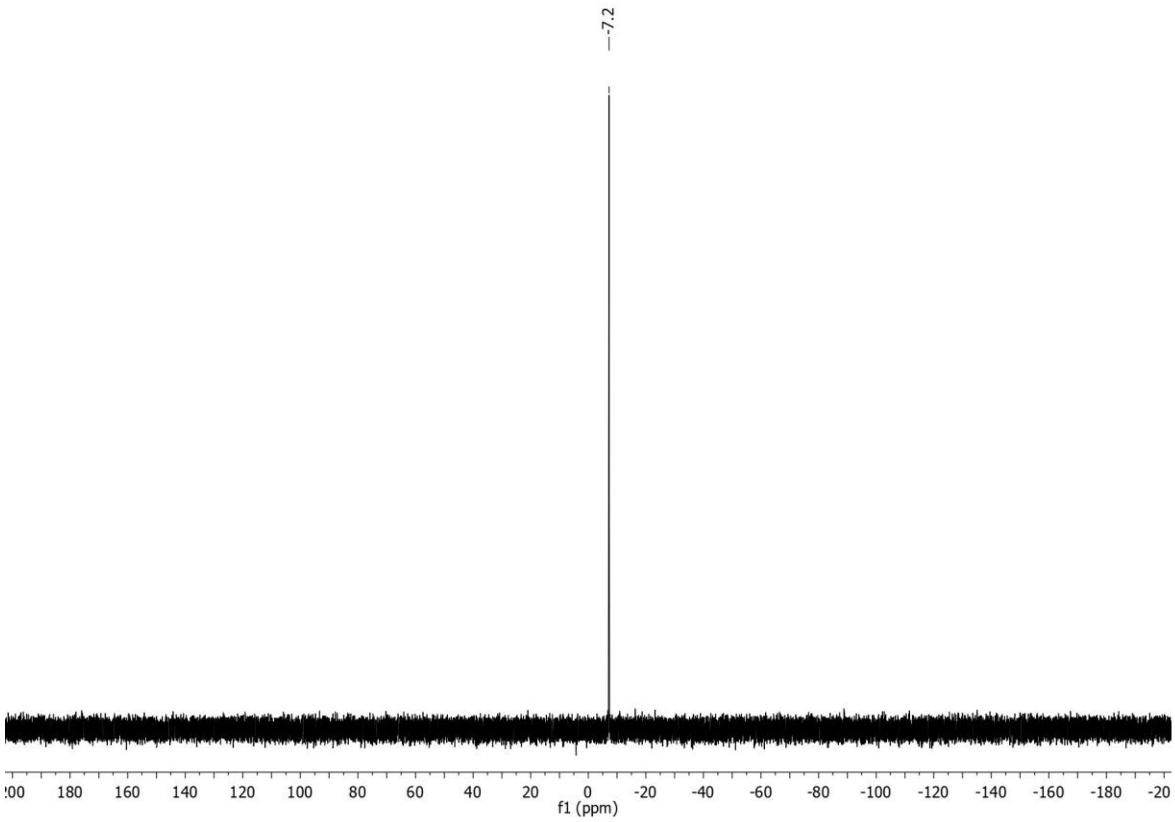

Figure S27. $^{31}P\{^{1}H\}$ NMR spectrum of **4-Y** in C_6D_6 recorded at 298 K.

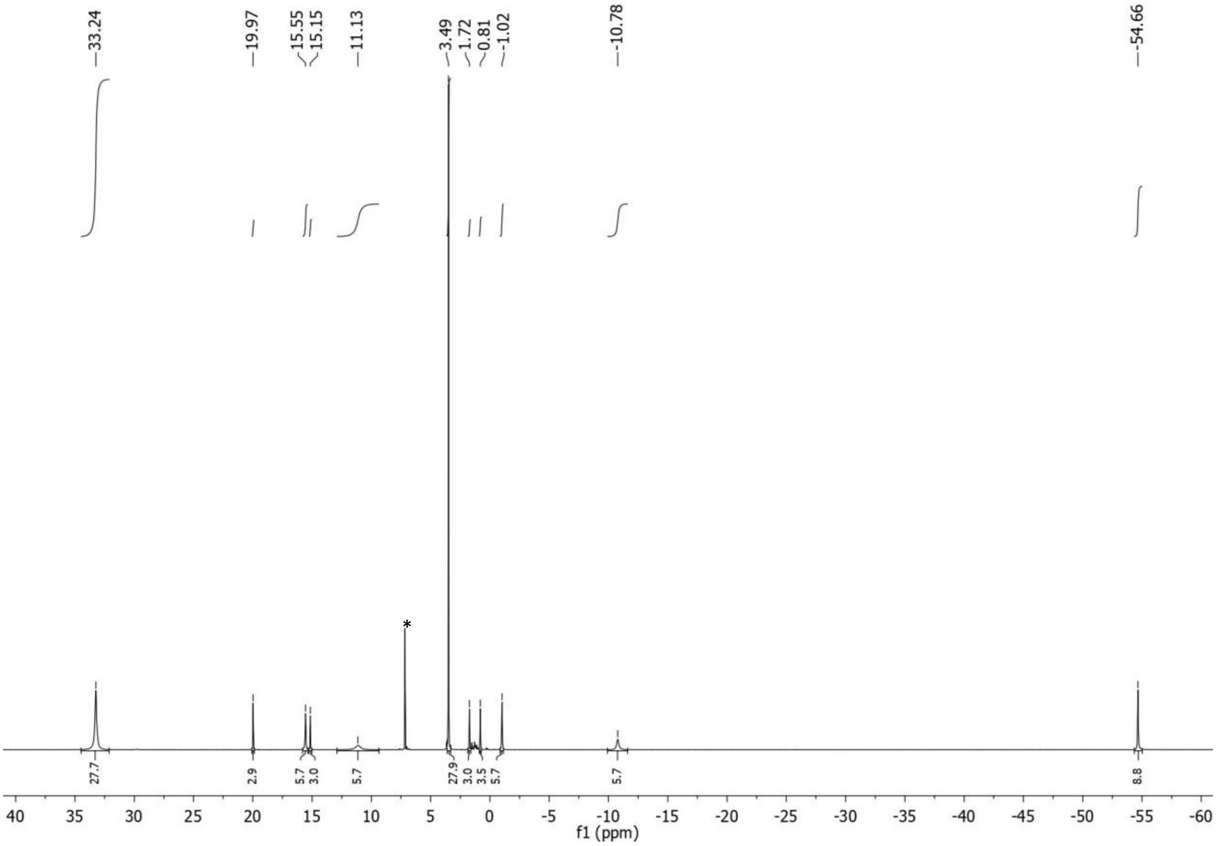

Figure S28. ^{1}H NMR spectrum of **4-Yb** in C_6D_6 recorded at 298 K. NMR solvent is marked with an asterisk.

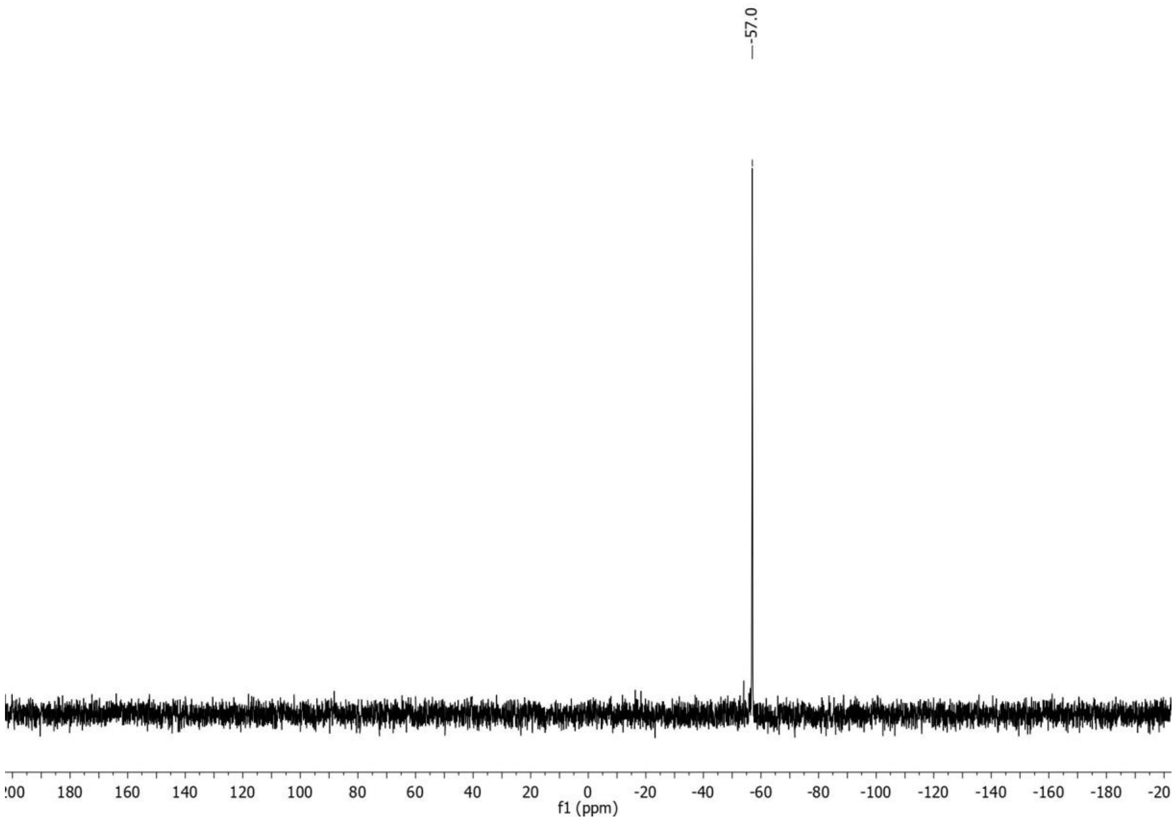

Figure S29. $^{31}P\{^{1}H\}$ NMR spectrum of **4-Yb** in C_6D_6 recorded at 298 K.

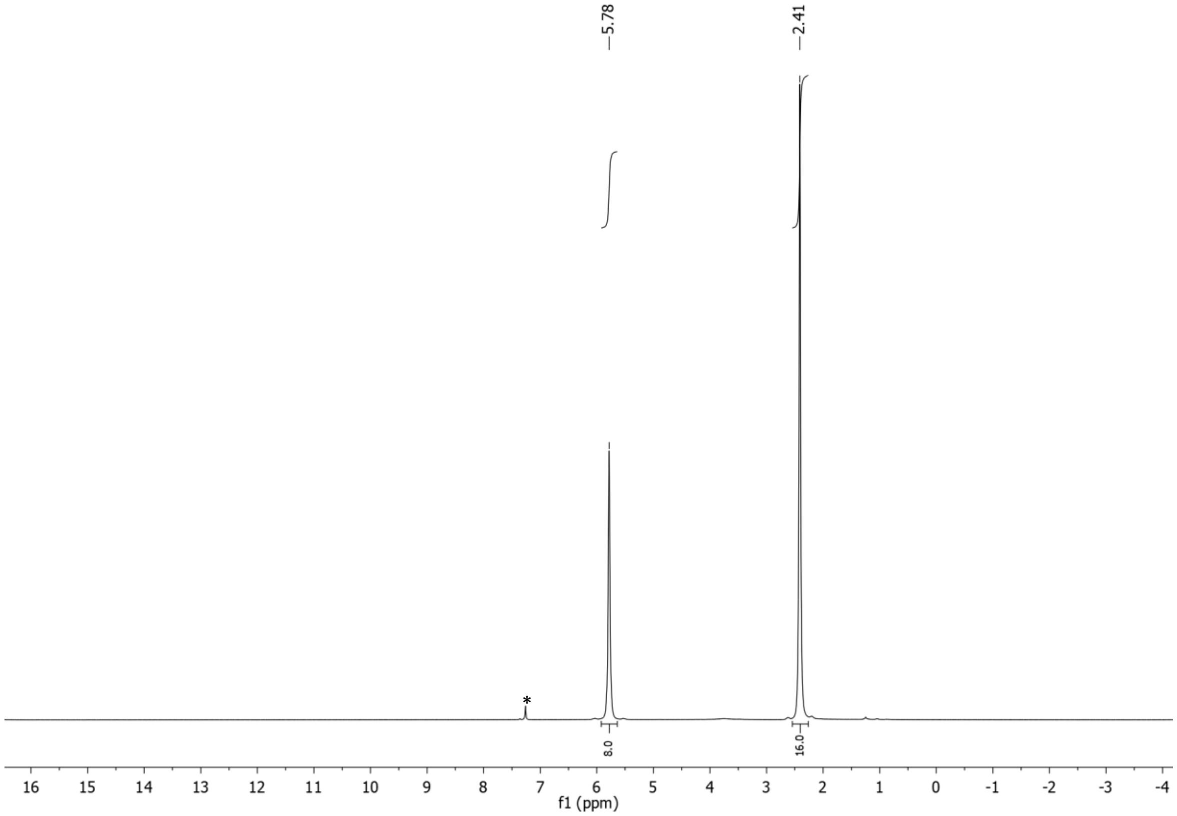

Figure S30. ^{1}H NMR spectrum of $(cod)_2Cu^{I}OTf$ in $CDCl_3$ recorded at 298 K. NMR solvent is marked with an asterisk.

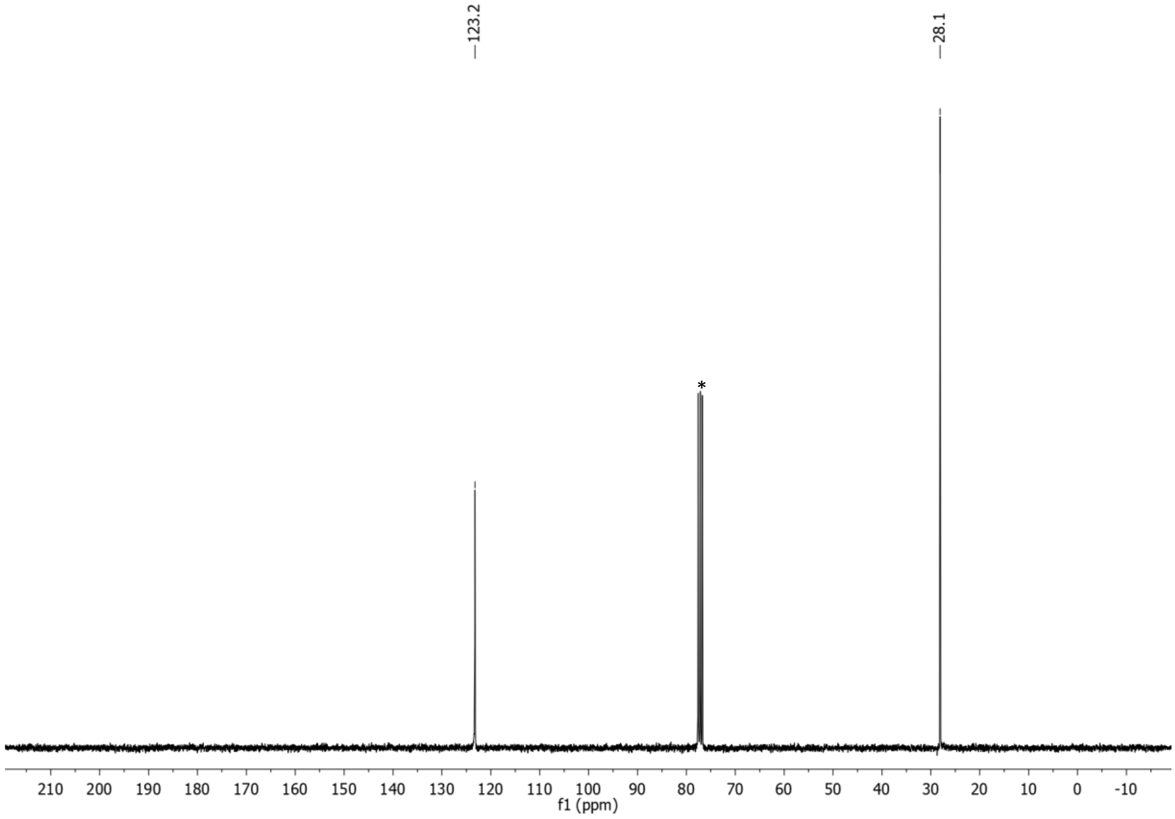

Figure S31. $^{13}C\{^{1}H\}$ NMR spectrum of $(cod)_2Cu^{I}OTf$ in $CDCl_3$ recorded at 298 K. NMR solvent is marked with an asterisk.

UV-vis Spectroscopy:

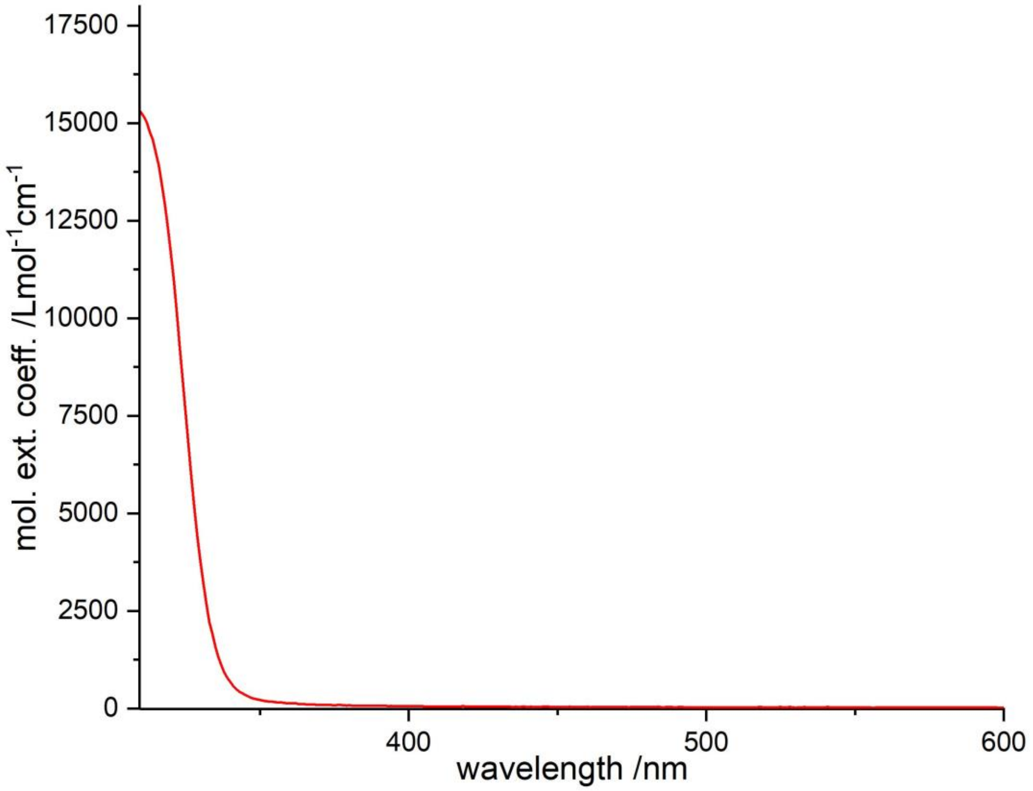

Figure S32. UV-vis spectrum of **2-La** recorded form a solution in toluene.

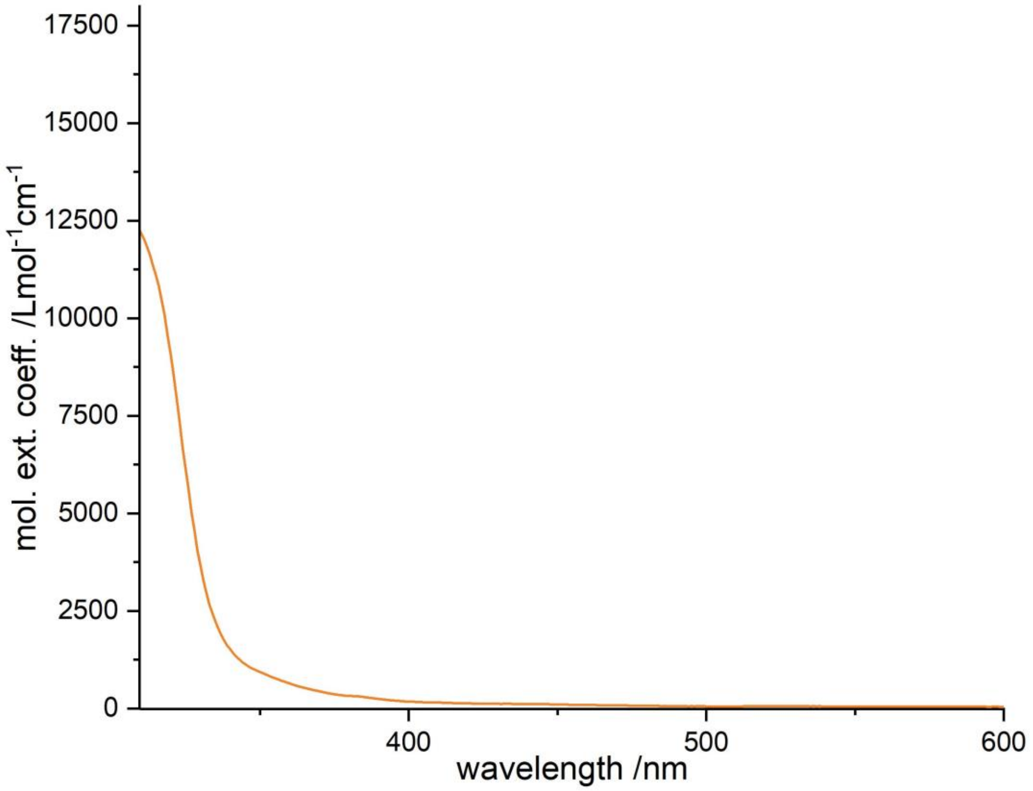

Figure S33. UV-vis spectrum of **2-Sm** recorded form a solution in toluene.

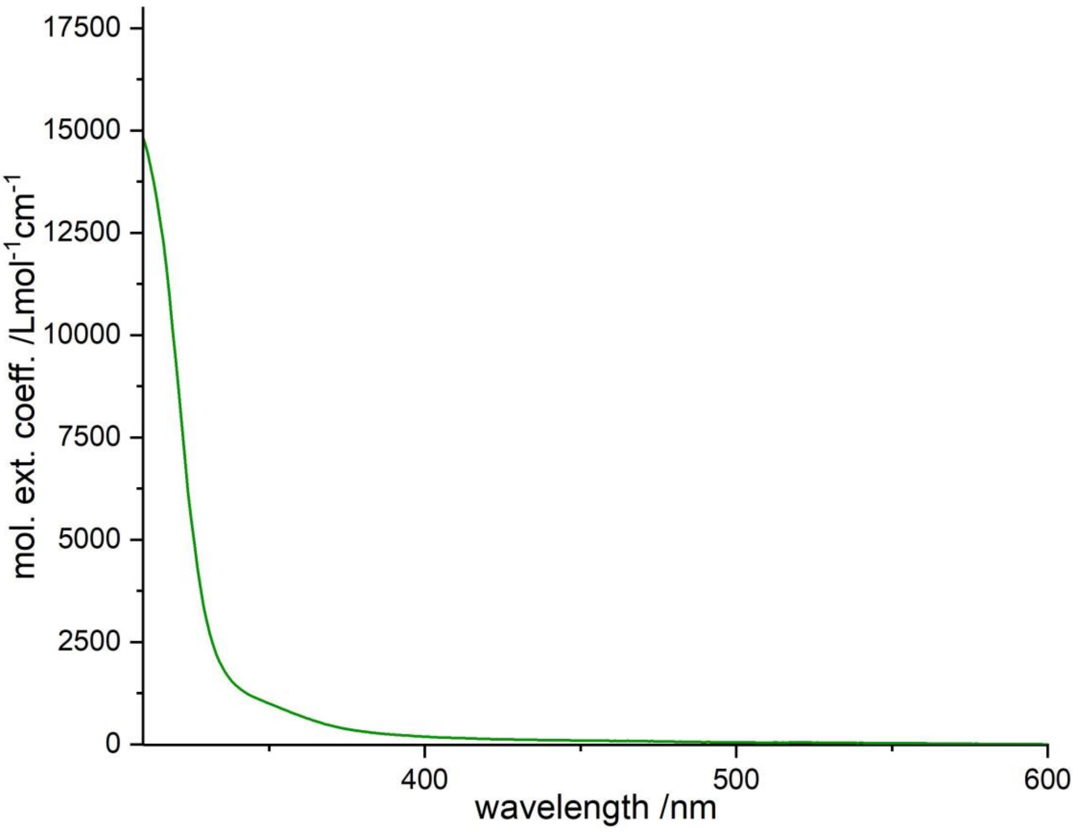

Figure S34. UV-vis spectrum of **2-Y** recorded form a solution in toluene.

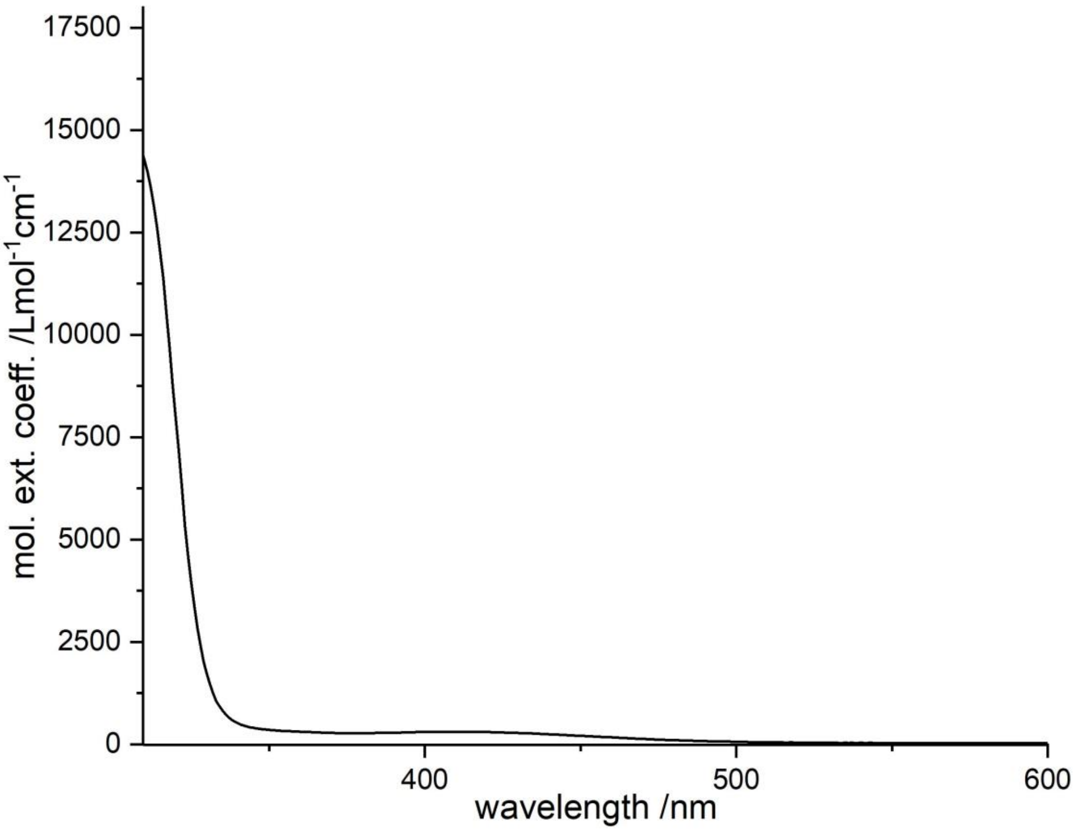

Figure S35. UV-vis spectrum of **2-Yb** recorded form a solution in toluene.

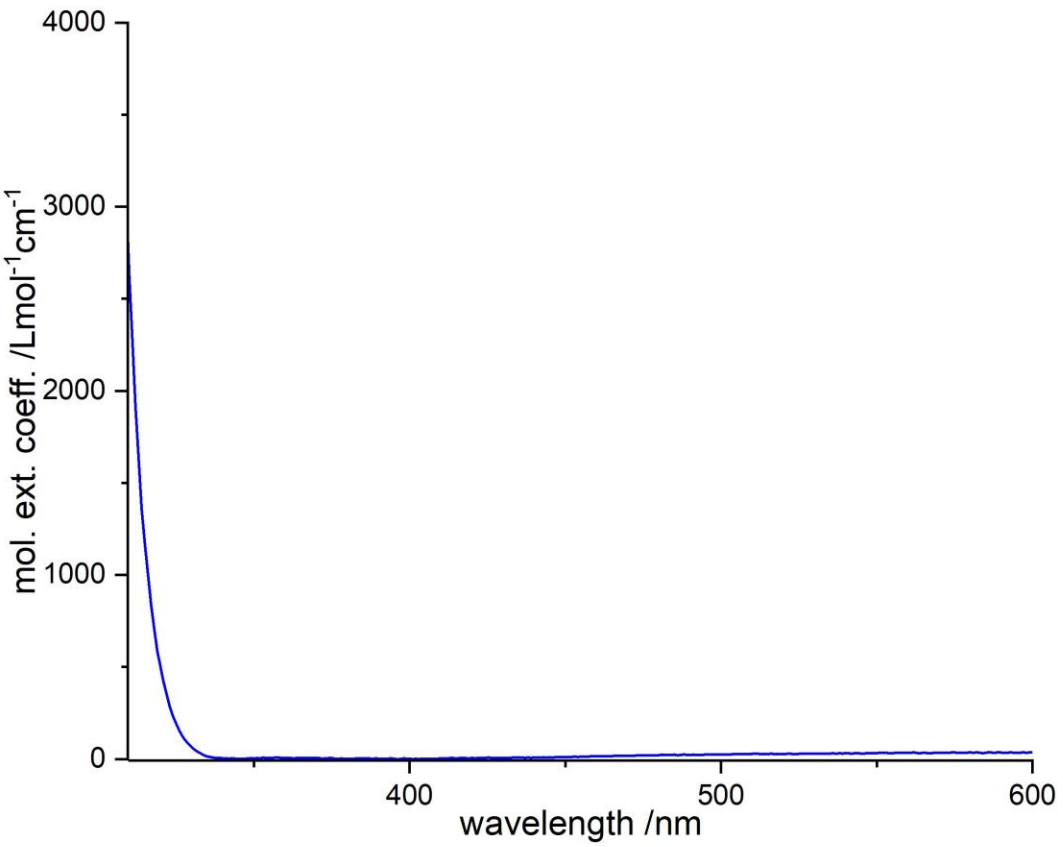

Figure S36. UV-vis spectrum of **3** recorded form a solution in toluene.

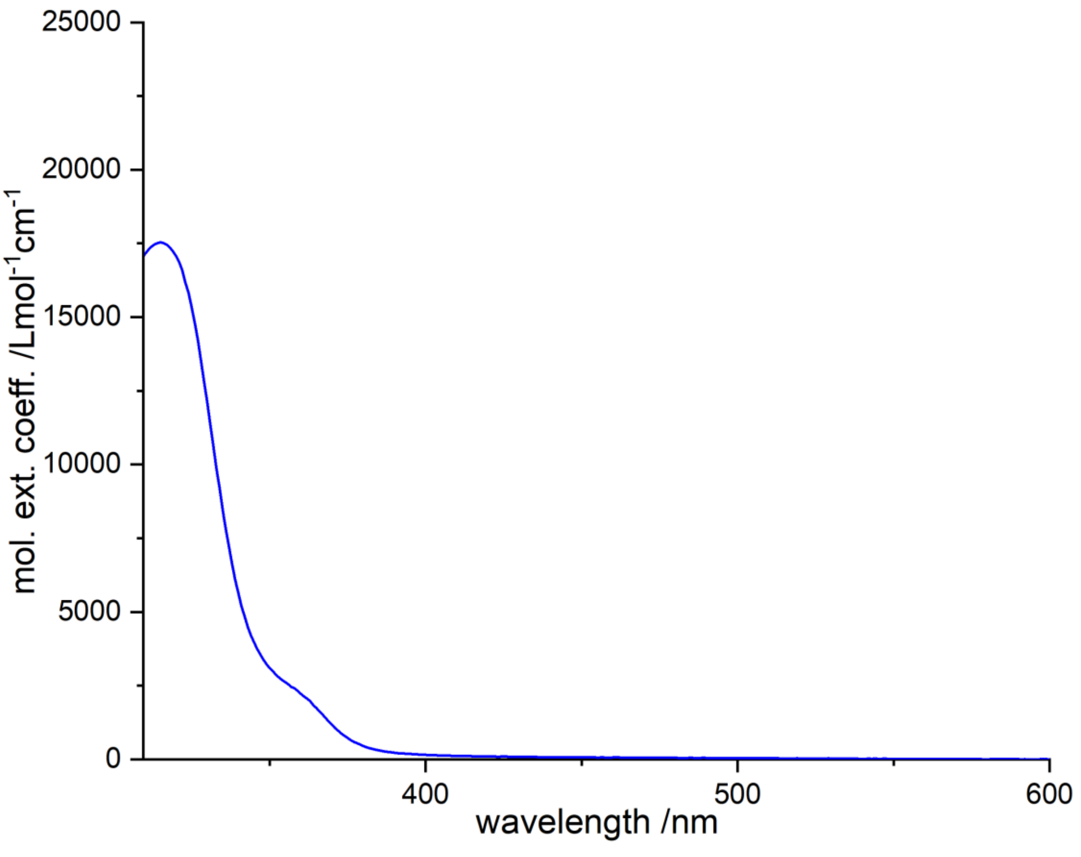

Figure S37. UV-vis spectrum of **4-La** recorded form a solution in toluene.

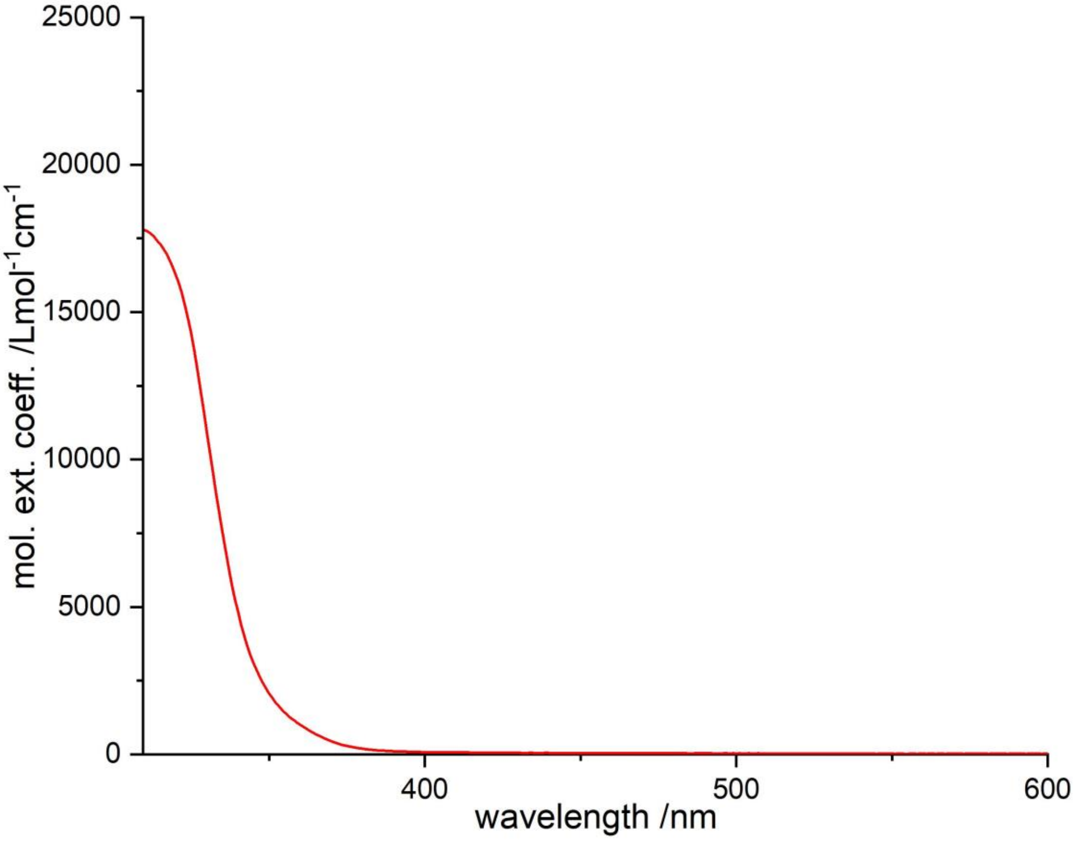

Figure S38. UV-vis spectrum of **4-Sm** recorded form a solution in toluene.

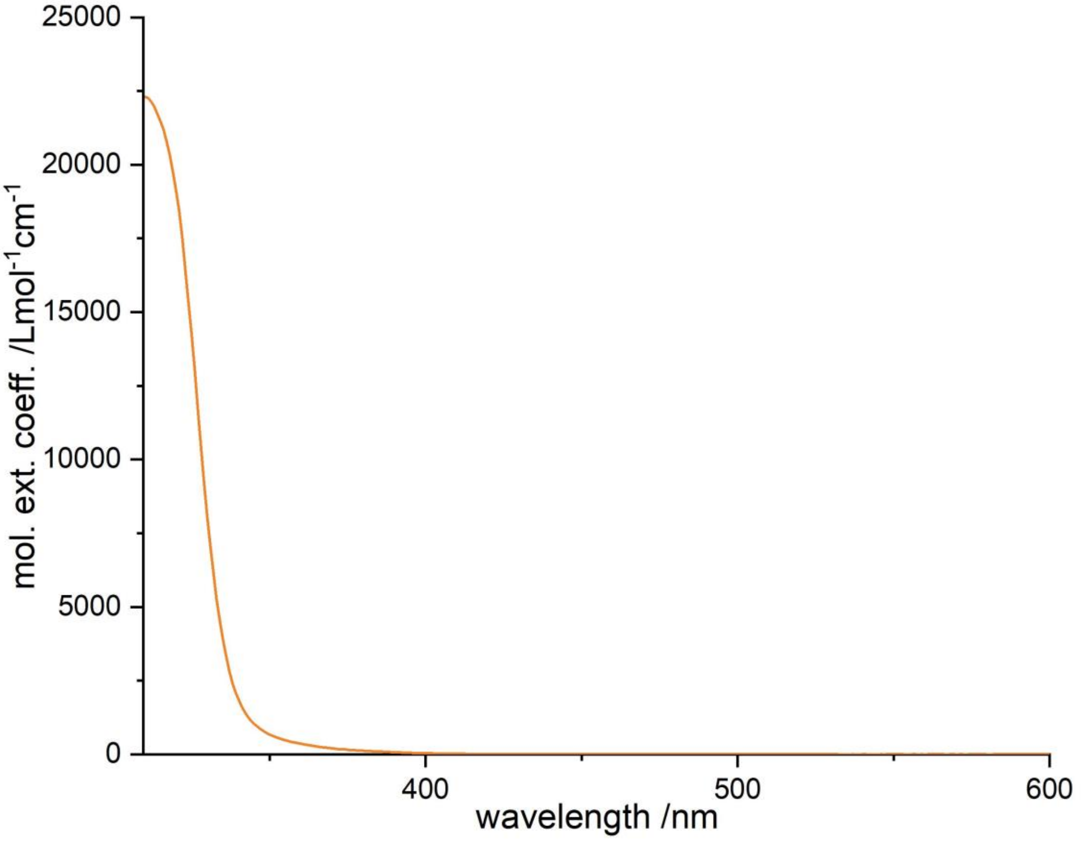

Figure S39. UV-vis spectrum of **4-Y** recorded form a solution in toluene.

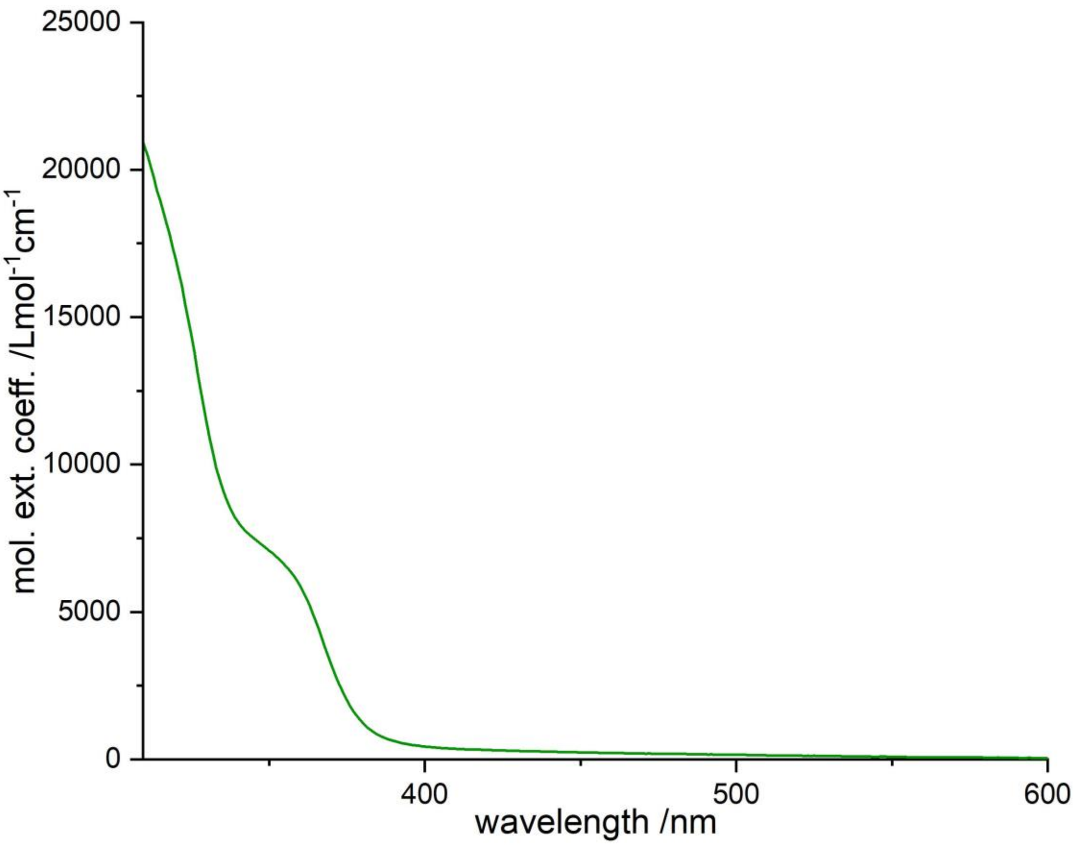

Figure S40. UV-vis spectrum of **4-Yb** recorded form a solution in toluene.

IR Spectroscopy:

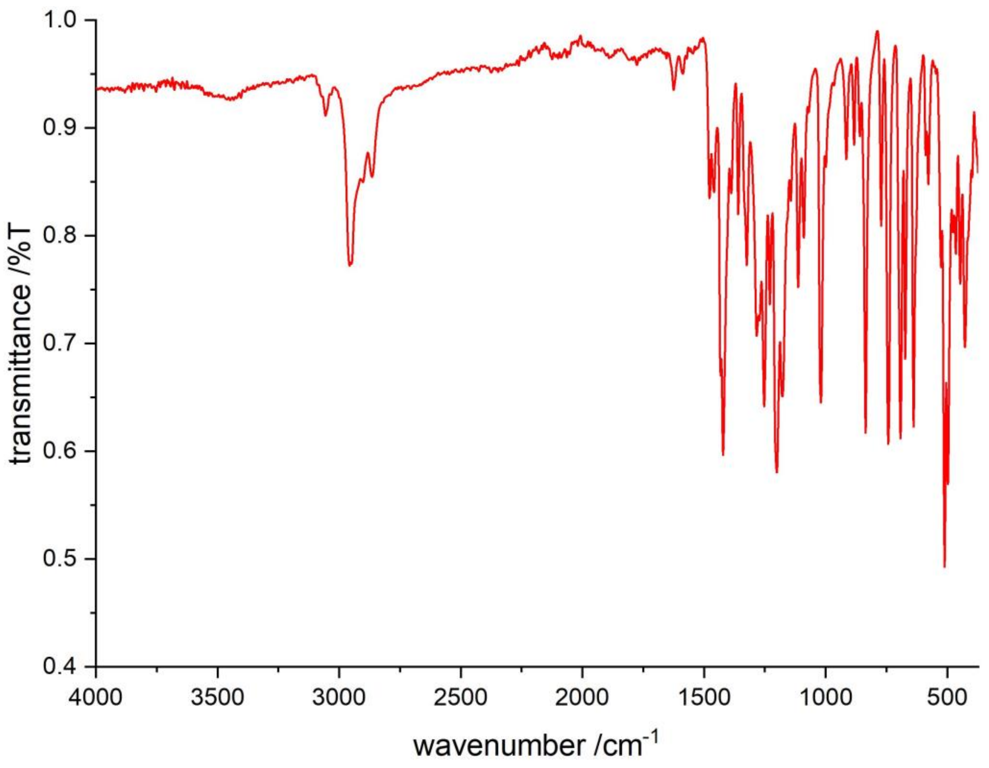

Figure S41. IR spectrum of **2-La** recorded neat from crystalline material using ATR.

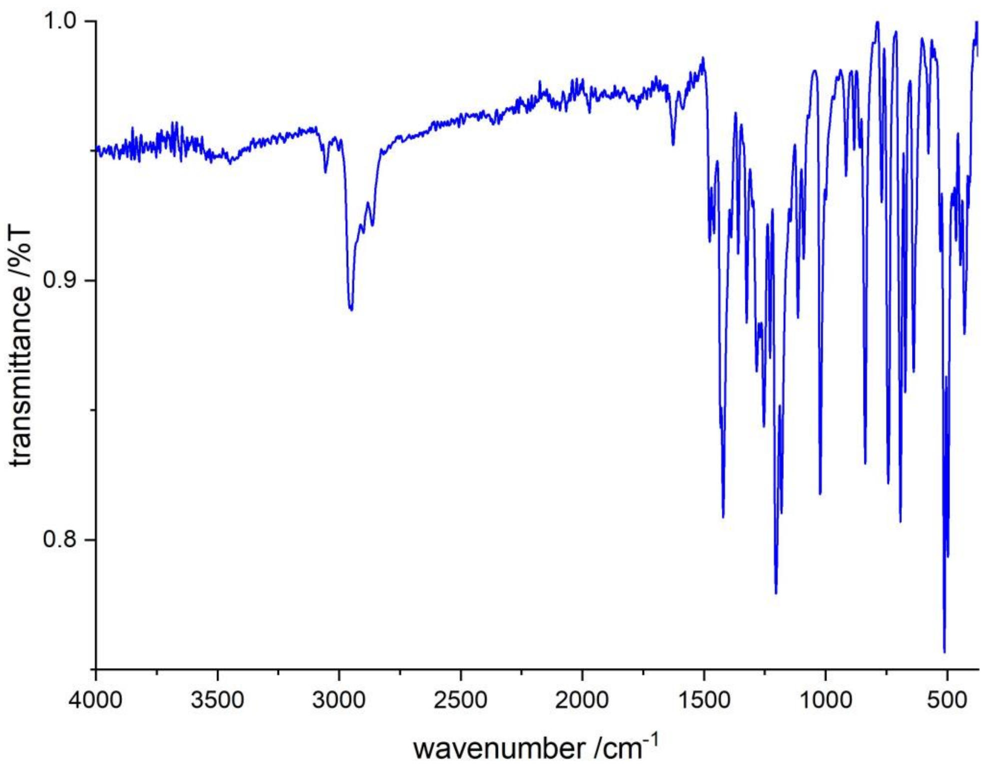

Figure S42. IR spectrum of **2-Sm** recorded neat from crystalline material using ATR.

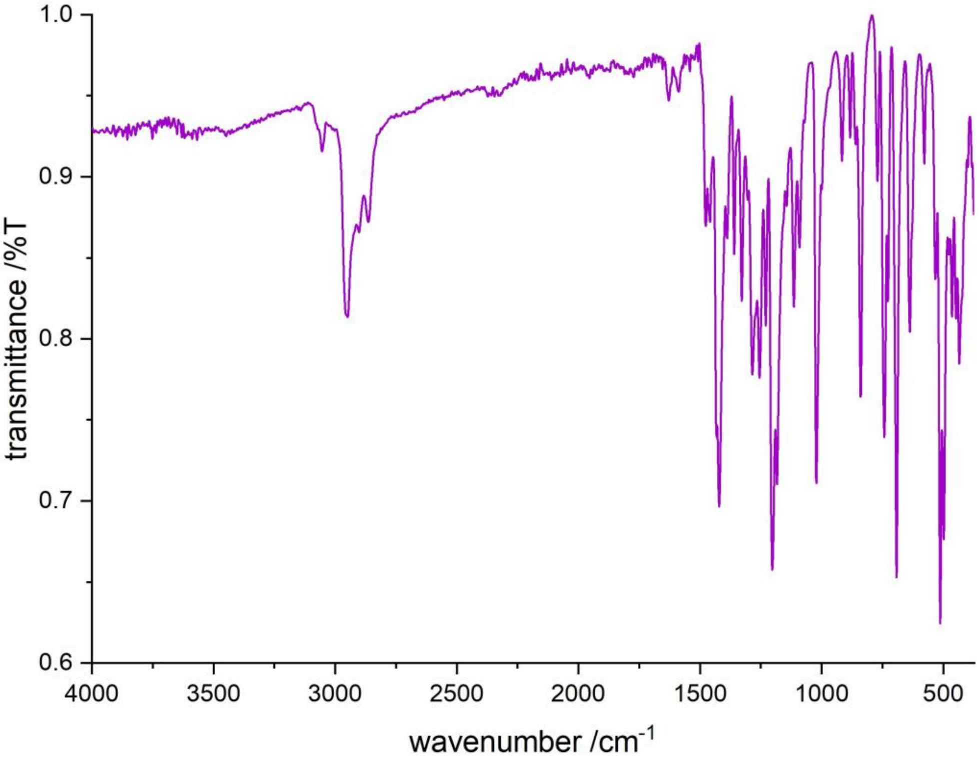

Figure S43. IR spectrum of **2-Y** recorded neat from crystalline material using ATR.

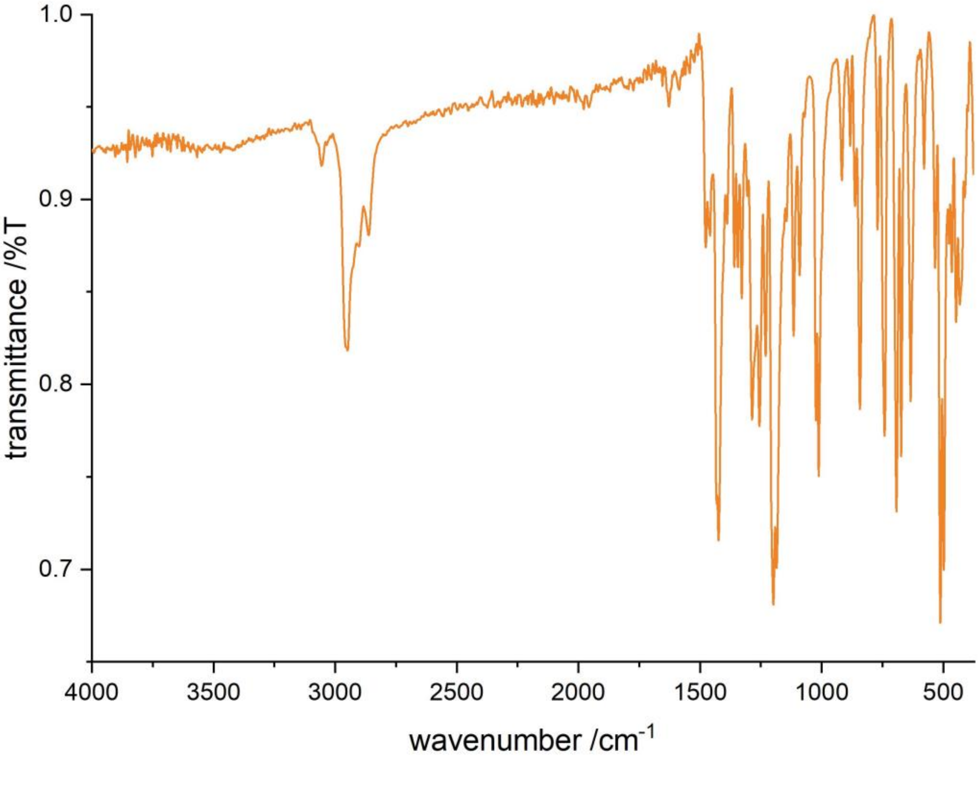

Figure S44. IR spectrum of **2-Yb** recorded neat from crystalline material using ATR.

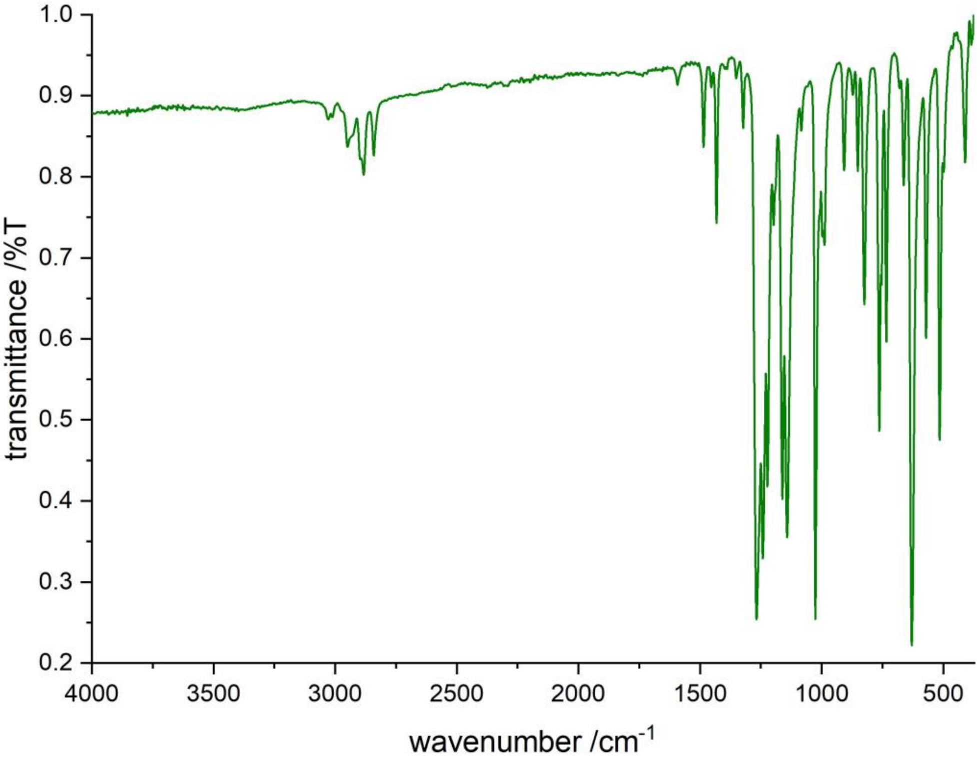

Figure S45. IR spectrum of **3** recorded neat from crystalline material using ATR.

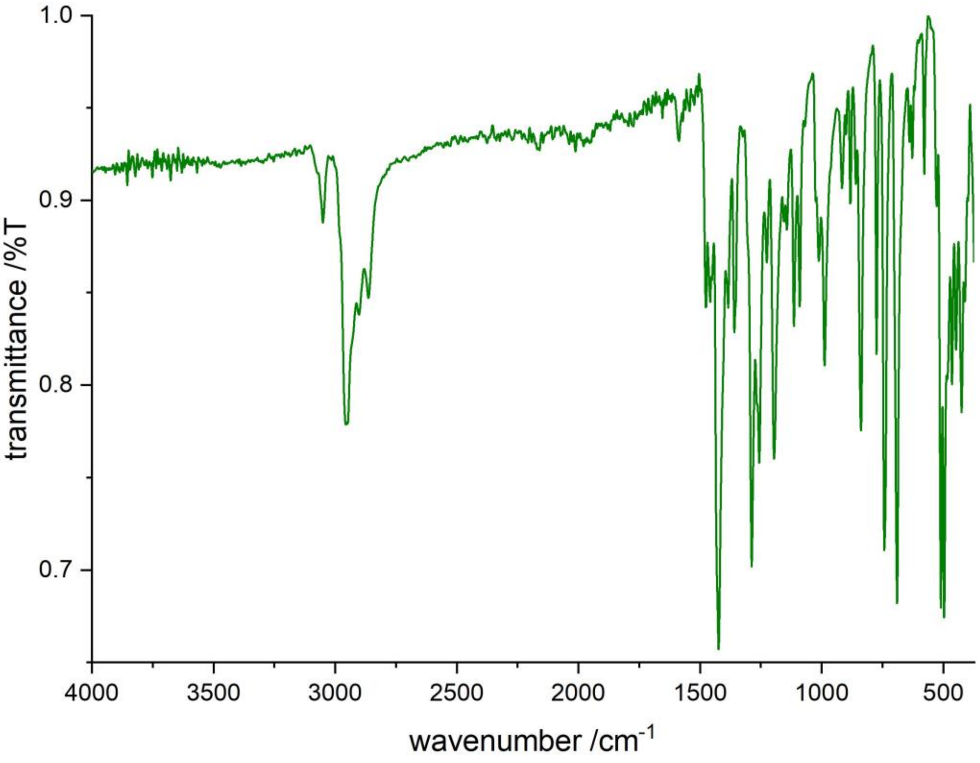

Figure S46. IR spectrum of **4-La** recorded neat from crystalline material using ATR.

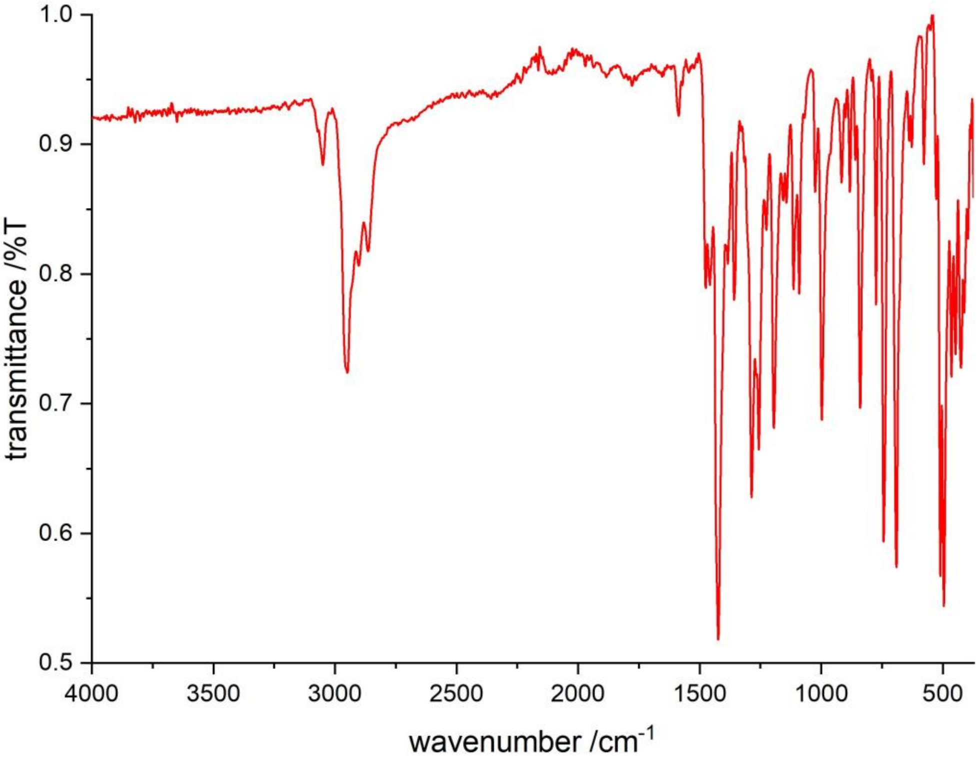

Figure S47. IR spectrum of **4-Sm** recorded neat from crystalline material using ATR.

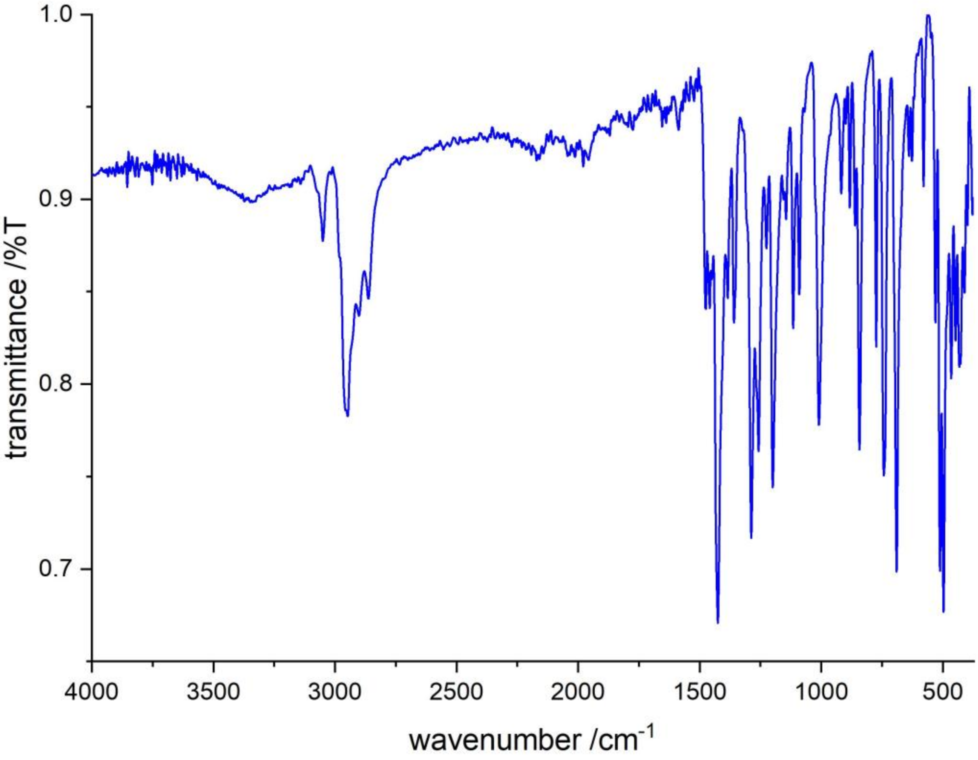

Figure S48. IR spectrum of **4-Y** recorded neat from crystalline material using ATR.

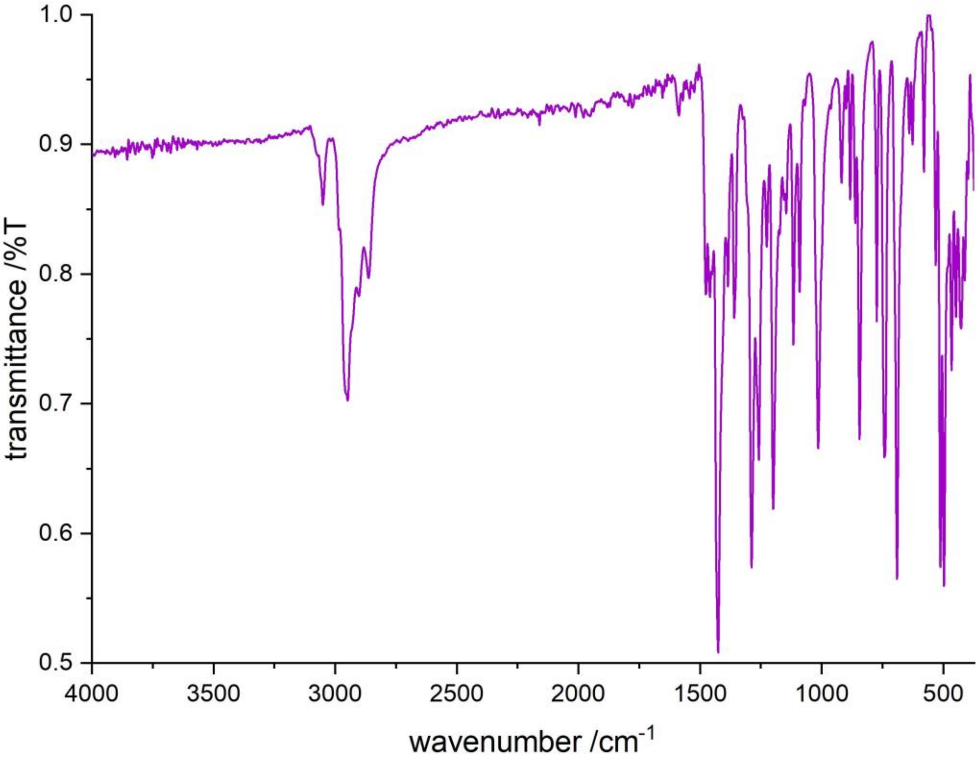

Figure S49. IR spectrum of **4-Yb** recorded neat from crystalline material using ATR.

Selected Derivatisation Attempts:

Treatment of 2-Y with $NaN(SiMe_3)_2$: A vial equipped with a stirrer bar was charged with **2-Y** (295 mg, 200 µmol) and sodium hexamethyldisilazide (37 mg, 200 µmol). The educts were taken up in toluene (4 ml) at ambient temperature and the stirred reaction mixture exhibited immediate formation of a white solid. The suspension was stirred for 18 h after which the solids were removed via filtration. The colourless solution was concentrated under reduced pressure and the NMR spectroscopic analysis of the remaining colourless solid (263 mg) exhibited decomposition into mononuclear compounds as indicated by the dominating presence of the yttrium complex **1-Y**.

Treatment of 2-Y with $LiNEt_2$: The experiment was performed in the same way as the former using **2-Y** (147 mg, 100 µmol), lithium diethylamide (8 mg, 0.1 mmol), and benzene as solvent for the reaction. After the workup the analysis of the isolated colourless solid (119 mg) exhibited NMR spectroscopic data characteristic for **1-Y**.

Treatment of 2-Y with LiMe: A vial equipped with a stirrer bar was charged with **2-Y** (147 mg, 100 µmol) and methyllithium (2 mg, 0.1 mmol). The educts were taken up in benzene (6 ml) at ambient temperature upon which the mixture turned yellow immediately. Within some further 30 min the reaction mixture darkened. When left standing without stirring a dark grey to black solid settled under a colourless solution. After 2h the solids were removed via filtration followed by evaporation of all volatiles. The NMR spectroscopic analysis of the remaining off-white solid (87 mg) exhibited a range of multiple different products of which only **1-Y** was identifiable due to the characteristic doublet in ^{31}P NMR spectrum. Any attempts to separate and identify other components of the mixture failed.

Treatment of 2-Y with KBn: A vial equipped with a stirrer bar was charged with **2-Y** (147 mg, 100 µmol) and THF (5 ml) and a second vial was charged with benzylpotassium (14 mg, 0.10 mmol) and THF (3 ml) and both solutions cooled to -35 °C in glovebox freezer. Then the solution of **2-Y** was placed in a cold metal block of the same temperature and under stirring the red solution of benzylpotassium was added dropwise. Within 10 min of further stirring in the cooling block after the addition the reaction mixture formed a dark grey precipitate. After 2 h all volatiles were evaporated under reduced pressure and the dark grey residue extracted with toluene. The extract was concentrated under reduced pressure yielding an off-white solid (98 mg). The NMR spectroscopic analysis exhibited a wide range of products and any attempts to isolate individual components were unsuccessful.
